# Whole-genome Duplication Reshaped Adaptive Evolution in A Relict Plant Species, *Cyclocarya paliurus*

**DOI:** 10.1016/j.gpb.2023.02.001

**Published:** 2023-02-11

**Authors:** Yinquan Qu, Xulan Shang, Ziyan Zeng, Yanhao Yu, Guoliang Bian, Wenling Wang, Li Liu, Li Tian, Shengcheng Zhang, Qian Wang, Dejin Xie, Xuequn Chen, Zhenyang Liao, Yibin Wang, Jian Qin, Wanxia Yang, Caowen Sun, Xiangxiang Fu, Xingtan Zhang, Shengzuo Fang

**Affiliations:** 1 Nanjing Forestry University, Co-Innovation Center for Sustainable Forestry in Southern China, Nanjing 210037, China; 2 Shenzhen Branch, Guangdong Laboratory for Lingnan Modern Agriculture, Genome Analysis Laboratory of the Ministry of Agriculture, Agricultural Genomics Institute at Shenzhen, Chinese Academy of Agricultural Sciences, Shenzhen 518120, China; 3 Center for Genomics and Biotechnology, Fujian Provincial Key Laboratory of Haixia Applied Plant Systems Biology, Key Laboratory of Genetics, Breeding and Multiple Utilization of Crops, Ministry of Education, Fujian Agriculture and Forestry University, Fuzhou 350002, China

**Keywords:** *Cyclocarya paliurus*, Genomics, Whole-genome duplication, Triterpenoid, Resequencing

## Abstract

***Cyclocarya paliurus*** is a relict plant species that survived the last glacial period and shows a population expansion recently. Its leaves have been traditionally used to treat obesity and diabetes with the well-known active ingredient cyclocaric acid B. Here, we presented three *C*. *paliurus* genomes from two diploids with different flower morphs and one haplotype-resolved tetraploid assembly. Comparative genomic analysis revealed two rounds of recent **whole-genome duplication** events and identified 691 genes with dosage effects that likely contribute to adaptive evolution through enhanced photosynthesis and increased accumulation of **triterpenoids**. **Resequencing** analysis of 45 *C*. *paliurus* individuals uncovered two bottlenecks, consistent with the known events of environmental changes, and many selectively swept genes involved in critical biological functions, including plant defense and secondary metabolite biosynthesis. We also proposed the biosynthesis pathway of cyclocaric acid B based on multi-omics data and identified key genes, in particular gibberellin-related genes, associated with the heterodichogamy in *C*. *paliurus* species. Our study sheds light on evolutionary history of *C*. *paliurus* and provides **genomic** resources to study the medicinal herbs.

## Introduction

The “relict” plants consist of the remaining population of species that were widely distributed previously but are restricted to limited geographic regions currently. The severe population bottleneck is likely caused by large-scale environmental changes (such as global dramatic temperature decline) that have a fundamental impact on the ecosystem of the previously abundant species [1]. Evidence reveals that the population dynamics are recorded in the population genomes [2]. Recently developed sequencing technologies provide a practical approach to uncover the disaster during long-term evolutionary history [3], [4]. However, how relict plants survived from previous environmental changes and flourish in the new territory remains unclear.

*Cyclocarya paliurus* (Batal.) Iljinskaja (wheel wingnut), the sole species in the genus *Cyclocarya* Iljinskaja (Juglandaceae), is not only a well-known multi-function tree species [5], but also has the character of heterodichogamy, a transitional form in the evolution of plants from monoecism to dioecism [6]. Previous studies have indicated that its leaves are often used in traditional Chinese medicine to treat hypertension and diabetes due to its high biological activity and favorable safety [7]. Furthermore, the antihyperglycemic tea of *C*. *paliurus* was the first health tea approved by Food and Drug Administration (FDA) in 1999 [8]. These lines of evidence show that *C*. *paliurus* leaves contain multiple bioactive compounds, including triterpenoids, flavonoids, phenolic acids, steroids, and polysaccharides. These compounds protect humans against chronic diseases owing to their antidiabetic, antioxidant, and antimicrobic properties [6].

Triterpenoids are synthesized from six C5 (isopentenyl diphosphate) units from the common precursor 2,3-oxidosqualene by oxidosqualene cyclases (OSCs) [9]. These carbon skeletons are further oxidized by cytochrome P450 monooxygenases (P450s) and glycosylated by UDP-dependent glycosyltransferases (UGTs), resulting in diverse triterpenoid structures [10], [11]. Triterpenoids constitute a vast family of natural products that play an important role in significant biological and pharmacological effects, such as cyclocaric acid B extracted from *C*. *paliurus* leaves [12]. Cyclocaric acid B has a pharmacological activity on diabetes, and it can enhance glucose uptake by involving AMP-activated protein kinase (AMPK) activation and improving insulin sensitivity in adipocytes [12]. However, the evolutionary history and functions of cyclocaric acid B-related genes remain unknown, although ≥ 40 different triterpenoid compounds have been isolated from *C*. *paliurus* species [13]. Therefore, elucidation of the biosynthetic pathways leading to the production of cyclocaric acid B is greatly needed for heterologous bioproduction and a high public health priority.

With the importance in pharmaceutical values, a considerable production of *C*. *paliurus* leaves is required for medical use. However, *C*. *paliurus* seedlings can only be propagated from seeds, but its seed quality is deficient with the seed vigor of 0%–10% due to its heterodichogamy [14]. Heterodichogamy in *C*. *paliurus* possesses two temporally complementary morphs, protandry (PA) or protogyny (PG), in monoecious population. The stigma matures before pollen dispersal in PG, whereas pollen scatters before stigma maturation in PA. Hence, the female and male function segregation within PA or PG significantly affects seed filling index and quality. Although heterodichogamy is especially common in Fagales, Magnoliales, Malvales, Laurales, Sapindales, Canellales, Ranunculales, Zingiberales, Trochodendrales, Rosales, Caryophyllales, Malpighiales, and Apiales [15], the related genetic mechanism is far from well-studied.

The species *C*. *paliurus* is circumscribed with approximately two ploidy levels, including diploid (2*n* = 2*x* = 32) and auto-tetraploid (2*n* = 4*x* = 64), and it is mainly distributed across subtropical mountainous areas in China. Polyploidy or ancient whole-genome duplication (WGD) is a major driver of plant evolution [16] that contributes to variation in genome size and abundant genetic materials, as well as phenotypic and functional diversification of plants [17]. Although one polyploid *C*. *paliurus* genome has been reported recently, the collapsed assembly missed haplotypic variations that may underlie important functions [18]. In addition, the exact roles that WGD played in the origin and evolution of *C*. *paliurus* have not been clearly elucidated. Herein, we present three chromosome-scale genomes, containing two diploid *C*. *paliurus* individuals that represent two different flower morphs, protandrous (PA-dip) and protogynous (PG-dip), and one haplotype-resolved genome for auto-tetraploid. Our work uncovers the genetic mechanisms behind the special features of *C*. *paliurus* species, including heterodichogamy, origination, polyploidization, and cyclocaric acid B biosynthesis.

## Results

### Assembly and annotation of three *C*. *paliurus* genomes

**Figure 1:**
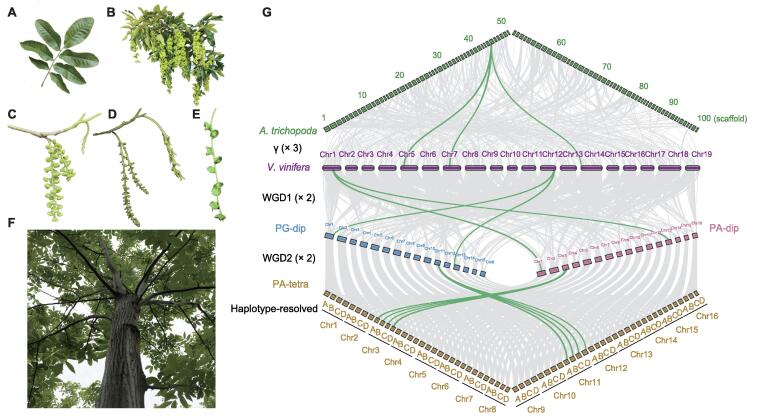
**Morphology and genome duplications of *C*.*paliurus*A.** Imparipinnate leaves. **B.** Mature fruits. **C.** Female and male flowers of PA-dip. **D.** Female and male flowers of PG-dip. **E.** Fruit wings that start to spread. **F.** Tetraploid plant. **G.** Genomic alignments between the basal angiosperm *A. trichopoda* and the basal eudicot *V*. *vinifera*, as well as PG-dip, PA-dip, and PA-tetra *C*. *paliurus* are shown. The conserved collinear blocks are shown as the gray lines in the background, and the green lines indicate cases in each round of WGD. *C. paliurus*, *Cyclocarya paliurus*; *A. trichopoda*, *Amborella trichopoda*; *V. vinifera*, *Vitis vinifera*; WGD, whole-genome duplication; PA-tetra, the protandrous tetraploid *C. paliurus*; Chr, chromosome; PG-dip, the protogynous diploid *C. paliurus*; PA-dip, the protandrous diploid *C. paliurus*.

We sequenced and assembled three *C*. *paliurus* genomes, including two diploid and one tetraploid individuals (Figure 1; Table 1, Tables S1–S4). The two diploid *C*. *paliurus* individuals represent two different reproduction types in hermaphroditism, PA-dip and PG-dip, whereas the tetraploid form is protandrous (PA-tetra). Karyotype analysis identified 32 chromosomes in the diploid genomes and 64 in the tetraploid genome (Figure S1). Taking the male florals of PA-tetra *C*. *paliurus* as experimental materials, the homologous chromosome synapsis at the early stage of meiosis in pollen mother cells of PA-tetra *C*. *paliurus* was studied. Multivalent (quadrivalent) phenomena were observed in pollen mother cells (Figure S2), strongly indicating that the PA-tetra is an auto-tetraploid. Using diploid *Pterocarya stenoptera* (600 Mb) as an internal reference, we estimated the genome size of PA-dip, PG-dip, and PA-tetra *C*. *paliurus* by flow cytometry (FCM) to be about 606 Mb (1C, the total number of DNA base pairs in one copy of the haploid genome), 659 Mb (1C), and 2460 Mb (1C = 1230 Mb), respectively (Figure S3). A total of 134.9 Gb, 75.5 Gb, and 271.8 Gb subreads were generated on the PacBio Sequel II platform, comprising ∼ 223×, ∼ 115×, and ∼ 221× coverages of the estimated genome sizes (by FCM, 1C) for PA-dip, PG-dip, and PA-tetra, respectively (Table 1, Table S1). The initial contigs were assembled using Canu assembler, resulting in three contig-level assemblies with N50 sizes of ∼ 1.9 Mb in PA-dip, ∼ 1.4 Mb in PG-dip, and ∼ 431 kb in PA-tetra (Table 1, Table S2). The assembled genome sizes were 586.62 Mb for PA-dip and 583.45 Mb for PG-dip, accounting for 96.8% and 88.5% of estimated genome size by FCM (Figure S3), respectively, while 2.38-Gb sequences were assembled in PA-tetra, almost four times of the haploid size (Table 1; Table S2). The chromosome-level assemblies were achieved using high-throughput chromatin conformation capture (Hi-C) technology (Table S3). For the diploid PA and PG genomes, 543.53 Mb (92.65%) and 553.87 Mb (94.93%) of sequences were integrated into 16 pseudo-chromosomes, respectively (Figure 1G; Table 1, Table S4). The PA-tetra genome comprised 64 pseudo-chromosomes with four sets of monoploid chromosomes using ALLHiC phasing algorithm that anchored 2.17 Gb (91.08%) of genomic sequences, representing a haplotype-resolved assembly of the tetraploid species (Figure 1G, Figure S4; Table 1, Table S4).

**Table 1:** **Summary on genome assembly and annotation of *C.paliurus***

**Sequencing**		**PA-dip**	**PG-dip**	**PA-tetra**
Illumina paired-end sequencing	Raw data (Gb)	106.7	86	291.7
	Coverage (×)	176	131	237
	Sequencing depth (×)	178	143	243
PacBio Sequel II sequencing	Raw data (Gb)	134.9	75.5	271.8
	Coverage (×)	223	115	221
	Sequencing depth (**×**)	225	125	226
Hi-C sequencing	Raw data (Gb)	65.4	68	264
	Coverage (×)	108	103	215
	Sequencing depth (**×**)	109	113	220
Contig-level assembly and annotation	Total length of contigs (Mb)	586.62	583.45	2380.95
	Contig N50 (bp)	1,928,354	1,389,753	430,910
Monoploid/haplotype-resolved chromosome-level genome assembly	Total length of chromosome-level assembly (Mb)	543.53	553.87	2168.65
BUSCO completeness of assembly (%)		95.2	96.4	95.5
Total number of protein-coding genes anchored on the chromosomes		34,699	35,221	34,633
BUSCO completeness of annotation (%)		96.2	96.2	94.4
Number of genes with 4 alleles		−	−	9362
Number of genes with 3 alleles		−	−	10,262
Number of genes with 2 alleles		−	−	7509
Number of genes with 1 alleles		−	−	7500
Number of unanchored genes/alleles		−	−	588

*Note*: Monoploid genome assembly and annotation were performed for PA-dip and PG-dip; haplotype-resolved chromosome-level genome assembly and annotation were performed fo PA-tetra. Only one allele was retained if the allelic genes had the exact same coding sequences. PA-tetra, the protandrous tetraploid *C*. *paliurus*; PG-dip, the protogynous diploid *C*. *paliurus*; PA-dip, the protandrous diploid *C*. *paliurus*; Hi-C, high-throughput chromatin conformation capture; BUSCO, Benchmarking Universal Single-Copy Orthologs.

We identified 12,737,788 haplotypic single nucleotide polymorphisms (SNPs) among the haplotype-resolved A/B/C/D homologous chromosomes, affecting 5152 functional genes (Table S5). These genes were significantly enriched in some primary biological pathways, such as aminoacyl-tRNA and fatty acid biosyntheses, glycerolipid metabolism, mitochondrial genome maintenance, and single strand break repair (Figure S5). We further assessed the quality of genome assemblies, showing more than 95.2% of Benchmarking Universal Single-Copy Orthologs (BUSCO) completeness (Table S6). Comparison with the previously published *C*. *paliurus* genome shows an improved BUSCO completeness (95.2% *vs.* 91%) with well-resolved duplicated genes (*i.e.*, allelic genes; BUSCO duplication 82.3% *vs.* 8.4% in Table S6) [18]. Our assessment using Illumina short reads showed at least 98.89% of global mapping ratio and 93.72% of properly paired reads (Table S7). Comparison among the three genomes revealed high levels of syntenic relationship with a large number of genes (26,760) located in the syntenic regions (Figure 1G). The Hi-C contact heatmaps also confirmed the high consistency of genome structure and quality, which also indicates improved chromosome-scale assemblies in comparison with the previously published genome [18] (Figures S6–S8).

We annotated 34,699 protein-coding genes in PA-dip and 35,221 protein-coding genes in PG-dip. The initial annotation of the tetraploid genome resulted in 90,752 gene models; however, this number mixed the concept of genes and allelic genes. To identify allelic genes that have at least one base substitution, we adopted the same strategy in our previously published sugarcane genome [19], leading to 34,633 allele-defined protein-coding genes in the tetraploid genome (Tables S8 and S9). Our assessment of the annotation showed 96.2%, 96.2%, and 94.4% of completeness for PA-dip, PG-dip, and PA-tetra, respectively, according to the 1375 conserved genes in BUSCO assessed using embryophyta_odb10 database (Table S10).

A total of 282.25 Mb (48.1% of the assembled genome), 316.95 Mb (54.3%), and 1,154.35 Mb (48.4%) repetitive sequences were identified in the PA-dip, PG-dip, and PA-tetra genomes, respectively, showing a slight increase in PG-dip genome (Table S11). The ratio of repetitive elements in the previously reported assembly by Zheng et al. [18] (14.94%) is much lower than our results, possibly due to a large proportion of collapsed sequences. Retroelements account for approximately three-quarters of the repetitive sequences, ranging from 35.7% to 37.3% in the three genomes. However, in contrast to other published plant genomes, such as pineapple [20], sugarcane [19], and banyan tree [21], long interspersed nuclear element (LINE) is the most prominent family in *C*. *paliurus*, spanning from 12.16% to 12.59% of the assembled genomes. In comparison, *Copia* and *Gypsy* account for only ∼ 5.47% and ∼ 5.94% of the assembled genomes on average in *C*. *paliurus* genomes (Table S11).

### An additional WGD event in the auto-tetraploid genome

**Figure 2:**
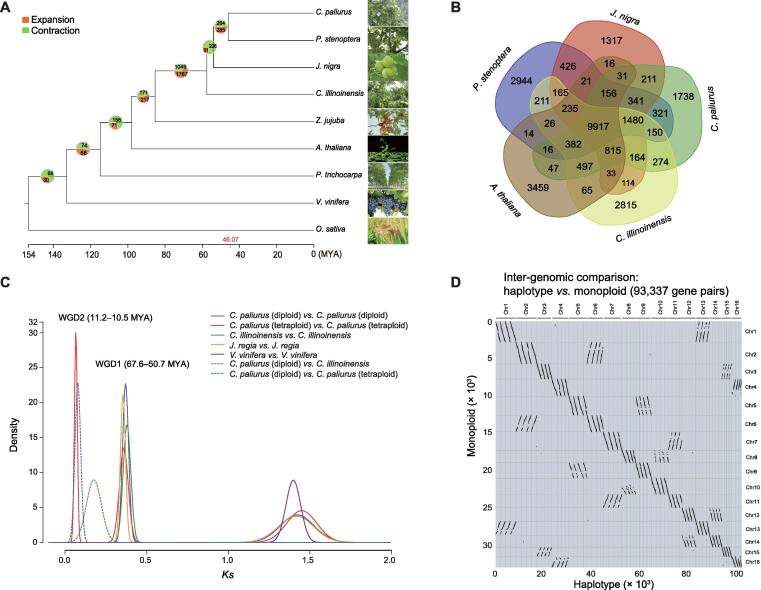
**Phylogenetic and comparative analyses of *C. paliurus*** **A.** Phylogenetic relationship of *C*. *paliurus*, *C*. *illinoinensis*, *J*. *nigra*, *P*. *stenoptera*, *A*. *thaliana*, *Z*. *jujuba*, *V*. *vinifera*, *P*. *trichocarpa*, and *O*. *sativa*. The divergence time among different plant species is labeled at the bottom. **B.** Venn diagram of orthologous and species-specific gene families in different plant genomes. **C.** Evolutionary analysis of the diploid and tetraploid *C*. *paliurus* genomes with the distribution of *Ks* values of orthologs*.***D.** Synteny analysis between PA-tetra and PA-dip genomes. “monoploid” indicates a reference genome assembly with only one representative haplotype retained, whereas “haplotype” indicates fully phased genome with all the four haplotypes. *C. illinoinensis*, *Carya illinoinensis*; *J. nigra*, *Juglans nigra*; *P. stenoptera*, *Pterocarya stenoptera*; *A. thaliana*, *Arabidopsis thaliana*; *Z. jujuba*, *Ziziphus jujuba*; *P. trichocarpa*, *Populus trichocarpa*; *O. sativa*, *Oryza sativa*; *Ks*, synonymous substitution rate; MYA, million years ago.

Maximum likelihood tree using 302 single-copy gene families from nine plant species reconstructed the phylogenetic relationship among *C*. *paliurus* and related species. The estimated divergence time between *C*. *paliurus* and *P*. *stenoptera* was approximately 46.07 million years ago (MYA) (Figure 2A), consistent with the previously reported divergence time of different genera of Juglandaceae [22]. Analysis of the gene families showed that a large number of families experienced expansion (285) and contraction (264) compared with *P. stenoptera* (Figure 2A)*.* In addition, we found 1738 gene families specific to the *C*. *paliurus* genome, while 9917 gene families were shared in the selected species, demonstrating evolutionary conservation **(**Figure 2B**)**.

The distribution of synonymous substitution rate (*Ks*) of each homologous gene pair within *C*. *paliurus* showed three peaks (Figure 2C), representing three WGD events. In addition to the ancient whole-genome triplication (WGT) event shared with grape, *C*. *paliurus* experienced two recent WGDs. Synteny analysis between PA-dip and PG-dip validated the early WGD (*i.e.*, WGD1), dating back to ∼ 67.6–50.7 MYA (Figure 2C, Figure S9). Most of the duplicated chromosomes maintained high levels of syntenic relationship and completeness compared with their counterparts. However, several structural variations were observed (Figure S9), *e.g.*, an inversion between chromosome 4 (Chr4) and Chr16 and a large deletion in Chr8 compared with Chr10, indicating the diploidization process in the diploid *C*. *paliurus* after the early WGD. The most recent WGD event (*i.e.*, WGD2) happened in ∼ 11.2–10.5 MYA and contributed to the tetraploidy in *C*. *paliurus* species (Figure 2C and D). To check whether each of two WGDs made specific contribution to specific gene family expansion in *C*. *paliurus*, we counted 1351 and 2024 genes, which experienced WGD1 and WGD2 events, respectively. Functional enrichment analysis showed that the genes involved in WGD1 event were significantly enriched in ribosomal subunit assembly, mannosyltransferase activity, and *N*-glycan biosynthesis, and the genes involved in WGD2 event were mostly enriched in terpene biosynthesis, such as sesquiterpenoid, triterpenoid, and monoterpenoid biosyntheses (Figure S10). Meanwhile, the genomic dot plots between *C*. *paliurus* and *Vitis vinifera* validated that tetraploid *C*. *paliurus* experienced two WGD events during the polyploidy evolution (Figures S11 and S12). We analyzed the timing of long terminal repeat retrotransposon (LTR-RT) insertions and found that LTR bursts occurred at ∼ 1.5 MYA across all the three genomes (Figure S13) after the divergence between tetraploid and diploid genomes (∼ 11.2–10.5 MYA). Collectively, the large tetraploid genome size is attributed to the most recent WGD (WGD2), rather that the expansion of repeats with the LTR bursts. The fully phased haplotypes facilitate us to investigate the evolution of polyploidy in *C*. *paliurus*. According to the method described by Mitros et al. [23] that depended on clustering of chromosome-specific *K*-mers (*K* = 13), we found that four haplotypes within each homologous group were consistently partitioned into a same branch (Figure S14), indicating a similar evolutionary history of the four haplotypes. In addition, the smudge pot analysis using heterozygous *K-*mer pairs extracted from Illumina sequencing reads suggests a highly heterozygous tetraploidy evidenced by the dominant component of AAAB pattern, accounting for 57% of tested *K*-mer pairs (Figure S4D). These lines of evidence collectively suggest that PA-tetra is likely an auto-tetraploid species with a high heterozygosity of 1.97% (Figure S4C).

### Expansion of P450 gene families is associated with elevated triterpenoid biosynthesis

Evidence has shown that triterpenoids, sterols, flavones, and phenol acids are enriched in *C*. *paliurus* leaves [24], which is possibly associated with the expansion of specific gene families related to the biosynthesis of these secondary metabolites. Gene Ontology (GO) and Kyoto Encyclopedia of Genes and Genomes (KEGG) functional enrichment analyses showed that many of the expanded genes (Figure 2A) were enriched in sesquiterpene, terpene, monoterpenoid, and triterpenoid biosynthesis pathways (Figures S15 and S16). For instance, we found that 15% (42/280 in total) of P450 genes expanded, among which 23 genes were clustered on Chr1, Chr4, and Chr12 (Figure S17). Phylogenetic analysis further identified that, among the 42 expanded P450 genes, 16 genes belong to the CYP89 family that participates in biosynthesis of tetracyclic triterpenoids [25], 18 genes belong to the CYP706 family that possibly contributes to the increased flavonoids [26], and six genes belong to the CYP82 family (Figure S18; Table S12).

### Genes associated with the heterodichogamy in *C*. *paliurus*

The most typical feature of *C*. *paliurus* is heterodichogamy with female and male functionally separated within PA or PG individuals, promoting outbreeding in an independent population. To investigate the genes triggering heterodichogamy in *C*. *paliurus*, we performed a comparative transcriptome analysis for the floral buds of PG and PA samples, respectively. These samples contained two tissue types (female and male floral buds) at five different developmental stages, namely from S0 to S4 [27].

Pairwise comparison between PG and PA female samples (PG-F *vs.* PA-F) identified 958 differentially expressed genes (DEGs) that were consistently up-regulated or down-regulated in at least two stages. Similarly, 2373 DEGs were found between PA and PG male samples (PA-M *vs.* PG-M) (Figure S19A and B). Functional analysis revealed that these DEGs were enriched in a series of biological processes involving in floral organ formation and development (Figures S19C and D, S20, and S21). Notably, many of up-regulated DEGs (128/855 in PG-F and 58/1539 in PA-M) were related to hormone biosynthesis and signaling pathways, indicating that hormones may contribute to the heterodichogamous morphs with asynchronous flowering. We further tested endogenous hormone contents in PA and PG floral buds, including gibberellin (GA_3_)_,_ auxin (IAA), and abscisic acid (ABA). The results displayed similar levels of IAA and ABA at each of the five stages especially at S0, an initial differential stage of floral buds (Figure S22). However, significantly increased levels of GA_3_ in PG samples were detected at S0 stage compared with PA (Figure S22), indicating that GA_3_ content likely plays a crucial role in regulating floral bud physiological differentiation and is responsible for the asynchronous flowering.

To investigate the co-expression networks during floral bud development, we identified co-expressed gene sets via weighted gene co-expression network analysis (WGCNA) package based on the 22 RNA sequencing (RNA-seq) datasets (Figure S23). After filtering genes with low expression, a total of 20,829 genes were retained, distributing in 26 modules (Figures S23 and S24). We observed that genes in three modules (darkorange: 47 genes; pink: 912 genes; red: 1244 genes) showed a high correlation (R^2^ ≥ 0.56, *P* ≤ 0.007, Pearson test) with GA_3_ content (Figures S25 and S26). In addition to “regulation of hormone and gibberellin biosynthetic process”, these genes were also functionally enriched in signal transduction, response to stimulus and stress, and biological regulation (Figures S27 and S28).

Key hub genes including transcription factor (TF)-coding genes were identified in the WGCNA analysis. For instance, *Trihelix-1* (CpaF1st06806), involved in endogenous hormone signaling and flower development [28], and *ERF066* (CpaF1st15865), responsible for embryo development and stress signal transduction [29], have the most edges (342 and 279, respectively) (Figure S25E) in the pink module. In red module, the top two most frequently connected hub genes, *ERF090* (CpaF1st01445) and *WRKY55* (CpaF1st00113) (Figure S25F), were identified that may play important roles in regulating the development of floral organs [30] and phytohormone-mediated signal transduction process [31].

### Dosage effect contributes to enhanced photosynthesis and increased accumulation of terpenoids

**Figure 3:**
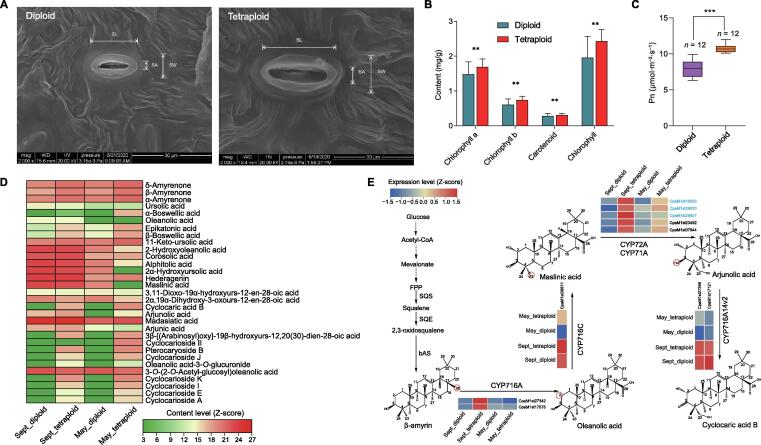
**Dosageeffect contributes to increased growth adaptability and accumulation of terpenoids** **A.** Scanning electron microscopy of stomata in diploid and tetraploid *C*. *paliurus* leaves. **B.** Comparison of chlorophyll content between diploid and tetraploid *C*. *paliurus* individuals. Statistical significance (*n* = 5) was determined using two-sided Student’s *t*-test. Error bars indicate mean ± SD of indicated replicates. **, *P* < 0.01. **C.** Comparison of Pn between diploid and tetraploid *C*. *paliurus* individuals. ***, *P* < 0.001. **D.** Heatmap showing the accumulation patterns of triterpenoids among the four samples. Sept_diploid indicates diploid samples collected in September; Sept_tetraploid indicates tetraploid samples collected in September; May_diploid indicates diploid samples collected in May; and May_tetraploid indicates tetraploid samples collected in May. **E.** Expression profiles of genes associated with cyclocaric acid B synthesis in *C*. *paliurus* tender and mature leaves for different ploidies. The scale ranging from blue (low) to red (high) indicates the expression magnitude of the FPKM values. The genes of CYP72A subfamily are shown in blue. The functions of the CYP716A14v2 and CYP716C subfamilies were identified by Miettinen and colleagues [11] and Moses and colleagues [32], [11]. SL, stomatal length; SA, stomatal aperture; SW, stomatal width; mag, magnification; WD, working distance; HV, high voltage; AM, ante meridiem; PM, post meridiem; SD, standard deviation; Pn, net photosynthetic rate; FPP, farnesyl pyrophosphate; SQS, squalene synthase; SQE, squalene epoxidase; bAS, β-amyrin synthesis; FPKM, fragments per kilobase per million.

Investigation of the morphology, anatomical structure, and photosynthetic capacity of the leaves of seedlings showed enhanced photosynthesis and accelerated plant growth in the tetraploid *C*. *paliurus*. We observed that a series of growth indices in the tetraploid plants were significantly higher than that in diploid individuals (*P* < 0.01, Duncan’s test), including seedling size, leaf area, length of compound leaves (Figure S29; Table S13), thickness of leaf tissues (upper and lower epidermal cells, palisade mesophyll, sponge tissue, and blade), stomatal size, stomatal density (Figure 3A, Figures S30 and S31), chlorophyll content (Figure 3B), and net photosynthetic rate (Pn; Figure 3C). In addition, we also detected three growth indices including blade aspect ratio, leaf moisture content, and leaf specific weight, which were significantly lower in the tetraploid individuals than those in diploid ones (Table S13).

To investigate genes underlying the functional impact of polyploidization, we identified 691 genes that showed significantly elevated expression in the tetraploid samples compared to diploid ones (fold change ≥ 2 and *P* ≤ 0.05) (Figure S32), which were considered as dosage-effect genes. Furthermore, functional annotation showed that these genes were abundantly enriched in some primary biological pathways (Figure S33). Notably, genes, which are involved in carbohydrate, starch, sucrose, alanine, aspartate, and glutamate metabolism, phosphatidylinositol signaling system, and ion channels, play vital roles in photosynthesis, and are indispensable for plant growth, development, and stress responses [33], [34]. We also explicitly investigated the *Arabidopsis thaliana* key homologous proteins in the photosynthetic pathway. Carbonic anhydrase (CA), phosphoenolpyruvate carboxylase kinase (PPCK), ribulose-1,5-bisphosphate carboxylase/oxygenase (Rubisco), fructose-1,6-bisphosphatase (FBP), and sedoheptulose-1,7-bisphosphatase (SBPASE) had significantly higher expression levels in tetraploid samples than those in diploid ones (*P* ≤ 0.05) (Figure S34). Meanwhile, we identified 759 dosage-effect genes with higher expression in diploid samples than in tetraploid ones (fold change ≥ 2 and *P* ≤ 0.05). GO and KEGG functional enrichment analyses showed that many of the dosage-effect genes were enriched in regulation of DNA recombination, maltose metabolic process, cysteine and methionine metabolism, and amino sugar and nucleotide sugar metabolism pathways (Figure S35).

Interestingly, we also noticed that many of the dosage-effect genes were significantly enriched in sesquiterpenoid and triterpenoid biosynthesis and terpenoid metabolism (*P* ≤ 0.05) (Figure S33), and likely contributed to the increased accumulation of some triterpenoid components in the PA-tetra (Figure 3D). Furthermore, we observed that 22 dosage-effect genes belong to the P450 gene family based on basic local alignment search tool (BLAST) results in public databases and phylogenetic analysis (Figures S36 and S37; File S1). Among them, three P450 subfamilies (CYP716A, CYP72A, and CYP71A) might be vitally important in the biosynthesis of cyclocaric acid B (Figure 3E) via modification of different C positions. Previous studies have reported that CYP716A12 and CYP716A1 can catalyze the oxidation at the C-28 position of β-amyrin, forming the triterpene oleanolic acid [35], [36], while the maslinic acid might be hydroxylated by CYP71A16 and CYP72A397 specifically at C-23 position into the triterpene arjunolic acid [37], [38]. Our study identified two homologous genes in the CYP716A subfamily, two in the CYP71A subfamily, and three in the CYP72A subfamily, showing dosage effects in the tetraploid *C*. *paliurus* (Figure 3E). In addition, they are likely the key genes contributing to the biosynthesis pathway of cyclocaric acid B (a specific triterpene to *C*. *paliurus*).

### Population genetics uncovers evolutionary history

**Figure 4:**
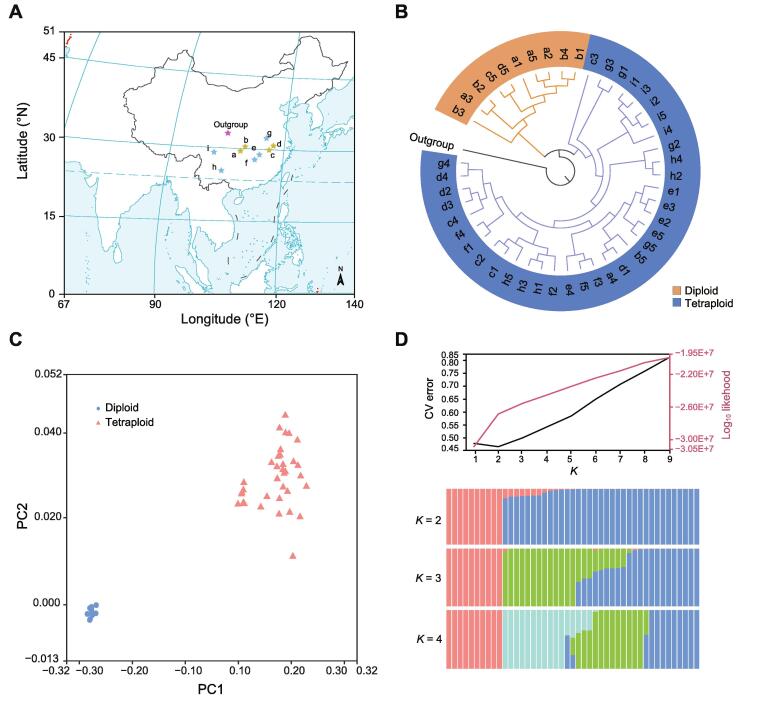
**Phylogenetic splits among *C*. *paliurus* populations** **A.** Dispersion of 45 individuals sampled from 9 sites (a–i) across most of the geographic range of *C*. *paliurus*. Populations are plotted with dots color-coded based on dispersion by latitude and longitude. Yellow stars represent the co-presence of diploid and auto-tetraploid distributions, blue stars represent only auto-tetraploid distribution, and purple star represents the outgroup (*Juglans regia*). More details are shown in Table S14. The world map was constructed from http://bzdt.ch.mnr.gov.cn/index.html. **B.** A phylogeny for *C*. *paliurus* individuals estimated from SNPs in neutrally evolving sites. **C.** PCA showing clear separation between diploid and auto-tetraploid populations. **D.** Top: CV plot displaying CV error *vs.* *K*. *K* = 2 is the best fit. Bottom: ADMIXTURE plot for *C*. *paliurus* showing the distribution of genetic clusters (*K* = 2, 3, and 4). *K* = 2 (representing the divergence within diploid and auto-tetraploid clades) indicates the smallest CV error. SNP, single nucleotide polymorphism; PCA, principal component analysis; CV, cross-validation.

To explore the population structure and evolutionary history of the *C*. *paliurus* populations, we resequenced 45 individuals, including 10 diploid and 35 tetraploid individuals native to the south of China, and one walnut species (*Juglans regia*) as an outgroup (Figure 4A; Table S14). Based on our stringent filtering criteria (see Materials and methods), we identified 3,886,832 variants [3,545,162 SNPs and 341,670 insertions/deletions (Indels)] from the diploid population, while identified 26,674,995 variants (23,076,276 SNPs and 3,598,719 Indels) from the tetraploid population. We also identified that the diploid and tetraploid populations contained 3845 and 38,899 Indels in genic regions, as well as 3753 and 23,764 synonymous variants and 5503 and 35,241 nonsynonymous variants, respectively (Table S15).

A phylogeny of these *C*. *paliurus* individuals collected from nine geographic locations partitioned these samples into two distinct groups (Figure 4B). The ten diploid samples were clustered in the first group, and most closely related to the outgroup *J*. *regia*. The remaining 35 tetraploid samples were represented in the second group. Both principal component analysis (PCA) and ADMIXTURE analysis supported the population structure (Figure 4C and D). These results indicated a single origin of the last WGD event in *C*. *paliurus* species rather than multiple origins observed in other polyploid species, such as sugarcane [19].

To identify the candidate genes responsible for coordinated local adaptation, we further analyzed selective sweeps based on the SweeD analysis in both diploid and auto-tetraploid genomes. A total of 25.88 Mb and 165.18 Mb genomic sequences were under purifying selection in diploid and tetraploid genomes, respectively (Figure S38). These selectively swept regions were evenly distributed in 16 chromosomes of PA-dip and 64 chromosomes of PA-tetra *C*. *paliurus*, with a number of them showing high levels of selective sweeps (Figure S38). These swept genomic regions overlapped with 1528 protein-coding genes in diploid, and 4812 allele-defined genes in the tetraploid group. Global GO and KEGG functional enrichment analyses also revealed that 114 and 568 of these swept genes respectively in diploid and tetraploid groups were involved in important functions, such as secondary metabolism, regulation of DNA recombination, and maltose metabolic process (Figures S39–S42).

Among these swept genes, 262 were shared between the diploid and tetraploid genomes, and also located in the syntenic regions. In addition, the selectively swept genes specific to tetraploid were mostly enriched in terpene synthase activity and terpene biosynthesis (Figures S43 and S44), probably caused by stronger environmental adaptation [39] and stress tolerance [40] than diploid. Further, three genes, CpaM1st41668, CpaM1st05982, and CpaM1st08040, were verified as orthologs of the *A*. *thaliana* genes *FAR1-related sequence* (*FRS10*), *Arabidopsis response regulator 1* (*ARR1*), and *Phytochrome C* (*PHYC*), respectively, which were associated with general environmental variables such as light regulation, temperature, and precipitation [41].

The reconstruction with the ancestral alleles from the diploid *C*. *paliurus* reference genome was used to estimate the site frequency spectrum (SFS) for 45 *C*. *paliurus* individuals using analysis of next generation sequencing data (ANGSD) and to generate a stairway plot elucidating effective population size (*Ne*) history over time. With an estimated mutation rate of 2 × 10^−9^ per generation and a generation time of 8 years, the stairway plot revealed two population bottlenecks over deep time (Figure S45). An early *Ne* bottleneck (dating back to ∼ 4.5–3.0 MYA) appeared during the upper Pliocene, consistent with the known events of environment change that boundary between the Pliocene and Miocene, was a regional transition from the warmer to the cooler stages [42]. The last *Ne* drop (dating back to ∼ 0.6–0.16 MYA) corresponded with the great extinction event at the Pleistocene glaciation [43], followed by a rapid population expansion. Facilitated by the comparison of the demographic bottlenecks in three *Camellia sinensis* genomes, we uncovered similar bottlenecks in *C*. *paliurus* (0.6–0.16 MYA) and *C*. *sinensis* (2.5–0.7 MYA), both coinciding with known periods of environmental change [44].

## Discussion

*C*. *paliurus* is well-known as the sweet tea tree and traditionally used as an herbal medicine [45]. The diverse ploidy and heterodichogamy in this species make it an ideal model to investigate the functional impact of WGDs and the development of flowers. We have generated three references, including two diploid and one haplotype-resolved tetraploid genomes, by incorporating the newly developed sequencing technologies and chromosome phasing algorithm. Facilitated by the comparison of these genomes and 45 resequenced individuals, we are able to investigate the evolutionary history and uncover functional genes underlying environmental adaptation as well as factors contributing to enhanced photosynthesis and the biosynthesis of cyclocaric acid B, one of the active components for the treatment of hypertension and diabetes [12].

Studies have shown that *Cyclocarya* is an ancient genus, likely originating in the late Paleocene and becoming extinct in the early Miocene except for *C*. *paliurus* from the subtropical China [13]. The remaining *C*. *paliurus* is a relict plant species within the genus, serving as an excellent model to study the adaptive evolution of relict plants. Our population analysis uncovered two bottlenecks in the species, with each coinciding with dramatic climate changes. However, the rapid demographic decline was recovered by population expansion as shown in the stairway plot, posing questions about how this species survived during the long-term evolutionary history. Increasing evidence has shown that WGD is a pivotal contributor to adaptation in angiosperms [46]. Two rounds of WGDs after the ancient WGT event shared by eudicots were observed in *C*. *paliurus*, occurring at ∼ 67.6–50.7 MYA and ∼ 11.2–10.5 MYA, respectively. Population genetics analysis clustered the 10 diploid and 35 tetraploid resequenced individuals into two distinct groups, indicating one single origination of the latest WGD event rather than multiple WGD events in a different location. Comparison between the auto-tetraploid and diploid genomes showed that the dosage effect after the most recent WGD involved genes contributing to adaptive evolution and improvement of photosynthesis, such as genes involved in the terpenoid metabolic biosynthetic pathways and the carbohydrate, starch, and sucrose metabolism. This is highlighted by the biosynthesis of cyclocaric acid B, which showed a significant increase in the auto-tetraploid genome compared with the diploid *C*. *paliurus*, attributing to elevated copy numbers of CYP716A, CYP72A, and CYP71A subfamily genes by WGDs. We also observed that the Pn, stomatal size, and chlorophyll content in the leaves of tetraploid *C*. *paliurus* were significantly higher than those of diploid, which is a benefit to improve its growth and environment adaptability. Moreover, combining the experimental phenomenon, the pollen viability of diploid *C*. *paliurus* is significantly lower than that in tetraploid (Figure S46). In conclusion, we propose that the tetraploid *C*. *paliurus* is more superior on the physiological and ecological characteristics than diploid.

As a typical heterodichogamy species, *C*. *paliurus* possesses two complementary morphs with asynchronous flowering, which may effectively prevent selfing, reduce intramorph inbreeding, and heavily contribute to the pattern of genetic diversity in the process of species evolution [47]. Our results uncovered that GA_3_ content plays an important role in the asynchronous flowering, which was also evidenced by up-regulated expression of GA-related genes in PG-F and PA-M. In addition to GA-related signaling pathway, co-expression network analysis identified that hub genes, including *Trihelix-1*, *ERF066*, *ERF090*, and *WRKY55*, likely contribute to heterodichogamy trait in *C*. *paliurus*.

## Materials and methods

### Homologous chromosome synapsis analysis of PA-tetra *C*. *paliurus*

The male florals at the early stage of meiosis of PA-tetra *C*. *paliurus* were transferred into Carnoy’s fluid (75% methanol and 25% glacial acetic acid) at 4 °C for 24 h under dark condition. Then 5 anthers were transferred to glass slide with 45% glacial acetic acid for 2 min acid hydrolysis. After covering the coverslip, the pollen mother cells were observed using phase contrast microscope, and the effective tablets were stored at −80 °C for 24 h. Then, 100% ethanol was added in materials at room temperature for dehydration of 30 min. Finally, 15 μl DAPI was added, and the tablet was examined by fluorescence microscope (Catalog No. Axioscope A1, Carl Zeiss, Jena, Germany).

### Sequencing and assembly of three *C*. *paliurus* genomes

The *C*. *paliurus* chromosome-level assemblies combined three technologies from single-molecule real-time (SMRT) sequencing with the PacBio Sequel technology, Hi-C, and short reads polished based on Illumina HiSeq sequencing. Briefly, raw data of ∼ 223×, ∼ 115×, and ∼ 221× coverages were generated on the PacBio Sequel II platform for PA-dip, PG-dip, and PA-tetra, respectively. Paired-end reads of ∼ 176×, ∼ 131×, and ∼ 237× coverages were generated on the Illumina NovaSeq 6000 platform for PA-dip, PG-dip, and PA-tetra, respectively (File S1).

The initial contig-level assemblies were accomplished using a series of PacBio assemblers. More specifically, the longest coverage of subreads from PacBio SMRT sequencing was self-corrected using Canu v.1.7 [48]. Then, error-corrected reads were assembled into genomic contigs using widely-used PacBio assembler Canu v.1.7 [48] with parameter corOutCoverage = 100. The N50 size, the assembled genome size (Table S2), and the complete BUSCO ratio (Table S6) were evaluated to inspect the quality of each round of assemblies. Last, the best results for subsequent analysis were selected through carefully manual inspection. Illumina paired-end reads were further used to polish the PacBio assemblies using Pilon v.1.18 [49]. Young leaves of *C*. *paliurus* were prepared for Hi-C library construction according to the standard protocol described previously [50]. The paired-end sequencing libraries were generated from chimeric fragments, followed by Illumina sequencing. The paired-end Hi-C reads were aligned to the contig-level assembly, and mis-joined contigs were then corrected using the 3D-DNA pipeline v.201008 [51] for abrupt long-range contact pattern detecting. The contigs corrected by Hi-C interactions were successfully linked into 16 pseudo-chromosomes in PG-dip and PA-dip, and 64 pseudo-chromosomes with four sets of haplotypes in PA-tetra *C*. *paliurus* using the ALLHiC pipeline [52], following the guideline that was used to assemble an auto-tetraploid sugarcane genome (https://github.com/tangerzhang/ALLHiC/wiki/ALLHiC:-scaffolding-an-auto-polyploid-sugarcane-genome).

### RNA extraction and sequencing

Total RNA was isolated from stems, leaves, leaf buds, and floral buds using E.Z.N.A Plant RNA Kit (Catalog No. R6827-01, Omega Bio-tek, Doraville, GA), and then purified with RNase-Free DNase I (Catalog No. 2270A, Takara Biotechnology, Dalian, China). Subsequently, 1% agarose gel was used to evaluate the RNA contamination and degradation. The purity was further monitored using ultraviolet spectrophotometer (Catalog No. NP80, Implen, München, Germany). Samples with RNA integrity number (RIN) values higher than 8 were used for downstream complementary DNA (cDNA) library preparation. The cDNA library construction was performed with the NEBNext Ultra RNA Library Prep Kit (Catalog No. E7770, New England BioLabs, Ipswich, MA) according to the manufacturer’s instruction. The Agilent 2100 Bioanalyzer system (Catalog No. G2938A, Agilent, Palo Alto, CA) was used for library quality assessing, and short paired-end reads were generated from the library preparation based on Illumina NovaSeq sequencing platform.

### Repeat annotation

Repetitive sequences were identified in the three *C*. *paliurus* genomes based on the same pipeline. First, RepeatModeler v.2.0.1 (https://www.repeatmasker.org/RepeatModeler/) and RepeatMasker v.4.0.5 (https://www.repeatmasker.org/) were used for *de novo* prediction of unkown transposable elements (TEs), as well as discovering known TEs. Next, TEclass v.2.1.3 [53] was further used to categorize the unknown TEs. Then, two pipelines Tandem Repeat Finder (TRF) v.4.07 [54] and LTR_Finder v.1.05 [55] were used to detect intact LTR-RTs and tandem repeats, respectively. Finally, LTRharvest v1.5.10 [56] and LTR_retriever [57] were used to construct a high-quality LTR library.

### Gene annotation

MAKER2 v.2.31.9 computational pipeline [58] was used to annotate genes in the three *C*. *paliurus* genomes, through a comprehensive strategy from RNA-seq-based prediction, homology-based prediction, and *ab initio* gene prediction. Briefly, RNA-seq data of different tissues of *C*. *paliurus* were assembled by Trinity v.2.6.5 software [59] with default parameters, genome-guided assembly, and *de novo* assembly. The fragments per kilobase per million (FPKM) expression values of assembled transcripts were quantified by RSEM [60], and the transcripts were removed if FPKM less than 1. The PASA v.r09162010 program [61] was applied to construct a comprehensive transcript library from the filtered transcripts. The almost “full-length” transcripts selected from PASA were aligned to the UniProt protein database, and protein sequences with coverage greater than 95% were reserved as candidate sequences. Afterward, the MAKER2 pipeline was used to integrate coding evidence of three annotation strategies (SNAP v.29–11-2013 [62], GeneMark v.4.28 [63], and AUGUSTUS v.3.2.3 [64]) and annotate protein-coding genes. After the first round, the predicated gene models with annotation edit distance (AED) values < 0.2 were selected for model re-training. Finally, gene annotation was improved from the second round of MAKER2. Further, the RNA-seq reads were aligned to reference genomes using HISAT2 v.2.0.4 with default parameters and re-assembled by StringTie v.2.2.0 [65]. The assembled RNA-seq transcripts and homologous proteins from *O*. *sativa*, *V*. *vinifera*, *Carica papaya*, *Morus notablis*, *Solanum tuberosum*, and *A*. *thaliana* were imported to MAKER2 pipeline. After filtering putative gene models of transposon-derived, a total of 34,699, 35,221, and 90,752 gene models were annotated in PA-dip, PG-dip and PA-tetra *C*. *paliurus* genomes, respectively. BUSCO analyses for PA-dip, PG-dip, and PA-tetra *C*. *paliurus* genomes were performed to evaluate completeness of the protein-coding annotations (Table S6).

### Phylogenetic tree reconstruction

To identify gene family groups, we analyzed protein-coding genes from 9 species, *C*. *paliurus*, *Carya illinoinensis*, *Juglans nigra*, *P. stenoptera*, *A*. *thaliana*, *Ziziphus jujuba*, *V*. *vinifera*, *Populus trichocarpa*, and *O*. *sativa* genomes. Gene family clustering was performed using OrthoFinder v.2.2.7 [66] based on 35,221 predicted genes of *C*. *paliurus*, and *O. sativa* was used as outgroup. Phylogenetic tree was constructed for *C*. *paliurus* and 8 other plant species based on coding sequence alignment of 302 single-copy gene families using FastTree v. 2.1.11 software [67]. The divergence time among 9 species was estimated by the r8s v.1.8.1 program [68]. For estimation of divergence time, we selected two calibration points from articles and calibrated the age of the nodes between *O. sativa* and *A*. *thaliana* (308–115 MYA), *J. nigra* and *P. stenoptera* (76–36 MYA), and *V. vinifera* and *A*. *thaliana* (135–107 MYA) according to the TimeTree website. The contraction and expansion of the gene families were observed by comparing the differences of cluster size between *C*. *paliurus* and each species using CAFE v.4.2.1 method [69].

### Analysis of genome collinear and WGD

For the comparative genomics analysis, the species we chose including *C*. *paliurus*, *P*. *stenoptera*, *J*. *nigra*, and *C*. *illinoinensis* all belong to the family Juglandaceae. The *Z*. *jujuba* shares the same typical feature of heterodichogamy with Juglandaceae. *A*. *thaliana* and *P*. *trichocarpa* were also chosen as the model plants for comparison. *V*. *vinifera* is a basal eudicot that has experienced a known WGT event, without a recent independent WGD. *O*. *sativa* as a monocotyledon, is used as an outgroup. Hence, the genomic information of these plant species are significant for comparative genomics analysis. We performed collinearity searches to identify collinear blocks within *C*. *paliurus* using MCScanX v.1.1.11 [70]. *Ks* between collinear genes were estimated based on whole-genome duplication integrated analysis (WGDI) v.0.1.6 software [71] with Yang-Nielsen (YN) model. In brief, the collinear blocks were constructed by performing similarity search for all-against-all protein sequence using BLASTP v.2.8.1 [72] (cutoff E-value of 1E−10), and then homologous block was built through MCScanX software. Finally, the *Ks* of each homologous gene pair was calculated.

### Analysis of the tetraploidy signatures

#### Identification of chromosome-enriched K-mers

To determine whether the genome is auto-tetraploid or allo-tetraploid, we adopted and modified a similar method that was used to study an allo-tetraploid *Miscanthus sinensis* genome [23] based on counting of chromosome-enriched 13-mers. Briefly, 13-mers were identified across the whole genome using Jellyfish v.2.2.6 [73], and only *K*-mers that met the following two conditions were retained: (1) *K*-mers occurring at least 1000 times globally; and (2) *K*-mers that were enriched in any chromosome with at least twofold difference. Applying of the filtering strategy resulted in a total of 11,783 chromosome-enriched 13-mers. We further clustered these selected *K*-mers based on the number of these 13-bp short sequences presenting across the whole genome. The results showed that every homologous chromosome group that comprises 4 haplotypes were grouped together, providing strong evidence of auto-tetraploidy signatures.

#### Smudge plot analysis

We also performed the smudge plot analysis [74] to investigate the polyploid signatures in this tetraploid species. This tool utilized heterozygous paired *K*-mers extracted from raw reads in the sequenced genome, and analyzed the genome structure by comparing the total coverage of paired *K*-mers (*i.e.*, coverage A + coverage B) to the relative coverage of the minor one, *i.e.*, coverage B / (coverage A + coverage B), where A and B are paired heterozygous *K*-mers, A indicates the dominant *K*-mer, and B indicates the minor one.

### Identification of DEGs between PG and PA

Male and female floral buds in various PA and PG individuals were collected at five different stages including (1) S0, physiological differentiation period; (2) S1, dormancy period; (3) S2, germination period; (4) S3, inflorescence elongation period; and (5) S4, maturation period. Three biological replicates of the extracted RNA from flowers (in PA and PG individuals) were sequenced on Illumina NovaSeq platform, and 6-Gb RNA-seq raw data for each sample were generated. Paired-end short reads were aligned to PG-dip *C*. *paliurus* genome using HISAT2 [75]. The expected number of FPKM fragments mapped were calculated using RSEM v.1.3.0 program [60], which was implanted in Trinity package [59]. Moreover, the DESeq2 R package v.1.30.0 [76] was applied to identify the DEGs. The clusterProfiler R package v.3.12.0 [77] was used for GO and KEGG enrichment analyses.

### Genome screening for P450 genes and gene cluster analysis

The hidden Markov model (HMM) profile for the P450 genes (PF00067) was obtained from the Pfam database (https://www.ebi.ac.uk/interpro/set/all/entry/pfam/). Then, the P450 genes were identified using the HMMER v3.3.2 [78] with default parameters by searching against the *C*. *paliurus* genome. According to definite standards with 40% for family variants described by Xiong [79], P450 genes were divided into 68 families by alignment with P450 database [80]. Maximum likelihood phylogenetic trees were constructed using the Randomized Axelerated Maximum Likelihood (RAxML) package v.8.2.11 [81] with full-length protein sequences.

The general feature format (GFF) files of 42 expanded P450 genes were used to search for gene clusters. The gene clusters were verified by following criteria: (1) one gene cluster should contain at least three adjacent P450 genes; and (2) the gene clusters were ruled out if the distance between adjacent P450s was more than 0.8 Mb.

### Identification of expressed genes and triterpenoid compounds for *C. paliurus*

Leaves in various PA-dip and PA-tetra individuals were collected in May and September, respectively. Three biological replicates of the extracted RNA were sequenced on Illumina NovaSeq platform, and 6-Gb RNA-seq raw data for each sample were generated. Paired-end short reads were aligned to PA-dip *C*. *paliurus* genome using HISAT2. Meanwhile, the same leaf samples were prepared for ultra performance liquid chromatography-tandem mass spectrometry (UPLC-MS/MS) analysis. UPLC-MS/MS system (UPLC, SHIMADZU Nexera X2; MS/MS, Applied Biosystems 4500 Q TRAP, Waters, Milford, MA) was used to analyze the differences in triterpenoid accumulation between diploid and tetraploid plant leaves. Sample extraction details are described in File S1. The UPLC operation parameters were as follows: chromatographic separation was carried out using an Agilent SB-C18 Analytical high-performance liquid chromatography (HPLC) Column (2.1 mm × 100 mm, 1.8 µm); the mobile phase consisted of ultrapure water (A) and acetonitrile (B), which both contained 0.1% formic acid. Gradient elution was as follows: original ratio of 5% B; B ratio linearly increased to 95% within 9 min and maintained for 1 min; B ratio decreased to 5% during 10–11.1 min and kept for 2.9 min. The flow rate was kept at 0.35 ml/min, temperature 40 °C, and injection volume 4 µl. The electrospray ionization (ESI)-triple quadrupole-linear ion trap (Q TRAP)-MS system used for MS experiment. The operating parameters of the ESI source were as follows: positive ion spray voltage (IS) 5500 V and negative ion mode − 4500 V; source temperature 550 °C; curtain gas (CUR) 25 psi, ion source gas I (GSI) 50 psi, and gas II (GSII) 60 psi, respectively. The 10 μmol/l and 100 μmol/l polypropylene glycol solutions were, respectively, implemented for mass calibration and instrument tuning in linear ion trap (LIT) modes and triple quadrupole (QQQ).

### Population genetic structure

More than coverage of 10× per sample for tetraploid and diploid *C*. *paliurus* were generated from 74 and 35 billion 150-bp Illumina short reads, respectively. Clean data were obtained by removing adapters and low-quality sequences (Q < 30) from paired-end raw reads, followed by aligning against the reference genome of PA-dip *C*. *paliurus* by BWA v.0.7.17 [82] with default parameters. The variant calling was carried out by GATK v.4.0.3.0 [83] following the best practice workflow. The general variants were identified for each individual using GATK HaplotypeCaller, and then combined by GenotypeGVCFs function to a single variant calling file. This two-step approach was carried out to ensure variant accuracy, which included re-genotyping and quality recalibration in the combined VCF file. SNPs were then identified using SAMtools/BCFtools with default parameters based on alignments of all Illumina short reads. SNPs were filtered by following parameters: (1) SNPs were only present in one of the two pipelines (SAMtools/BCFtools and GATK); (2) SNPs with read depth more than 1000 or less than 5; (3) non-biallelic SNPs; (4) SNPs with missing rate more than 40%; (5) SNPs in repeat regions; and (6) SNPs having less then 5-bp distance with nearby variant sites. A phylogenetic gene tree was constructed based on SNPs in the single-copy genes regions. Two popular programs (RAxML [84] with GTRCAT model and IQ-TREE [85] with self-estimated best substitution model) were applied to construct the maximum likelihood tree. Ancestral population structure among nine *C*. *paliurus* populations was estimated from ancestral population sizes *K* = 1–5 by ADMIXTURE software v.1.3.0, and the population size with the smallest cross-validation error (*K* = 2) was determined. ADMIXTURE analysis was performed following the parameter standard errors, which were estimated by bootstrapping (bootstrap = 200).

### Identification of selective sweeps

To identify selective sweeps, SweeD v.3.0 [86] program was used to identify regions that display significant variations in the SFS by the composite likelihood ratio (CLR) statistic. We allowed 0.3% with maximum missing data in per site, and top 5% as the threshold to screen candidate selective sweeps. Ten diploid and 35 tetraploid individuals were compared to PA-dip and PA-tetra reference genomes, respectively.

## Data availability

The whole-genome sequencing raw data including Illumina short reads, PacBio long reads, Hi-C interaction reads, and transcriptome data have been deposited in the Genome Sequence Archive [87] at the National Genomics Data Center (NGDC), Beijing Institute of Genomics (BIG), Chinese Academy of Sciences (CAS) / China National Center for Bioinformation (CNCB) (GSA: CRA004671), and are publicly accessible at https://ngdc.cncb.ac.cn/gsa/. The genome assemblies and annotations have been deposited in the Genome Warehouse [88] at the NGDC, BIG, CAS / CNCB (GWH: GWHBKKW00000000, GWHBKKX00000000, and GWHBKKY00000000), and are publicly accessible at https://ngdc.cncb.ac.cn/gwh/.

## Competing interests

The authors have declared no competing interests.

## CRediT authorship contribution statement

**Yinquan Qu:** Investigation, Methodology, Formal analysis, Visualization, Writing – original draft, Writing – review & editing. **Xulan Shang:** Resources, Methodology, Writing – original draft. **Ziyan Zeng:** Visualization, Methodology. **Yanhao Yu:** Resources, Formal analysis. **Guoliang Bian:** Resources, Data curation. **Wenling Wang:** Formal analysis. **Li Liu:** Visualization, Formal analysis. **Li Tian:** Formal analysis. **Shengcheng Zhang:** Formal analysis. **Qian Wang:** Resources. **Dejin Xie:** Formal analysis. **Xuequn Chen:** Formal analysis. **Zhenyang Liao:** Formal analysis. **Yibin Wang:** Formal analysis. **Jian Qin:** Formal analysis. **Wanxia Yang:** Resources. **Caowen Sun:** Resources. **Xiangxiang Fu:** Methodology, Resources, Writing – original draft, Writing – review & editing, Supervision. **Xingtan Zhang:** Methodology, Formal analysis, Writing – original draft, Writing – review & editing. **Shengzuo Fang:** Conceptualization, Methodology, Resources, Writing – original draft, Writing – review & editing, Supervision. All authors have read and approved the final manuscript.

## Supplementary material

Supplementary File 1 **Supplementary information and methods**

Supplementary Figure S1 **Chromosome karyotypes of *C. paliurus* A.** PA-dip. **B.** PG-dip. **C.** PA-tetra.

Supplementary Figure S2 **The phenomena of homologous chromosome****synapsis at the early stage of meiosis Ⅰ in PA-tetra *C. paliurus*** A total of 11 pollen mother cells were selected to observe and the red rounds represent the quadrivalent.

Supplementary Figure S3 **Using flow cytometry to estimate genome size of *C*. *paliurus* A.** PG-dip. **B.** PA-dip. **C.** PA-tetra. The *P*. *stenoptera* genome (2n = 2× = ∼ 600 Mb) and PA-tetra *C*. *paliurus* were used as an internal reference standard.

Supplementary Figure S4 ***K-mer* (21-mer) distribution and estimation of genome size and heterozygosity of *C. paliurus* A.** PG-dip. **B.** PA-dip. **C.** PA-tetra. **D.** Total coverage of the k-mer pair (A + B) in PA-tetra *C. paliurus*.

Supplementary Figure S5 **Functional enrichment analysis of selected genes involved in haplotypic variations in PA-tetra genome A.** GO. **B.** KEGG. GO, Gene Ontology; KEGG, Kyoto Encyclopedia of Genes and Genomes.

Supplementary Figure S6 **Genome-wide analysis of chromatin interactions at 150-kb resolution in PA-dip genome** The colored bar on the right represents the strength of interaction.

Supplementary Figure S7 **Genome-wide analysis of chromatin interactions at 150-kb resolution in PG-dip genome**

Supplementary Figure S8 **Genome-wide analysis of chromatin interactions at 150-kb resolution in PA-tetra genome**

Supplementary Figure S9 **Synteny analysis between PA-dip and PG-dip genomes**

Supplementary Figure S10 **Functional enrichment analysis of selected genes involved in WGD1 and WGD2 events A.** GO enrichment analysis of selected genes involved in WGD1 event. **B.** KEGG enrichment analysis of selected genes involved in WGD1 event. **C.** GO enrichment analysis of selected genes involved in WGD2 event. **D.** KEGG enrichment analysis of selected genes involved in WGD2 event.

Supplementary Figure S11 **Synteny analysis between PA-dip and*****V. vinifera*** **genomes**

Supplementary Figure S12 **Synteny analysis between PA-tetra and*****V. vinifera*** **genomes**

Supplementary Figure S13 **Comparison of timing of LTR-RT insertions among PA-tetra, PA-dip, and PG-dip genomes** LTR-RT, long terminal repeat retrotransposon.

Supplementary Figure S14 **Clustering of counts of*****K-mers*** **(*****K*** **= 13) enables the consistent partitioning of four haplotypes in each homologous chromosome into same group**

Supplementary Figure S15 **GO enrichment analysis of genes experienced expansion in*****C. paliurus***

Supplementary Figure S16 **KEGG pathway analysis of genes experienced expansion in*****C. paliurus***

Supplementary Figure S17 **The P450 clusters of expanded genes are arranged on Chr1, Chr4, and Chr12** P450, cytochrome P450 monooxygenase.

Supplementary Figure S18 **Phylogenic analysis of P450 families** The yellow and blue branches indicate the sequences from *Arabidopsis* and *C. paliurus*, respectively. The dots represent P450 genes. The outermost arc indicates the P450 gene family.

Supplementary Figure S19 **Identification and functional enrichment of DEGs between PG and PA *C. paliurus* at five flowering time stages (S0–S4) A**. Venn diagrams of DEGs in the female floral buds (PG-F vs. PA-F). **B.** Venn diagrams of DEGs in the male floral buds (PA-M vs. PG-M). **C.** GO enrichment of the 958 DEGs in female floral buds (PG-F vs. PA-F). **D.** GO enrichment of the 2373 DEGs in male floral buds (PA-M vs. PG-M). In all subsequent figures, PG-F means female floral buds of PG, PA-F means female floral buds of PA, PG-M means male floral buds of PG, and PA-M means male floral buds of PA. FC, fold change; DEGs, differentially expressed genes; PG, protogyny; PA, protandry.

Supplementary Figure S20 .**KEGG enrichment of the 958 DEGs in female floral buds (PG-F *vs.* PA-F) at five flowering time stages (S0–S4)**

Supplementary Figure S21 **KEGG enrichment of the 2373 DEGs in male floral buds (PA-M *vs.* PG-M) at five flowering time stages (S0–S4)**

Supplementary Figure S22 **Hormone contents of *C. paliurus* during five flower differentiation and development stages A.** Identification of GA3 level in female and male floral buds from PG or PA type. **B.** Identification of ABA level in female and male floral buds from PG or PA type. **C.** Identification of IAA level in female and male floral buds from PG or PA type. FW, fresh weight; GA3, gibberellin; ABA, abscisic acid; IAA, auxin.

Supplementary Figure S23 **Hierarchical clustering tree (dendrogram) of genes based on co-expression network analysis in PG and PA individuals (female flora buds, male flora buds, female leaves, and male leaves) during five development stages** Each individual’s value is the average of the expression of three replicate samples. PG-L means leaves of PG and PA-L means leaves of PA.

Supplementary Figure S24 **Co-expression modules identified by weighted gene co-expression network analysis (WGCNA)**

Supplementary Figure S25 **Functions and networks of co-expression module genes A.** Module–trait relationships. The column indicate GA_3_, and the rows indicate the different modules. The red and blue colors indicate positive and negative correlations, respectively. The correlation coefficient (*r*) and *P* value (*P*) are displayed in each cell. **B.** Eigengene expression profiles in the darkorange module. **C.** Eigengene expression profiles in the pink module. **D.** Eigengene expression profiles in the red module. **E.** Construction of the correlation network of the pink module. **F.** Construction of the correlation network of the red module. The large circles represent transcription factors. The color is determined by the edge number of the gene.

Supplementary Figure S26 **Expression profiles of three modules and GA3 contents during five stages**

Supplementary Figure S27 **GO enrichment of three modules (darkorange, red, and pink) genes**

Supplementary Figure S28 **KEGG enrichment of the three modules (darkorange, red, and pink) genes**

Supplementary Figure S29 **The morphological difference between diploid and tetraploid *C. paliurus* A.** Compound leaf. **B.** Singe leaf. **C.** Seedlings.

Supplementary Figure S30 **The comparison of leaf thickness and stomatal density between diploid and tetraploid *C. paliurus* based on scanning electron microscopy A.** Leaf thickness of diploid sample. **B.** Leaf thickness of tetraploid sample. **C.** Stomatal density of diploid sample. **D.** Stomatal density of tetraploid sample. U-ep, upper epidermal cells; L-ep, lower epidermal cells; Pal, palisade mesophyll; Sp, sponge tissue.

Supplementary Figure S31 **Quantification of anatomical structure values in different ploidy *C. paliurus* A.** Thickness of leaf tissues. **B.** Stomatal size of leaf. **C.** Stomatal density. **, *P* value < 0.01.

Supplementary Figure S32 **Heatmap showing the expression patterns of 691 dosage-effect genes among tetraploid and diploid samples**

Supplementary Figure S33 **KEGG pathway enrichment analysis of up-regulated genes in tetraploid samples**

Supplementary Figure S34 **Heatmap showing the expression patterns of photosynthesis**-**related genes** CA, carbonic anhydrase; PPCK, phosphoenolpyruvate carboxylase kinase; Rubisco, ribulose-1,5-bisphosphate carboxylase/oxygenase; FBP, fructose-1,6-bisphosphatase; SBPASE, sedoheptulose-1,7-bisphosphatase.

Supplementary Figure S35 **Functional enrichment analysis of dosage compensation effect genes in*****C. paliurus***

Supplementary Figure S36 **Heatmap showing the expression patterns of P450 family genes of dosage****effect genes across four samples**

Supplementary Figure S37 **Phylogenetic tree of P450 gene family in multiple species, including *C. paliurus*, *Centella asiatica*, *Avicennia marina*, *Lagerstroemia speciosa, Moreua rubra*, *J. regia*, *Prunus duscis, A. thaliana*, *Kalopanax truncatula*, *Barbarea vulgaris*, *Chenopodium quinoa*, *Lotus japonicus*, *Quercus suber*, *Kalopanax septemlobus*, *and Quercus lobata***

Supplementary Figure S38 **Distribution of selective sweep regions in *C. paliurus* genome A.** Diploid. **B.** Tetraploid. CLR, composite likelihood ratio.

Supplementary Figure S39 **GO enrichment analysis of genes under strong selective sweep in diploid*****C. paliuru***

Supplementary Figure S40 **KEGG pathway analysis of genes under strong selective sweep in diploid *C. paliurus***

Supplementary Figure S41 **GO enrichment analysis of genes under strong selective sweep in tetraploid*****C. paliurus***

Supplementary Figure S42 **KEGG pathway analysis of genes under strong selective sweep in tetraploid*****C. paliurus***

Supplementary Figure S43 **Venn diagrams of selective genes in diploid and tetraploid*****C. paliurus***

Supplementary Figure S44 **GO and KEGG enrichment of selective genes specific to tetraploid*****C. paliurus***

Supplementary Figure S45 **Demographic history of *C. paliurus*** Historical effective population size for *C. paliurus* beginning from 8 million years ago to present. Stairway plot showing that the *C. paliurus* population has undergone bottlenecks during two known periods of major climate upheaval: the Pleistocene (purple) and the Pliocene (blue). Ne, effective population size.

Supplementary Figure S46 **The comparison of pollen viability between diploid and tetraploid*****C. paliurus***

Supplementary Table S1 **Sequencing information for the assemblies**

Supplementary Table S2 **Contig-level assemblies**

Supplementary Table S3 **Statistics of Hi-C mapping**

Supplementary Table S4 **Chromosome-level genome assemblies**

Supplementary Table S5 **Characteristics of genetic variation compared with monoploid genome in PA-tetra*****C. paliurus***

Supplementary Table S6 **BUSCO assessment of genome assemblies**

Supplementary Table S7 **Statistics of Illumina short reads remapped to the assemblies**

Supplementary Table S8 **Allele annotation in auto-tetraploid*****C. paliurus*** **genome**

Supplementary Table S9 **Genes number of the three*****C. paliurus*** **genomes**

Supplementary Table S10 **BUSCO analysis of annotation completeness**

Supplementary Table S11 **Repeat annotation of the three*****C. paliurus*** **genomes**

Supplementary Table S12 **The expanded P450s families in the*****C. paliurus*** **genome**

Supplementary Table S13 **Comparison of growth indices between diploid and tetraploid*****C. paliurus*** **seedling, one year after stumping**

Supplementary Table S14 ***C. paliurus*** **and outgroup (*****Juglans regia*****) species used for the population genomics analysis**

Supplementary Table S15 Summary of genetic variations in *C. paliurus* populations
