# Supplementary material for: Whole-genome Duplication Reshaped Adaptive Evolution in A Relict Plant Species, Cyclocarya paliurus
Source: Genomics Proteomics Bioinformatics. 2023 Feb 11;21(3):455–69. doi: 10.1016/j.gpb.2023.02.001 (PMC10787019; doi:10.1016/j.gpb.2023.02.001)
Supplement: Supplementary File 1 — Supplementary information and methods [file mmc1.docx]

**File S1 Supplementary information and methods**

**Genome sequencing and assembly** **of three *Cyclocarya paliurus***

*Plant material*

Tissues of two diploid *C*. *paliurus* (PG-dip and PA-dip) and one autotetraploid (PA-tetra) for genome sequencing were collected from plants grown in germplasm bank of *C*. *paliurus*, which locates in Baima experimental, Nanjing, Jiangsu Province, China. Tissues were immediately frozen in liquid nitrogen and stored at −80 ℃.

*Illumina short reads sequencing*

Total DNA was isolated and extracted using the QIAGEN DNaesy Plant Mini Kit (Catalog No. 69104, Qiagen, Hilden, Germany) and sequenced in Illumina NovaSeq 6000 with 150-bp paired-end reads length.

*PacBio library construction and sequencing*

Approximately 20 μg of high-molecular-weight genomic DNA was sheared to an ~20 kb targeted size, followed by damage repair and end repair, blunt-end adaptor ligation, and size selection with a BluePippin system (Catalog No. BLU0001, Sage Science, Beverly, USA). The final libraries were subsequently sequenced using a PacBio Sequel II with P6-C4 chemistry. After data filtering and preprocessing, 134.9, 75.5, and 271.8 Gb raw data were generated for PA-dip, PG-dip, and PA-tetra *C*. *paliurus*, respectively (Table S1).

*High-throughput chromatin conformation capture (Hi-C) library construction and sequencing*

Hi-C libraries were created from tender leaves of *C*. *paliurus* plants as described before [1]. Briefly, the leaves were fixed with lysed formaldehyde, and then the cross-linked DNA was digested with HindIII over night. Sticky blunt ends were biotinylated and proximity-ligated to form chimeric junctions, and then physically sheared to a size of 500–700 bp. Chimeric fragments representing the original cross-linked long-distance physical interactions were then processed into paired-end sequencing libraries. A total of 196, 190, and 687 million of 150-bp paired-end reads were produced on Illumina NovaSeq 6000 system. Hi-C sequencing was assessed using HiC-Pro program and the results showed a decent proportion of validate reads (no less than 80% in three *C*. *paliurus* Hi-C libraries), suggesting high quality of the Hi-C data (Table S3).

**Estimation of** ***C*. *paliurus* genome size**

To estimate the genome size of the three *C*. *paliurus*, Illumina short reads were recruited to determine the distribution of *K*-mer values using published Perl scripts (https://bioinformatics.uconn.edu/genome-size-estimation-tutorial/). The genome size was estimated using the following formula: the number of *K*-mers divided by the depth of peaks (*K*-mer number/depth). The size of the haploid genomes for PA-dip and PG-dip *C*. *paliurus* were estimated to be approximately 538.16 Mb and 574.97 Mb, which are similar with the genome size of *Pterocarya stenoptera*. In addition, the estimated genome size is 2065.41 Mb for PA-tetra *C*. *paliurus* with high heterozygosity. To obtain estimation of nuclear DNA content, we followed previous method [2] to perform flow cytometry. The estimated genome sizes are consistent with our *K*-mer based estimation with an average 606 Mb for PA-dip, 659 Mb for PG-dip, and 2460 Mb for PA-tetra *C*. *paliurus* (Figure S4).

**Estimation of genome heterozygosity rate**

Ideally, each *K*-mer in a set of genomes should be a single occurrence, and there is no repetitive sequence or heterozygosity. In actual samples, due to the influence of heterozygous and repetitive sequences, the corresponding frequency of each *K*-mer is uncertain. We classified *K*-mer according to the frequency of appearance (i). Then, We calculated the percentage of each type of *K*-mer number: a_i_ = n_i,Kspecies_/n_Kspecies_, and the percentage of the number: bi = n_i,Kindividuals_/n_Kindividuals_. Considering the distribution of *K*-mer frequencies in the genome is [1,m], and the expected depth of each category is C_i_ = i*C, the probability function of the number of *K*-mer species in the genome with heterozygosity and repetitive sequences could be obtained (P_Kspecies_(x) represents the Poisson distribution with the expected depth Ci):

$$P_{K\text{species}}(x)=\sum_{i=1}^{m} a_{i}\times P_{Kspecies,i}(x)$$

The probability function of the number of *K*-mers in a genome with heterozygosity and repetitive sequences could be obtained (P_Kindividuals_(x) represents the deformed Poisson distribution with the expected depth Ci.):

$$P_{K\text{in}dividuals}(x)=\sum_{i=1}^{m} b_{i}\times P_{kindividuals,i}(x)$$

For heterozygous genomes, all *K*-mers can be divided into two categories: heterozygous *K*-mers and homozygous *K*-mers. There are 2*K heterozygous *K*-mers covered at each heterozygous site. Hence, the expected depth of the heterozygous *K*-mer is C/2 compared to the expected depth of the homozygous *K*-mer.

Using this method, the number of heterozygous sites and genome size could be estimated by a_1/2_ and n_Kspecies_, and the heterozygosity rate was estimated to be 1.97% (a_1/2_ is the percentage of the number of heterozygous *K*-mer species, n_Kspecies_ is the number of all *K*-mer types):

$$\varphi=\frac{\text{a}_{1/2}\times n_{Kspecies}/(2\times K)}{n_{Kspecies}-a_{1/2}\times n_{Kspecies}/2}=\frac{a_{1/2}}{K(2-a_{1/2})}$$

**Identification of triterpenoid compounds for *C*. *paliurus***

Standards (chromatographic purity) were purchased from Sigma-Aldrich (St Louis, MO). Methanol and acetonitrile were all purchased from Merck (Darmstadt, Germany). *C*. *paliurus* leaves of vacuum freeze drying were ground into powder (30 Hz, 1.5 min), and 100 mg powder were extracted with 1.2 ml methanol solution (70%). The extract was vortexed every 30 min and repeated in six sets of 30 s, and stored at −20 °C for one night. After centrifugation (rotating speed 12,000 rpm, 10 min), the supernatants were filtered with microporous membrane (0.22 µm pore size) and collected for ultra performance liquid chromatography-electrospray ionization-tandem mass spectrometry (UPLC-ESI-MS/MS) analysis.

**Hi-C scaffolding and chromosome assembly**

Hi-C reads were uniquely mapped to the contig assemblies and reads within 500 bp regions of Sau3AI restriction sites were retained for further analysis. Mis-joined contigs were corrected to detect abrupt long-range contact patterns by 3D-DNA pipeline. The Hi-C corrected contigs were further linked into 16 pseudo-chromosomes in PG-dip and PA-dip, and 64 pseudo-chromosomes with 4 sets of monoploid chromosomes in PA-tetra *C*. *paliurus* using the ALLHiC pipeline [3]. The accuracy of Hi-C based chromosome construction was evaluated by chromatin contact matrix (Figures S6–S8).

**Phylogenetic analysis of cytochrome P450 monooxygenases (P450s) subfamilies**

Phylogenetic analysis revealed that 22 dosage-effect genes were clustered into their respective clade for plant P450s family. Furthermore, seven genes clustered to *CYP716A* (*CpaM1st27342* and *CpaM1st17575*), *CYP71A* (*CpaM1st23492* and *CpaM1st07944*), and *CYP72A* (*CpaM1st19063*, *CpaM1st29930*, and *CpaM1st29927*) in *C*. *paliurus* were deeply compared to public databases by basic local alignment search tool (BLAST). Among them, *CpaM1st27342* and *CpaM1st17575* were defined as the ortholog genes of *CYP716A1* and *CYP716A2* in *Arabidopsi thaliana,* respectively. *CpaM1st23492* and *CpaM1st07944* were highly homologous with *CYP71A22* and *CYP71A26,* respectively. *CpaM1st19063* and *CpaM1st29927* were highly homologous with *CYP72A219*. *CpaM1st29930* was an ortholog of *CYP72A15*. For whole genome scale, *CpaM1st27346* and *CpaM1st17121* were defined as *CYP716A14v2* gene in *Artemisia annua*, and *CpaM1st38811* was an ortholog of *CYP716C* in *A. thaliana*.

**References**

[1] Dudchenko, O, Batra, SS, Arina, DO, Nyquist, SK, Hoeger M, Durand, NC, et al. *De novo* assembly of the *Aedes aegypti* genome using Hi-C yields chromosome-length scaffolds. Science 2017;356:92–5.

[2] Loureiro J, Rodriguez E, Dolezel J, Santos C. Two new nuclear isolation buffers for plant DNA flow cytometry: a test with 37 species. Ann Bot 2007;100:875–88.

[3] Zhang X, Zhang S, Zhao Q, Ming R, Tang H. Assembly of allele-aware, chromosomal-scale autopolyploid genomes based on Hi-C data. Nat Plants 2019;5:833–45.
