# Supplementary figures and images for: Whole-genome Duplication Reshaped Adaptive Evolution in A Relict Plant Species, Cyclocarya paliurus
Source: Genomics Proteomics Bioinformatics. 2023 Feb 11;21(3):455–69. doi: 10.1016/j.gpb.2023.02.001 (PMC10787019; doi:10.1016/j.gpb.2023.02.001)

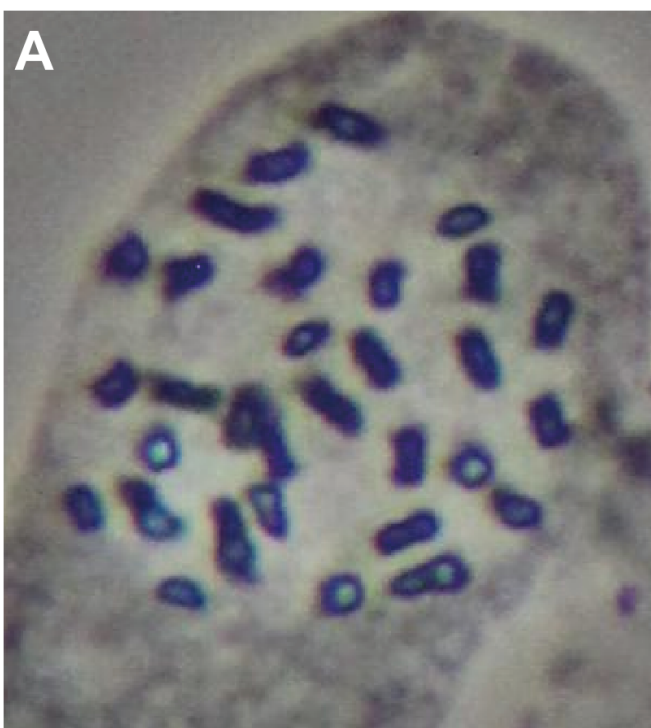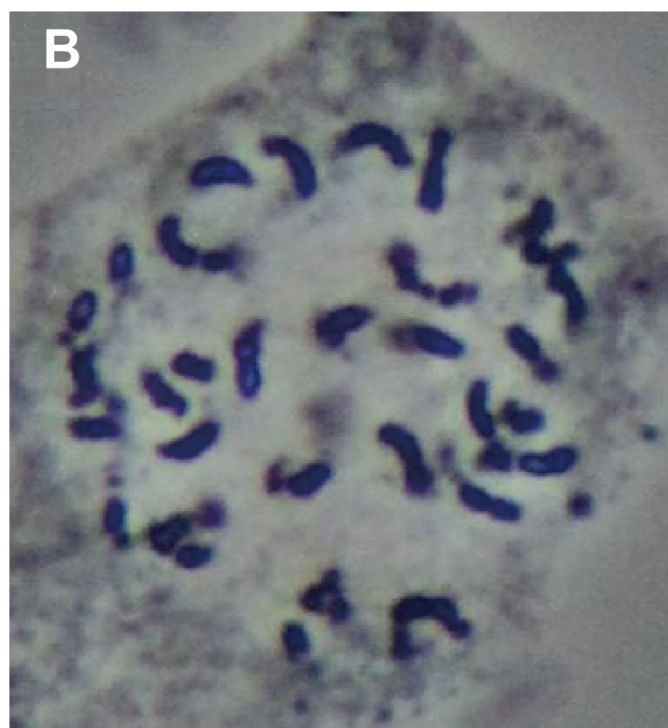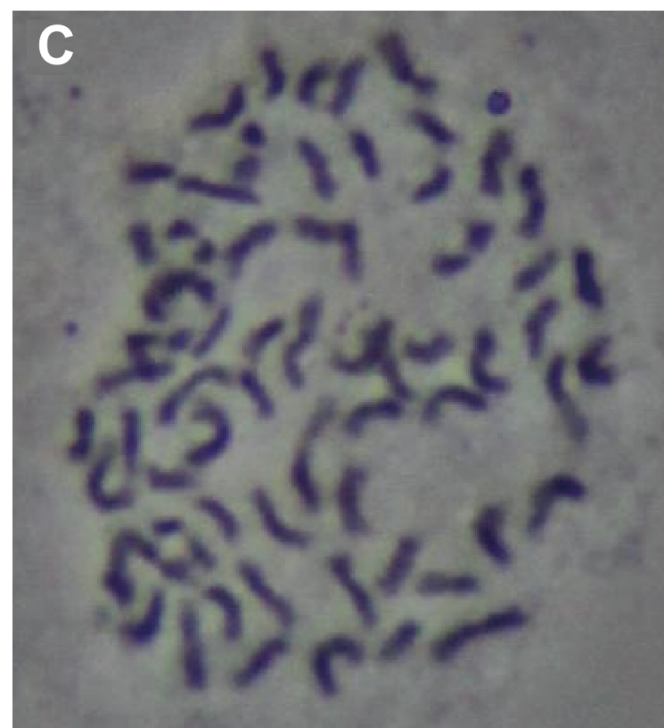

Supplement: Supplementary Figure S1 — Chromosome karyotypes of C. paliurus A. PA-dip. B. PG-dip. C. PA-tetra. [file mmc2.pdf]

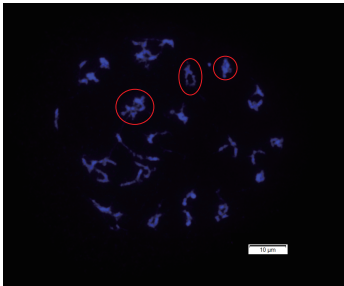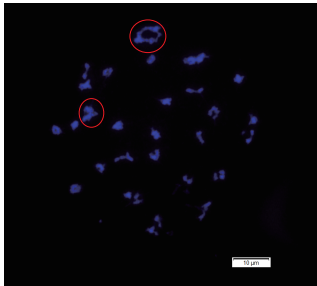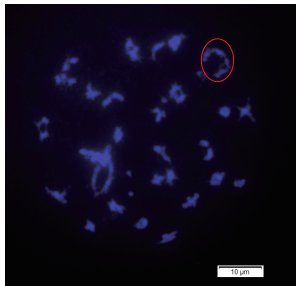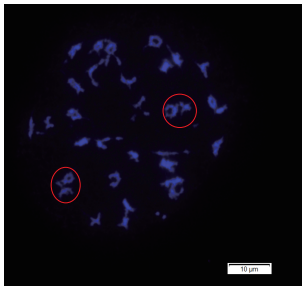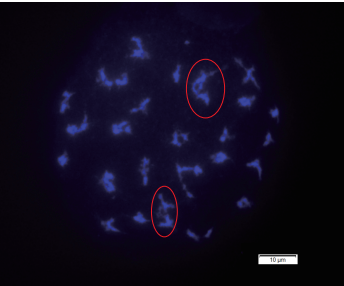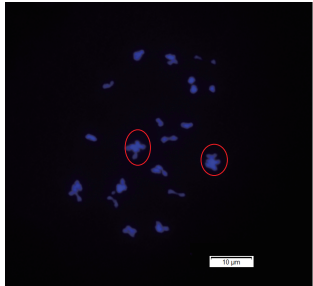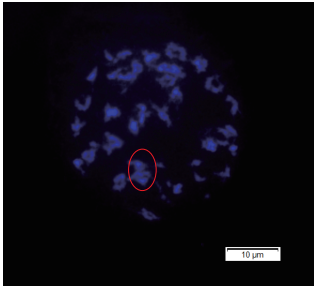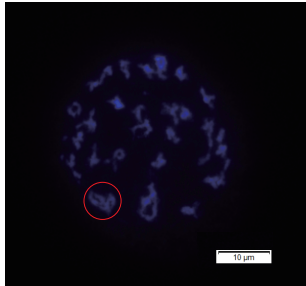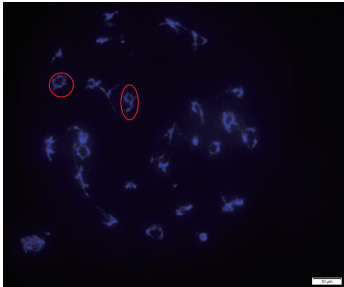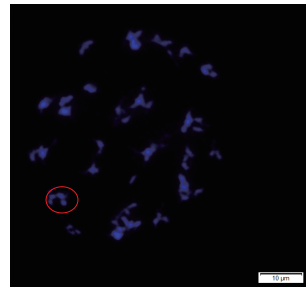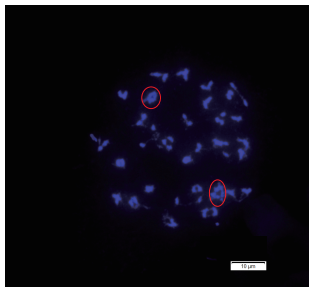

Supplement: Supplementary Figure S2 — The phenomena of homologous chromosomesynapsis at the early stage of meiosis Ⅰ in PA-tetra C. paliurus A total of 11 pollen mother cells were selected to observe and the red rounds represent the quadrivalent. [file mmc3.pdf]

**A**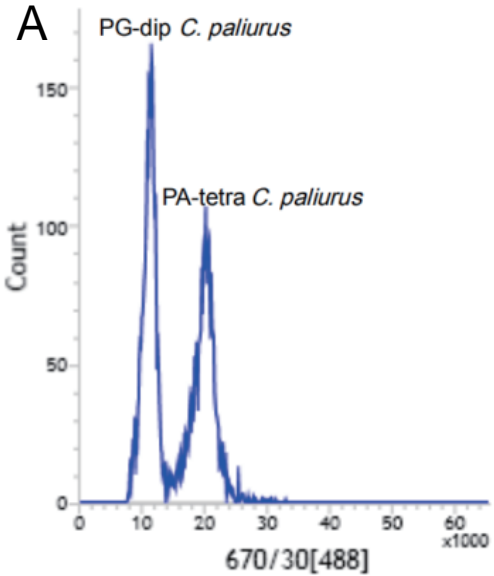**B**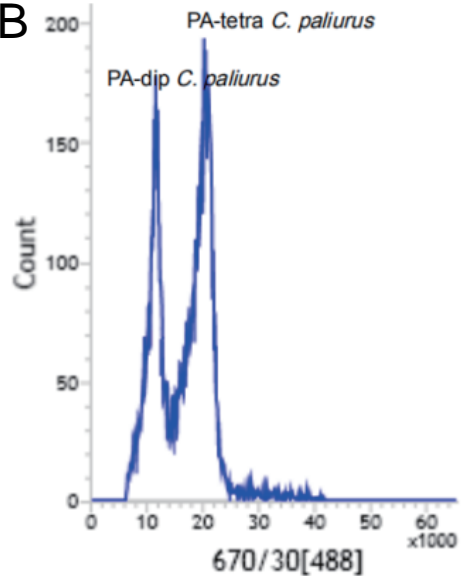**C**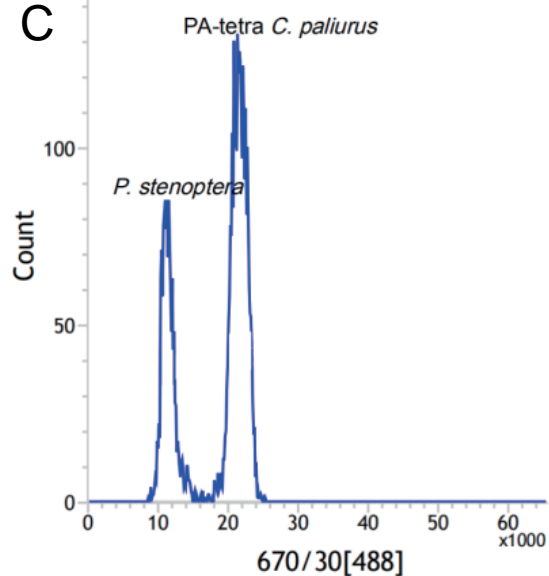

Supplement: Supplementary Figure S3 — Using flow cytometry to estimate genome size of C. paliurus A. PG-dip. B. PA-dip. C. PA-tetra. The P. stenoptera genome (2n = 2× = ∼ 600 Mb) and PA-tetra C. paliurus were used as an internal reference standard. [file mmc4.pdf]

A

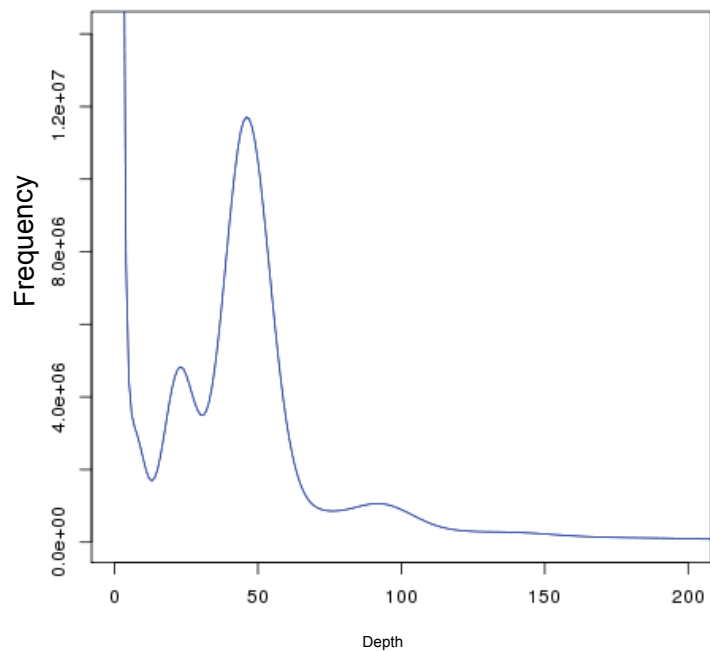

B

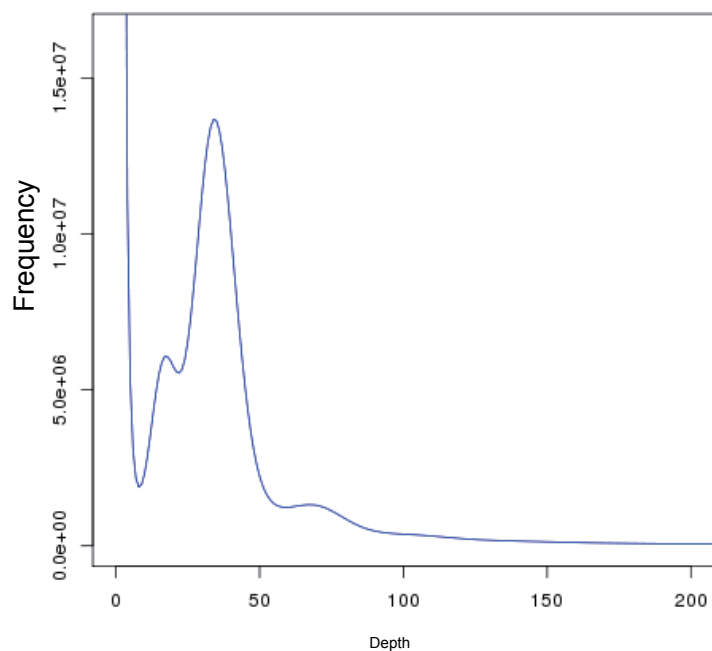

C

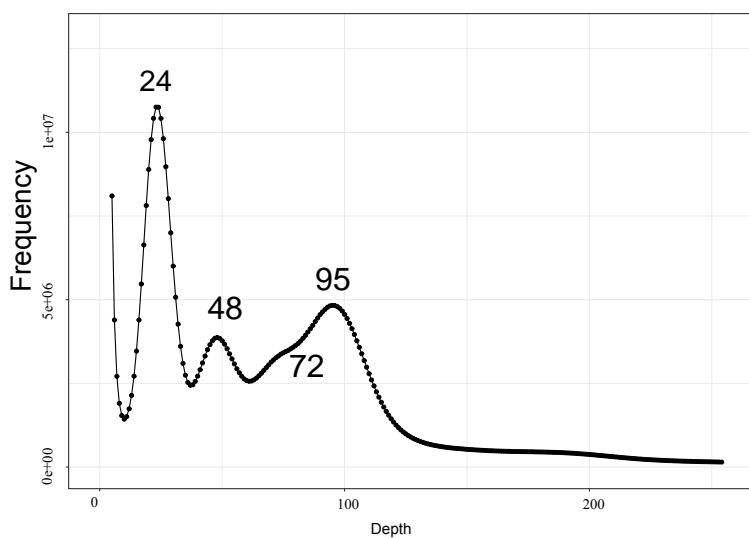

D

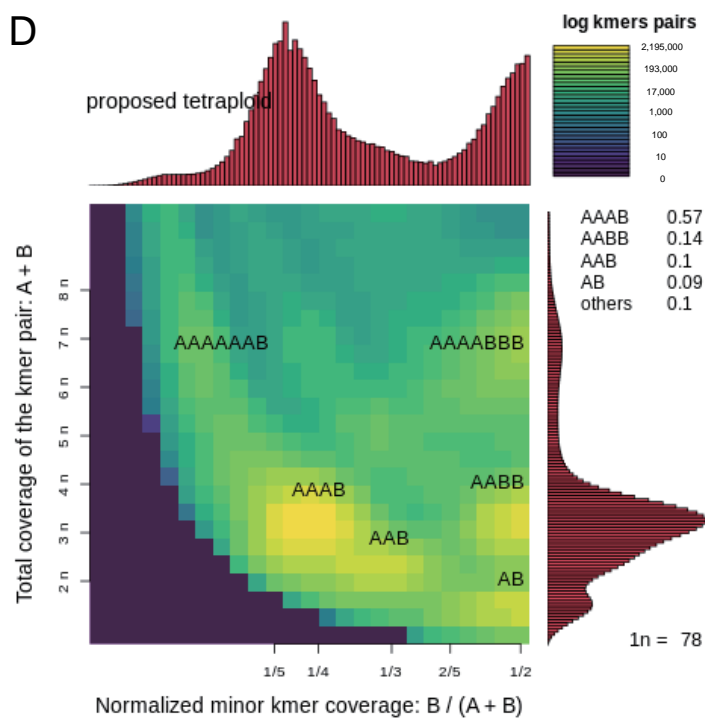

Supplement: Supplementary Figure S4 — K-mer (21-mer) distribution and estimation of genome size and heterozygosity of C. paliurus A. PG-dip. B. PA-dip. C. PA-tetra. D. Total coverage of the k-mer pair (A + B) in PA-tetra C. paliurus. [file mmc5.pdf]

A

Pathway

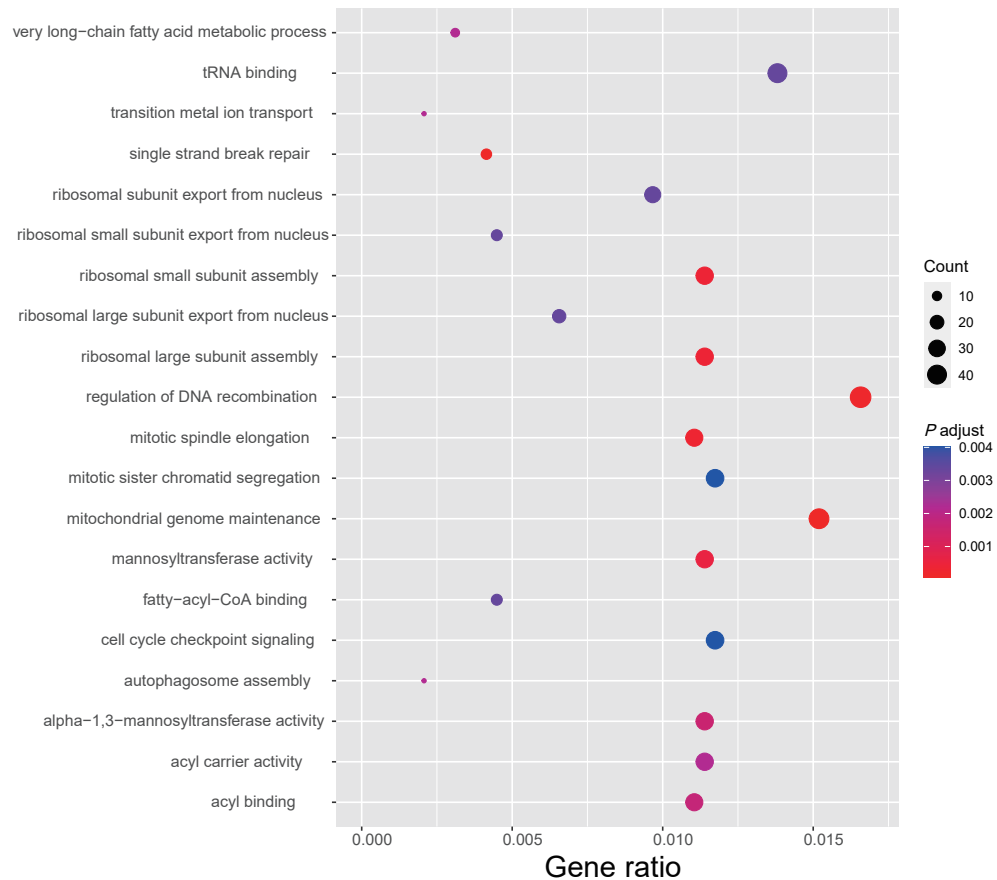

B

Description

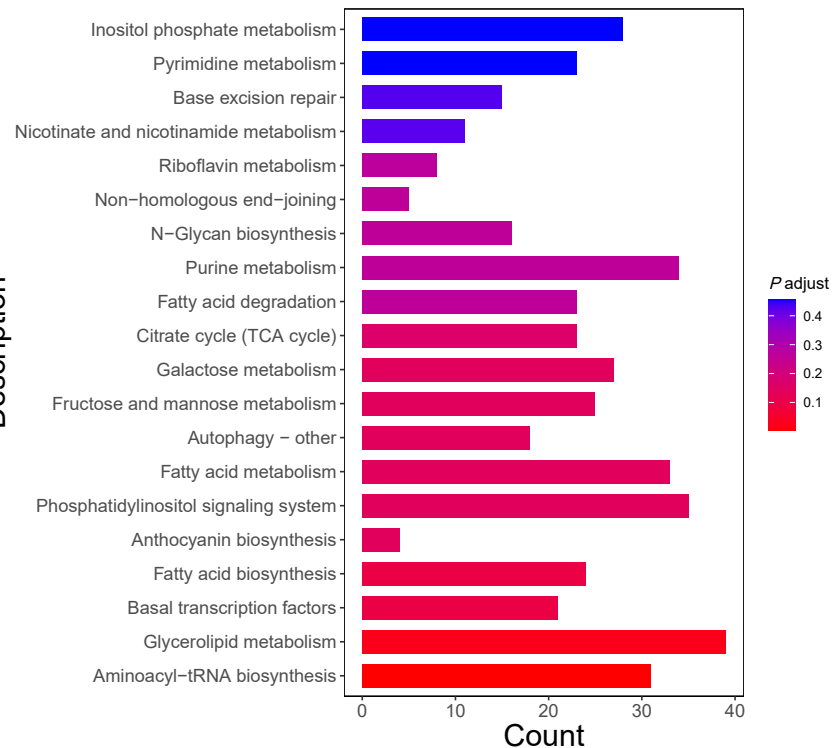

Supplement: Supplementary Figure S5 — Functional enrichment analysis of selected genes involved in haplotypic variations in PA-tetra genome A. GO. B. KEGG. GO, Gene Ontology; KEGG, Kyoto Encyclopedia of Genes and Genomes. [file mmc6.pdf]

Whole\_genome\_150K

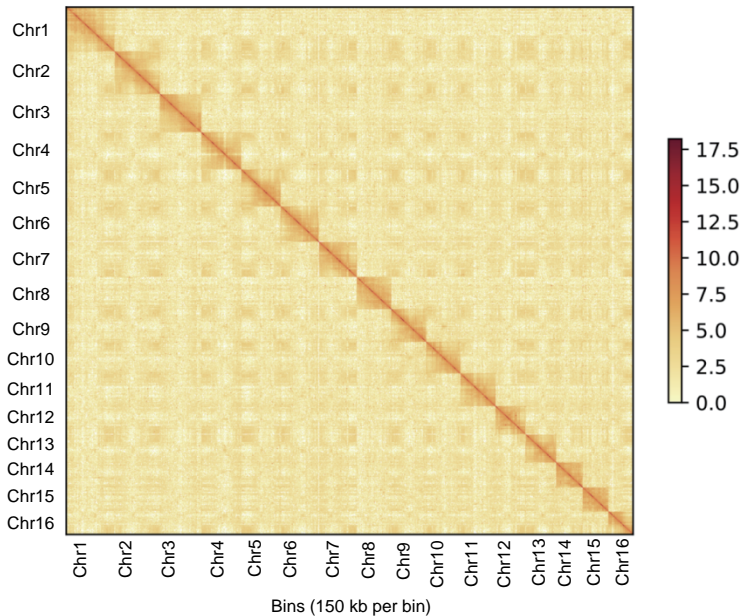

Supplement: Supplementary Figure S6 — Genome-wide analysis of chromatin interactions at 150-kb resolution in PA-dip genome The colored bar on the right represents the strength of interaction. [file mmc7.pdf]

Whole\_genome\_150K

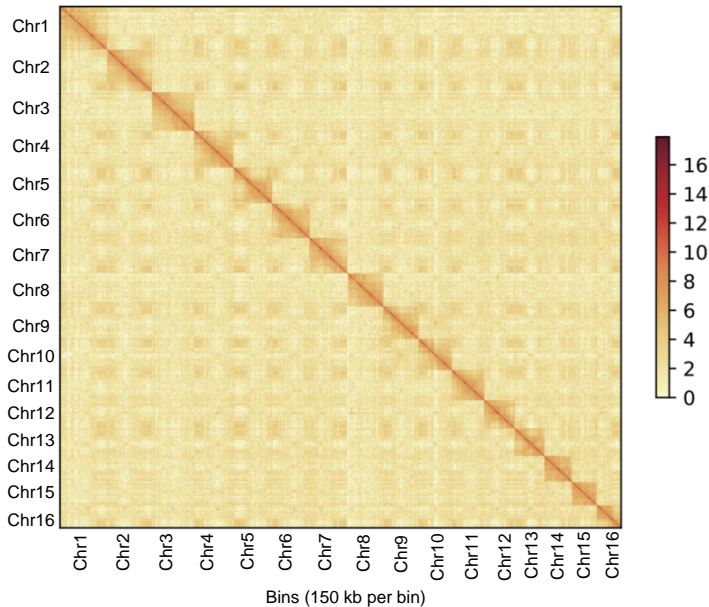

Supplement: Supplementary Figure S7 — Genome-wide analysis of chromatin interactions at 150-kb resolution in PG-dip genome [file mmc8.pdf]

# Whole\_genome\_150K

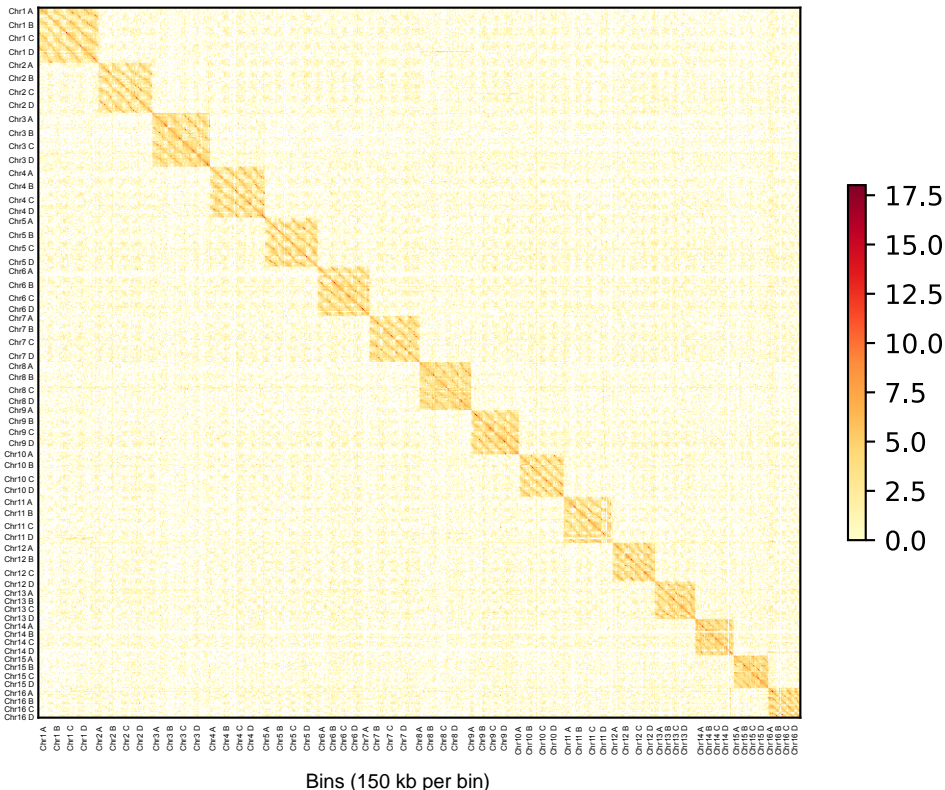

Supplement: Supplementary Figure S8 — Genome-wide analysis of chromatin interactions at 150-kb resolution in PA-tetra genome [file mmc9.pdf]

Inter-genomic comparison: PA vs. PG (30,536 gene pairs)

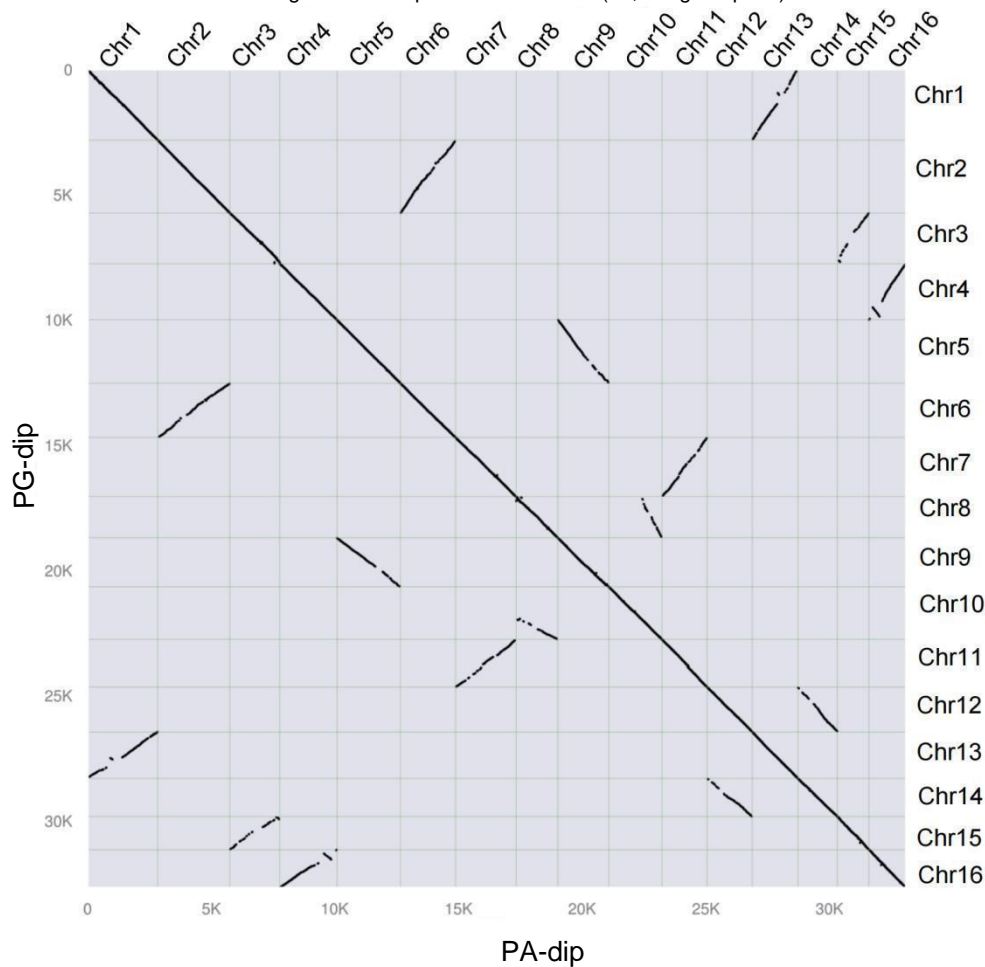

Supplement: Supplementary Figure S9 — Synteny analysis between PA-dip and PG-dip genomes [file mmc10.pdf]

A

Pathway

C

Pathway

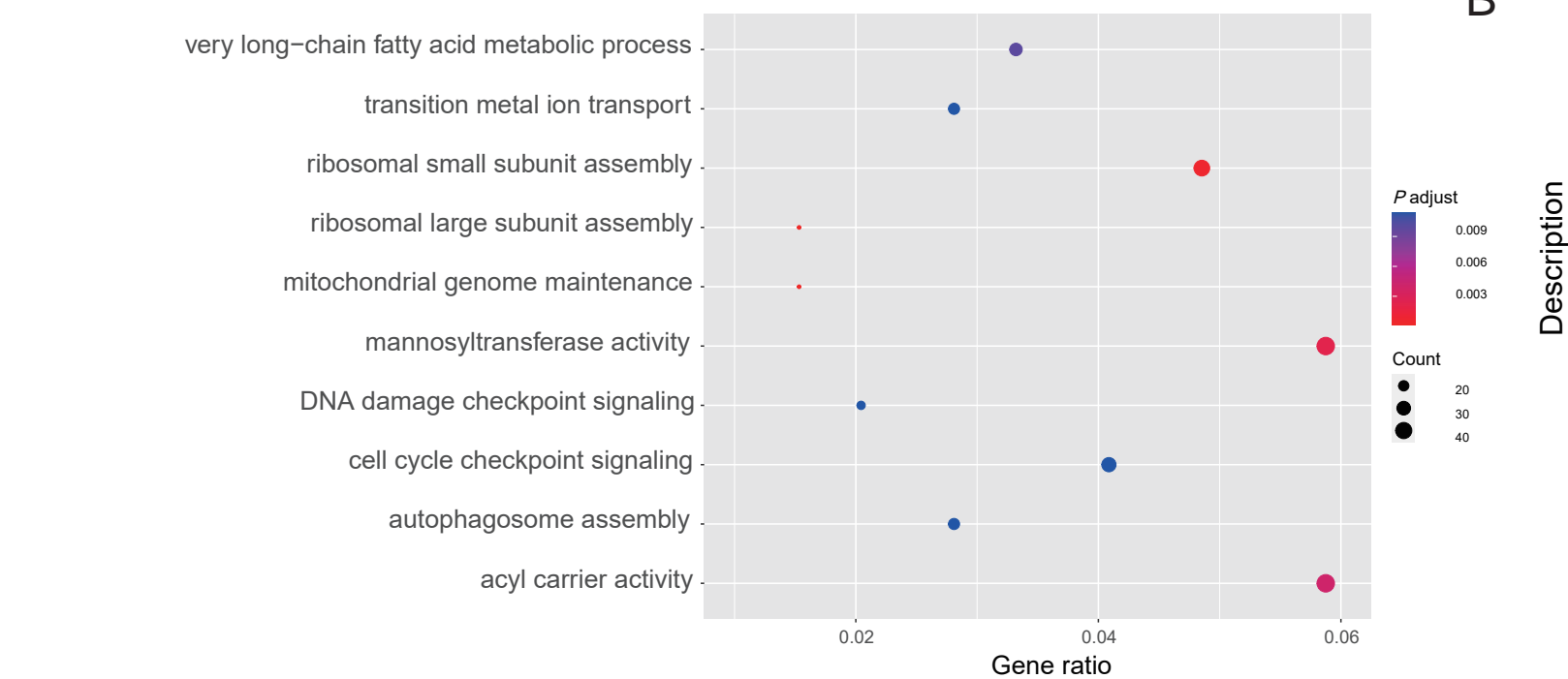

B

Description

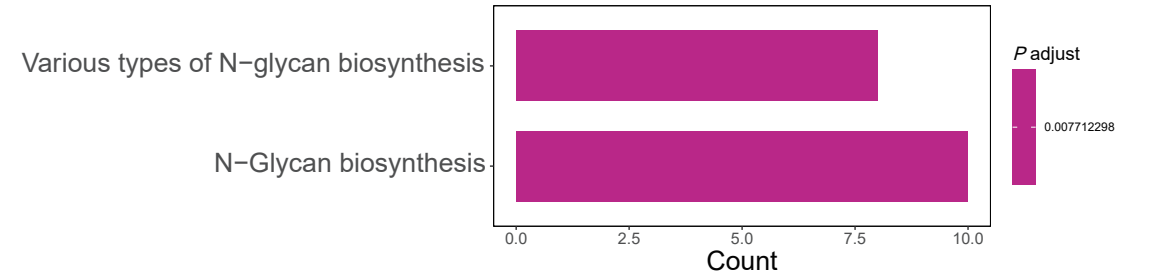

D

Description

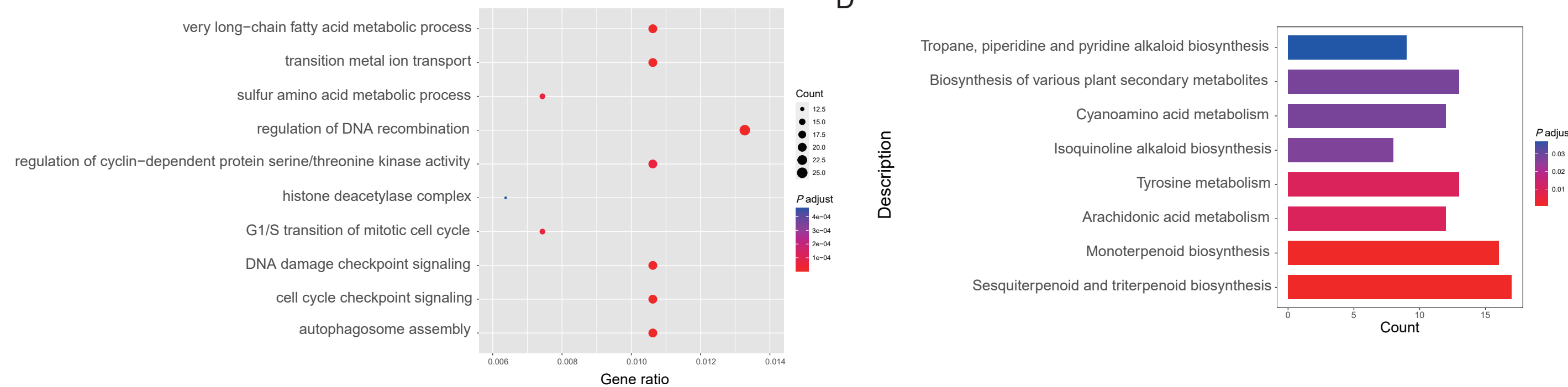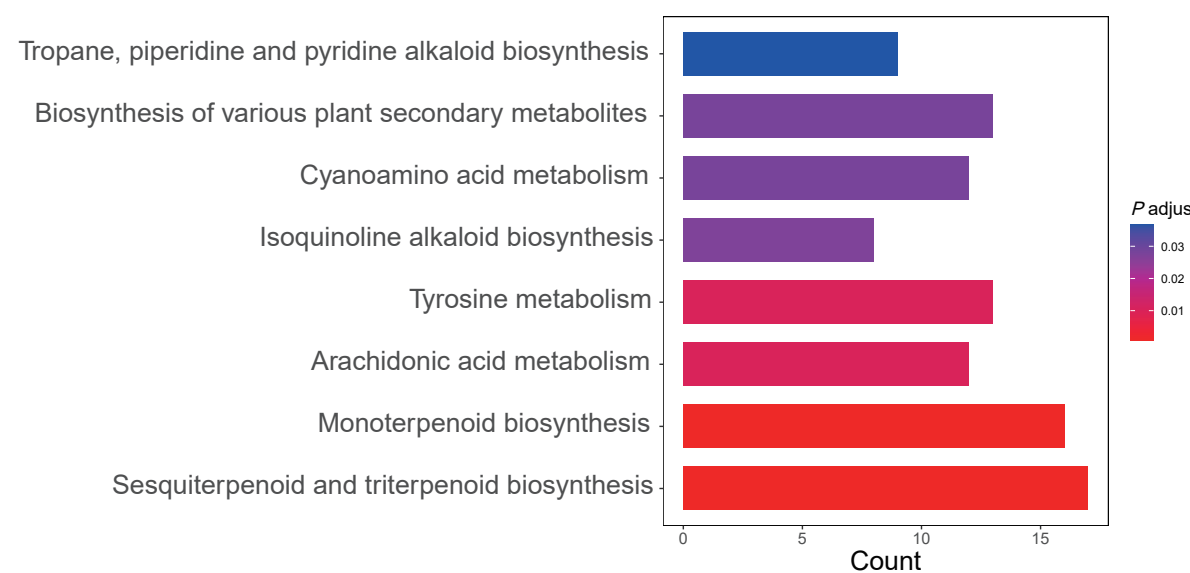

Supplement: Supplementary Figure S10 — Functional enrichment analysis of selected genes involved in WGD1 and WGD2 events A. GO enrichment analysis of selected genes involved in WGD1 event. B. KEGG enrichment analysis of selected genes involved in WGD1 event. C. GO enrichment analysis of selected genes involved in WGD2 event. D. KEGG enrichment analysis of selected genes involved in WGD2 event. [file mmc11.pdf]

Inter-genomiccomparison:PA-dip vs. *Vitis vinifera*(11,858genepairs)

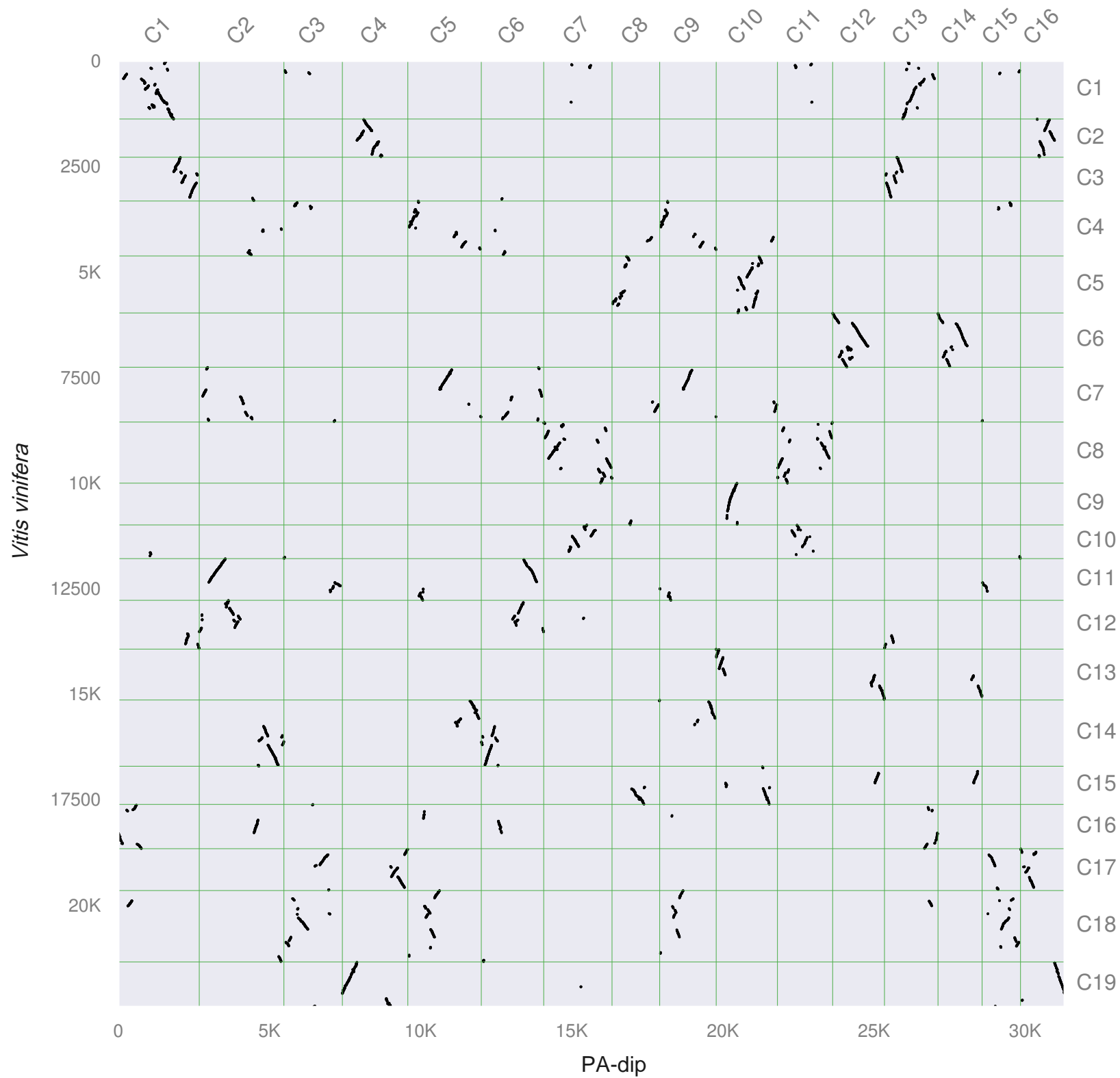

Supplement: Supplementary Figure S11 — Synteny analysis between PA-dip andV. vinifera genomes [file mmc12.pdf]

Inter-genomiccomparison:PA-tetra vs. *Vitis vinifera* (26,245genepairs)

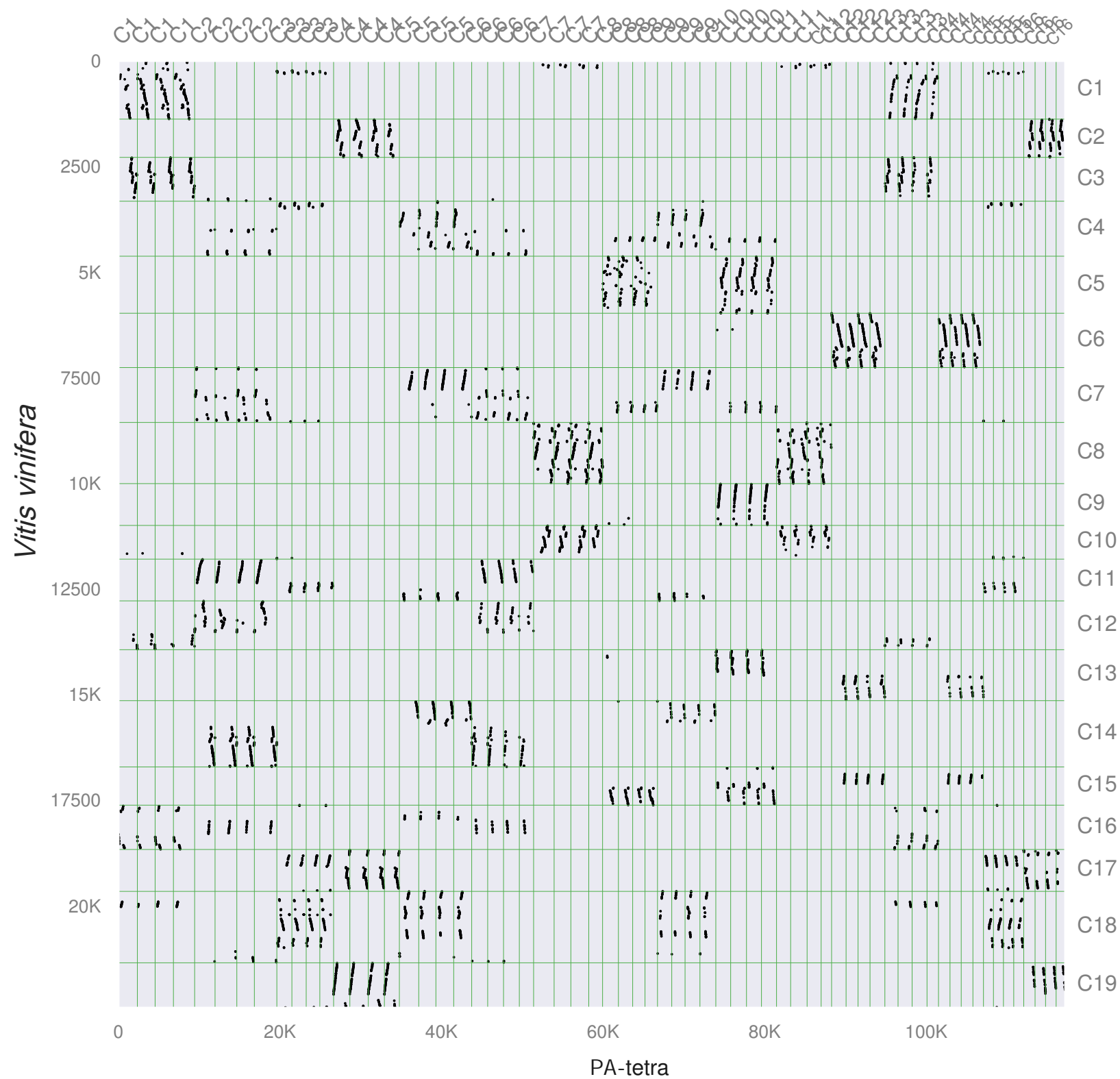

Supplement: Supplementary Figure S12 — Synteny analysis between PA-tetra andV. vinifera genomes [file mmc13.pdf]

PA-dip PA-tetra PG-dip

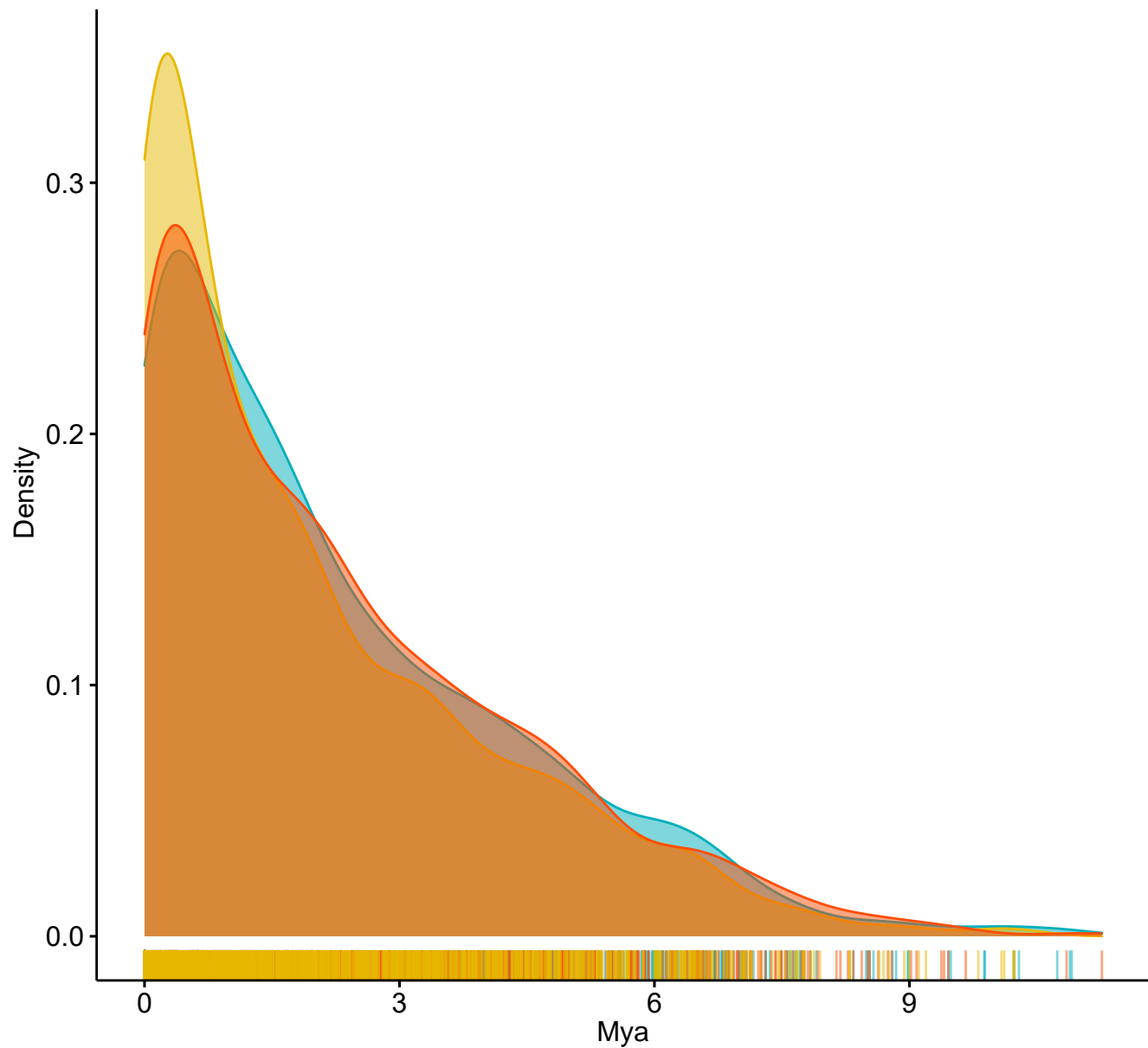

Supplement: Supplementary Figure S13 — Comparison of timing of LTR-RT insertions among PA-tetra, PA-dip, and PG-dip genomes LTR-RT, long terminal repeat retrotransposon. [file mmc14.pdf]

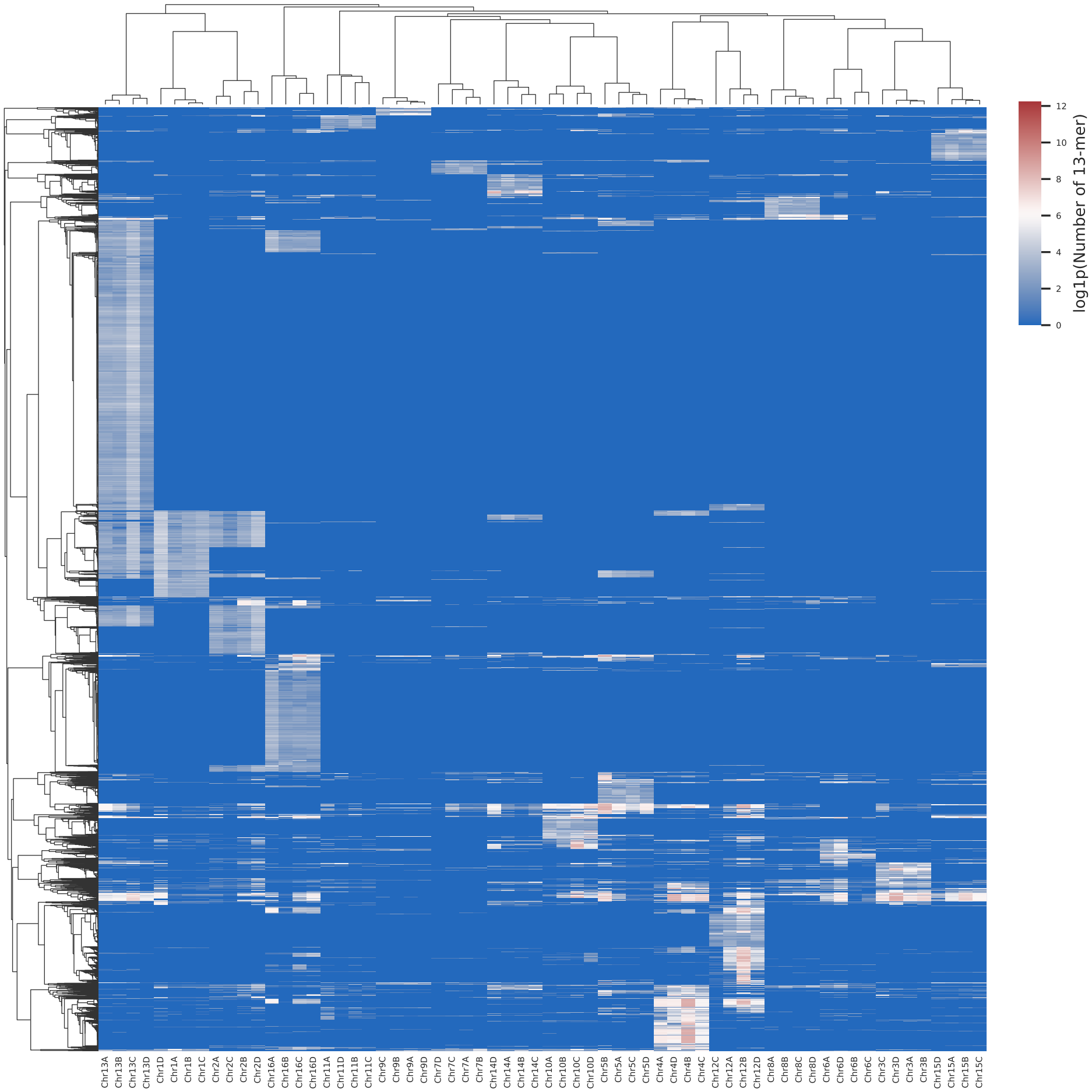

Supplement: Supplementary Figure S14 — Clustering of counts ofK-mers (K = 13) enables the consistent partitioning of four haplotypes in each homologous chromosome into same group [file mmc15.pdf]

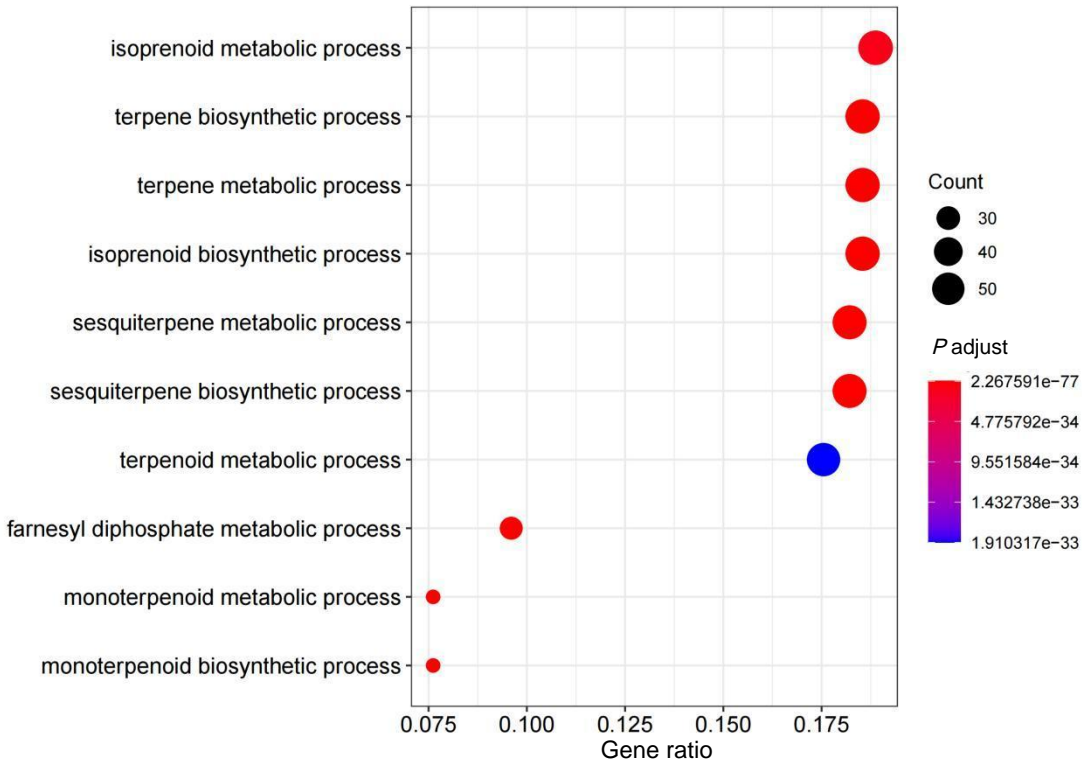

Supplement: Supplementary Figure S15 — GO enrichment analysis of genes experienced expansion inC. paliurus [file mmc16.pdf]

Description

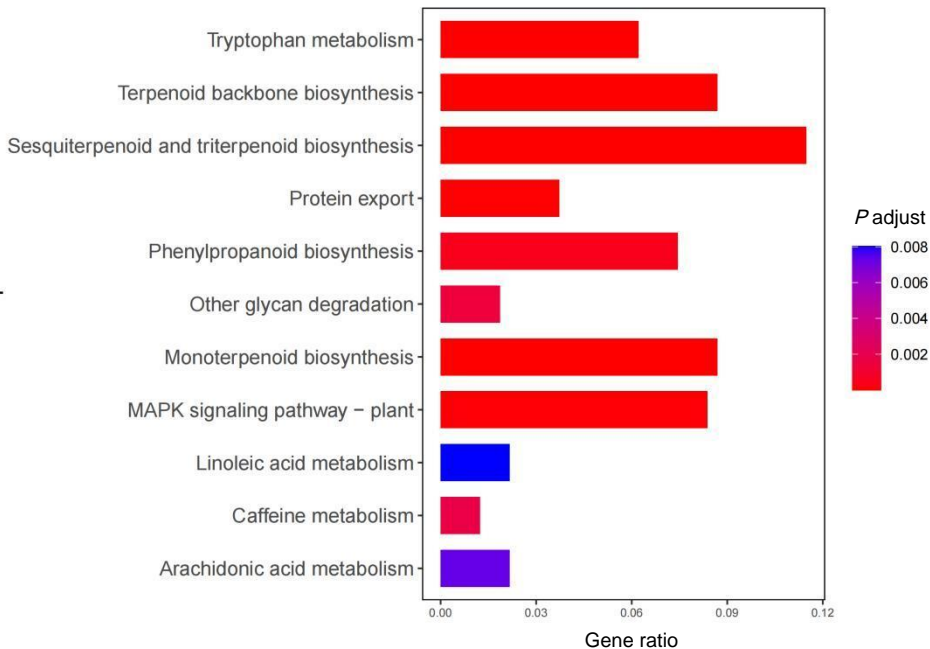

Supplement: Supplementary Figure S16 — KEGG pathway analysis of genes experienced expansion inC. paliurus [file mmc17.pdf]

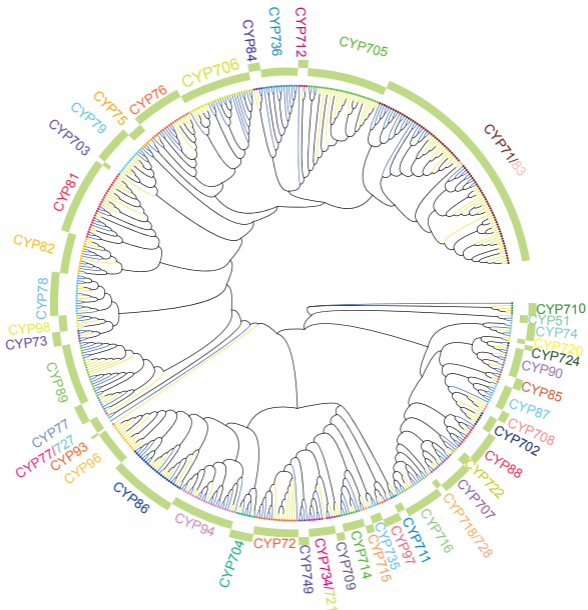

Supplement: Supplementary Figure S18 — Phylogenic analysis of P450 families The yellow and blue branches indicate the sequences from Arabidopsis and C. paliurus, respectively. The dots represent P450 genes. The outermost arc indicates the P450 gene family. [file mmc19.pdf]

Pathway

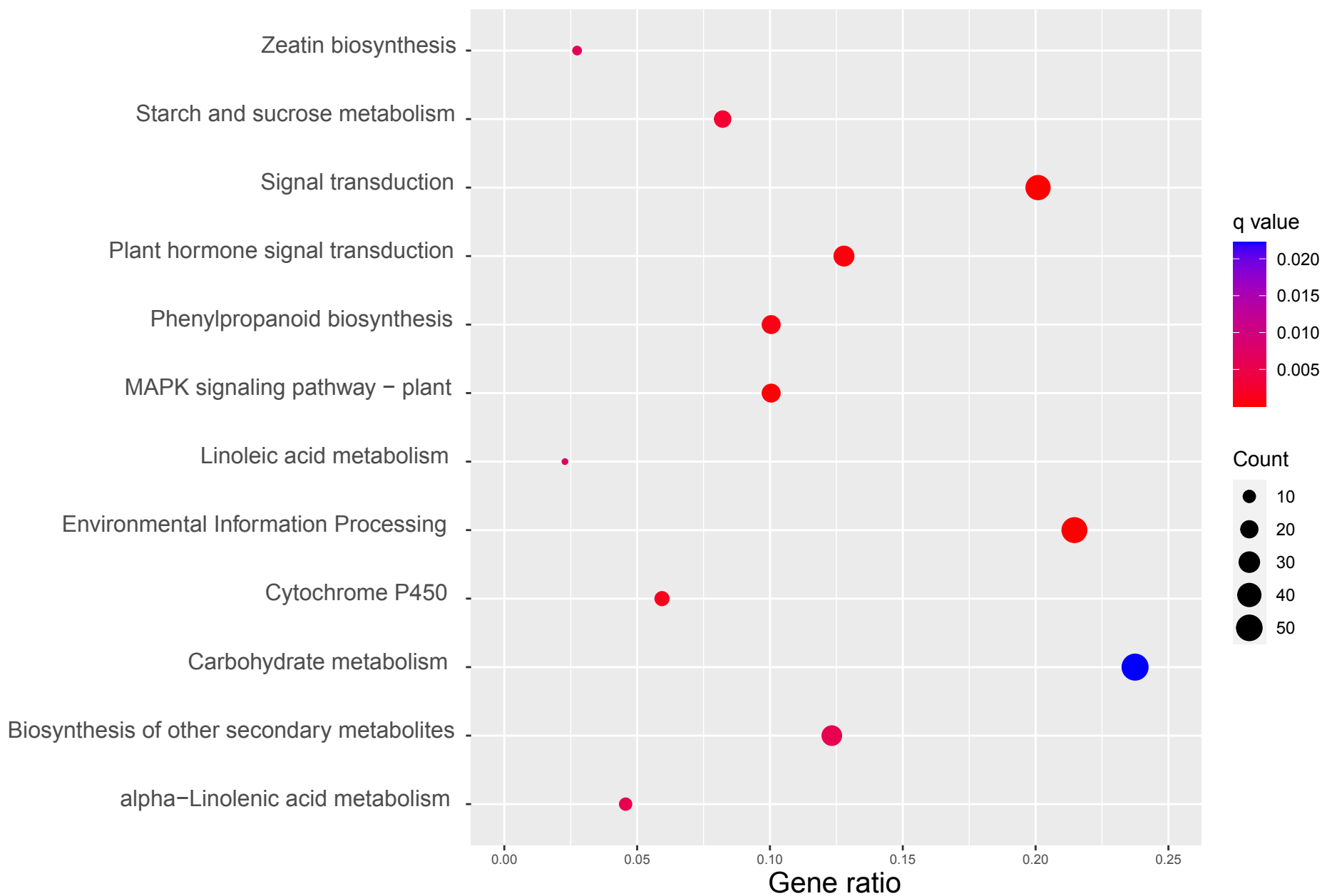

Supplement: Supplementary Figure S20 — .KEGG enrichment of the 958 DEGs in female floral buds (PG-F vs. PA-F) at five flowering time stages (S0–S4) [file mmc21.pdf]

Pathway

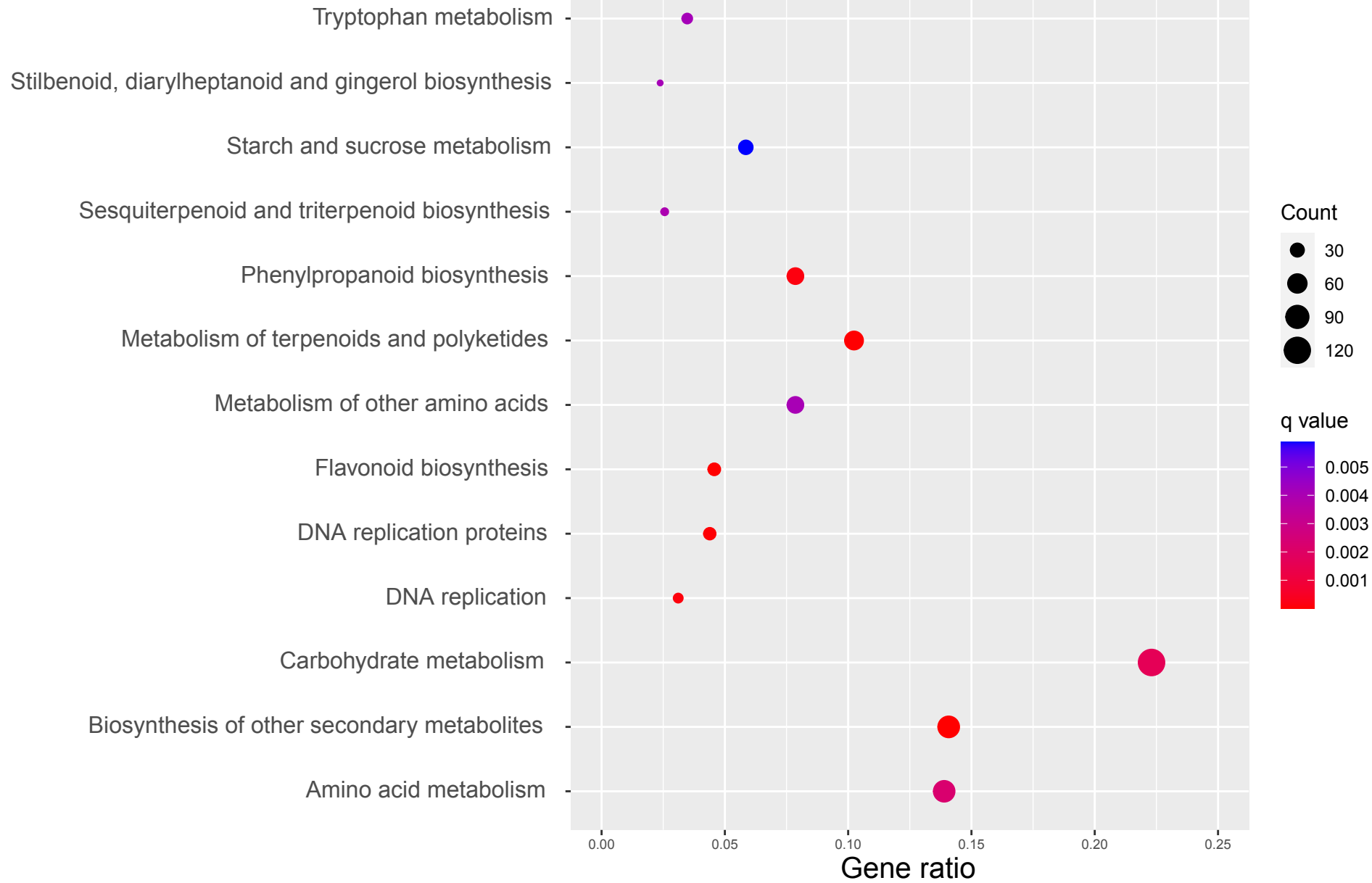

Supplement: Supplementary Figure S21 — KEGG enrichment of the 2373 DEGs in male floral buds (PA-M vs. PG-M) at five flowering time stages (S0–S4) [file mmc22.pdf]

A

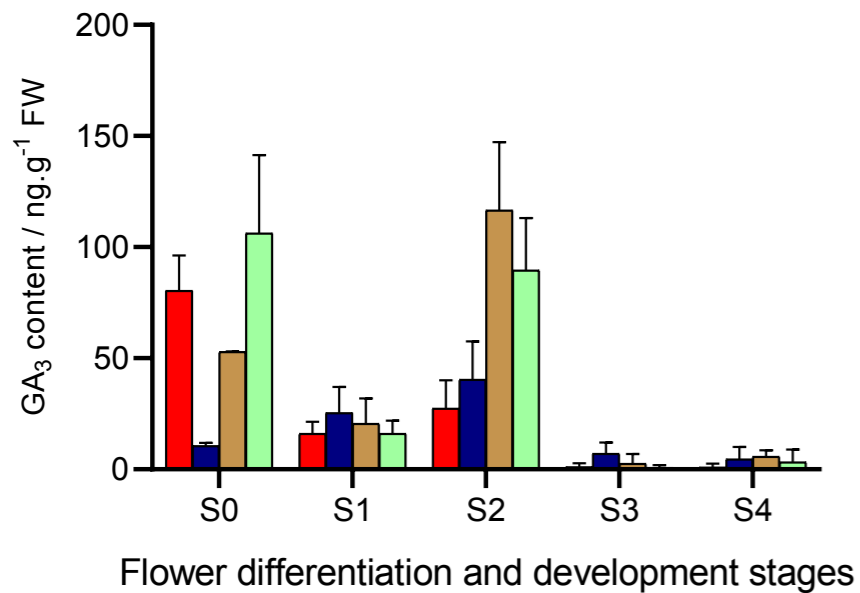

B

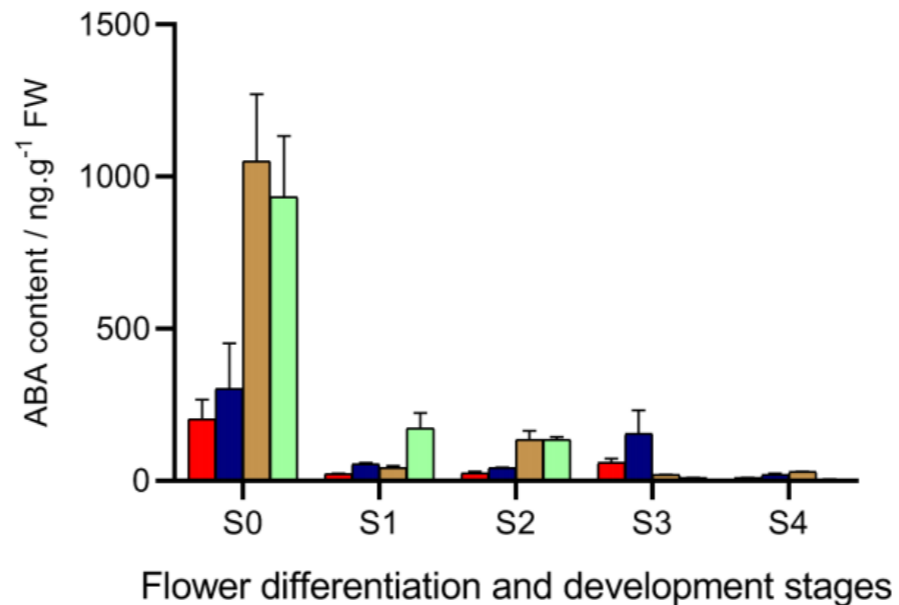

C

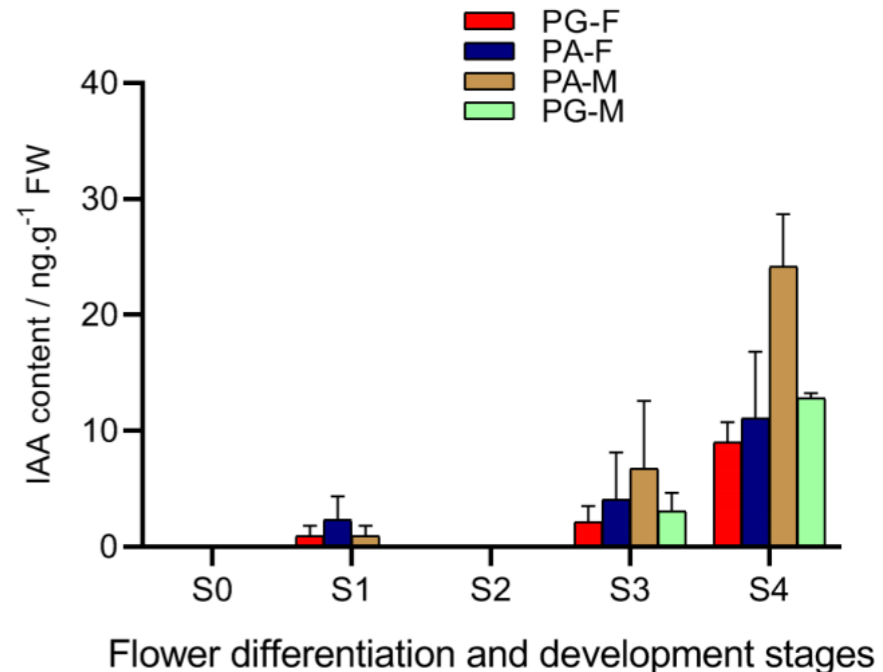

Supplement: Supplementary Figure S22 — Hormone contents of C. paliurus during five flower differentiation and development stages A. Identification of GA3 level in female and male floral buds from PG or PA type. B. Identification of ABA level in female and male floral buds from PG or PA type. C. Identification of IAA level in female and male floral buds from PG or PA type. FW, fresh weight; GA3, gibberellin; ABA, abscisic acid; IAA, auxin. [file mmc23.pdf]

Cluster dendrogram

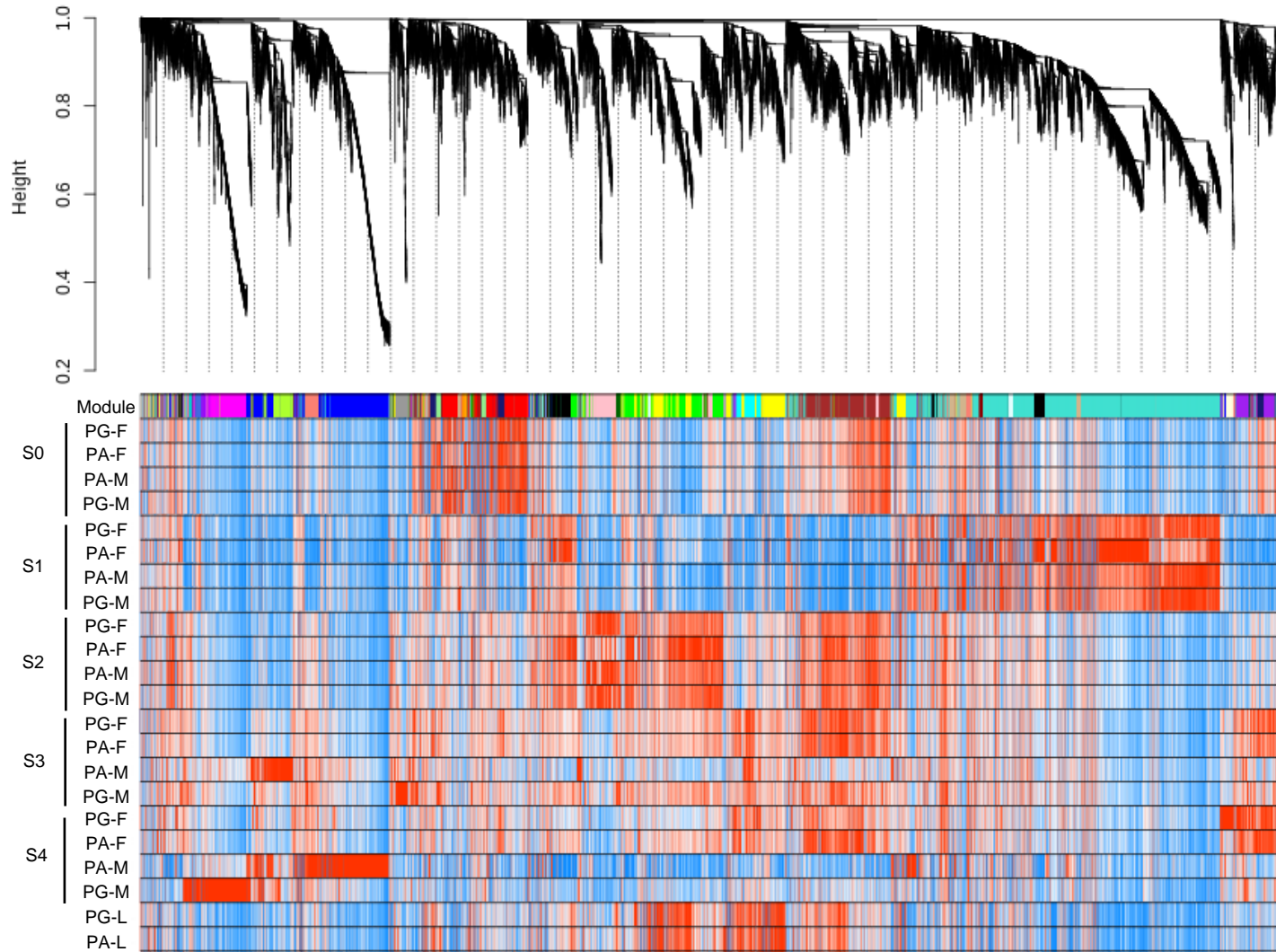

Supplement: Supplementary Figure S23 — Hierarchical clustering tree (dendrogram) of genes based on co-expression network analysis in PG and PA individuals (female flora buds, male flora buds, female leaves, and male leaves) during five development stages Each individual’s value is the average of the expression of three replicate samples. PG-L means leaves of PG and PA-L means leaves of PA. [file mmc24.pdf]

D

Red (1244)

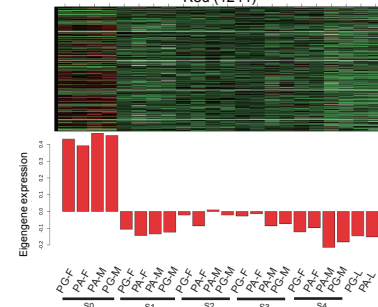

F

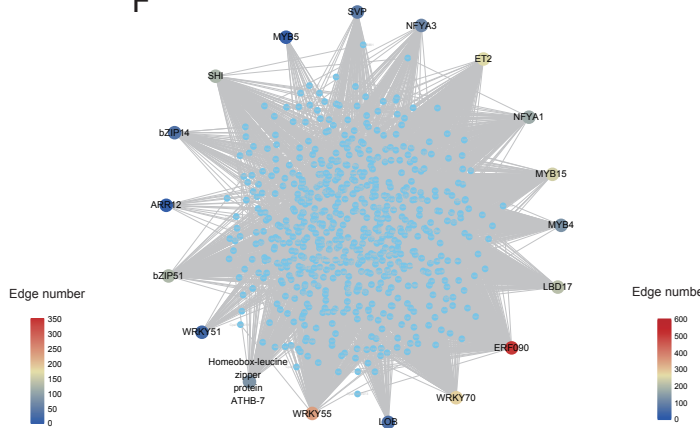

Supplement: Supplementary Figure S25 — Functions and networks of co-expression module genes A. Module–trait relationships. The column indicate GA3, and the rows indicate the different modules. The red and blue colors indicate positive and negative correlations, respectively. The correlation coefficient (r) and P value (P) are displayed in each cell. B. Eigengene expression profiles in the darkorange module. C. Eigengene expression profiles in the pink module. D. Eigengene expression profiles in the red module. E. Construction of the correlation network of the pink module. F. Construction of the correlation network of the red module. The large circles represent transcription factors. The color is determined by the edge number of the gene. [file mmc26.pdf]

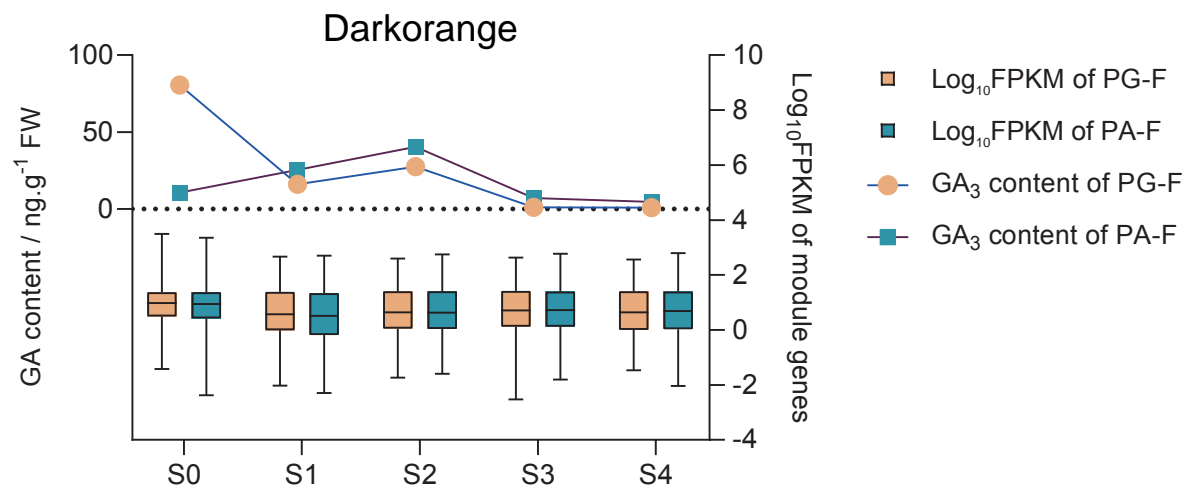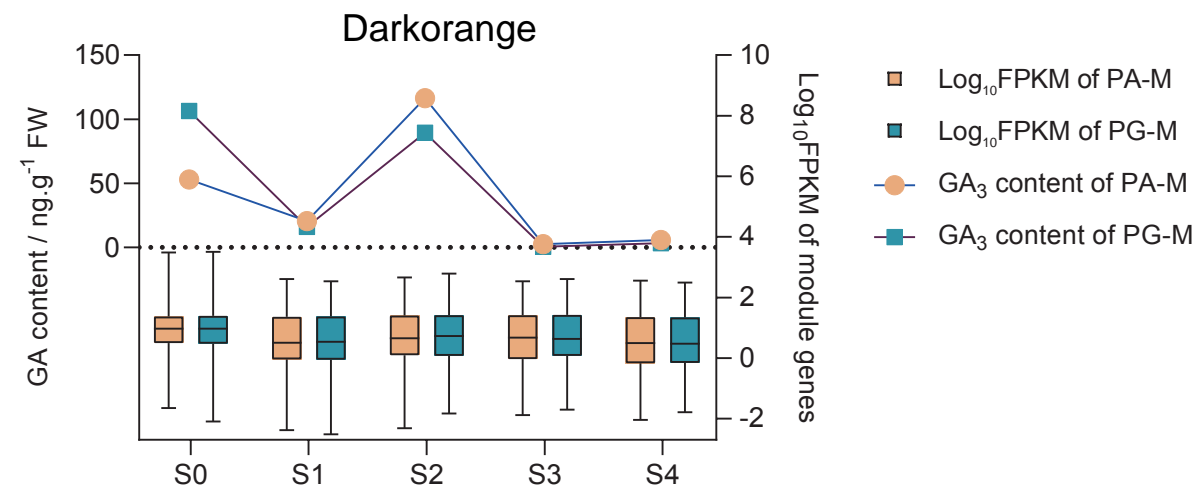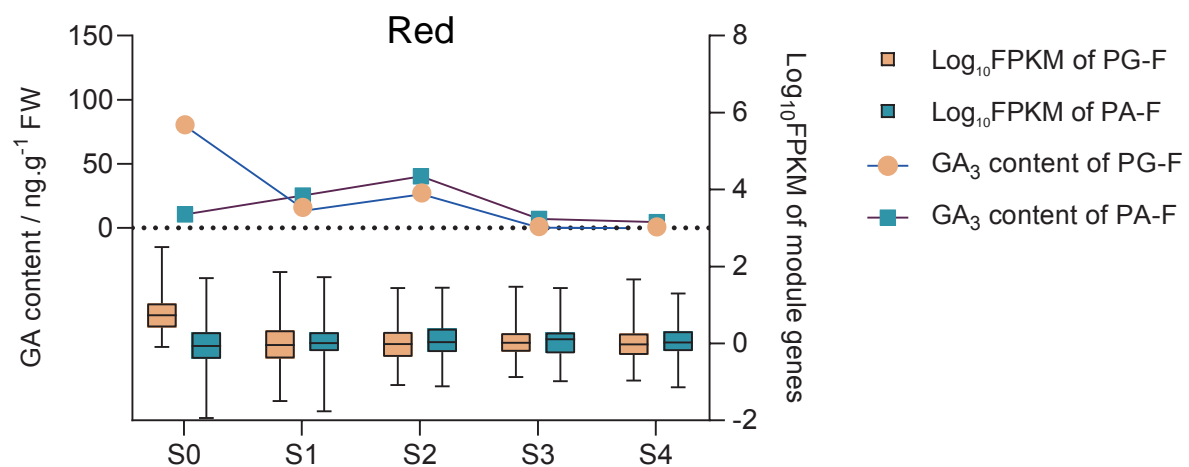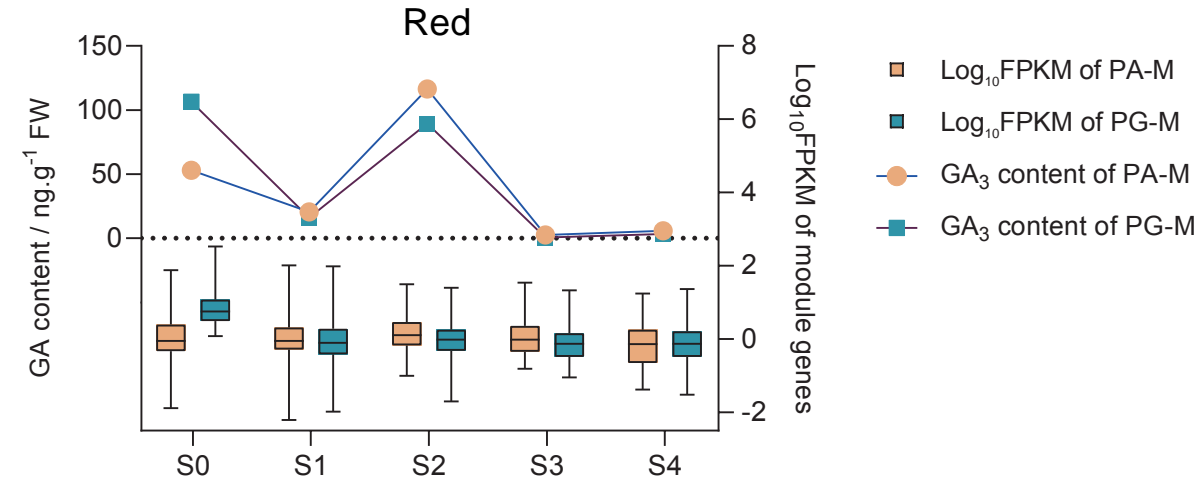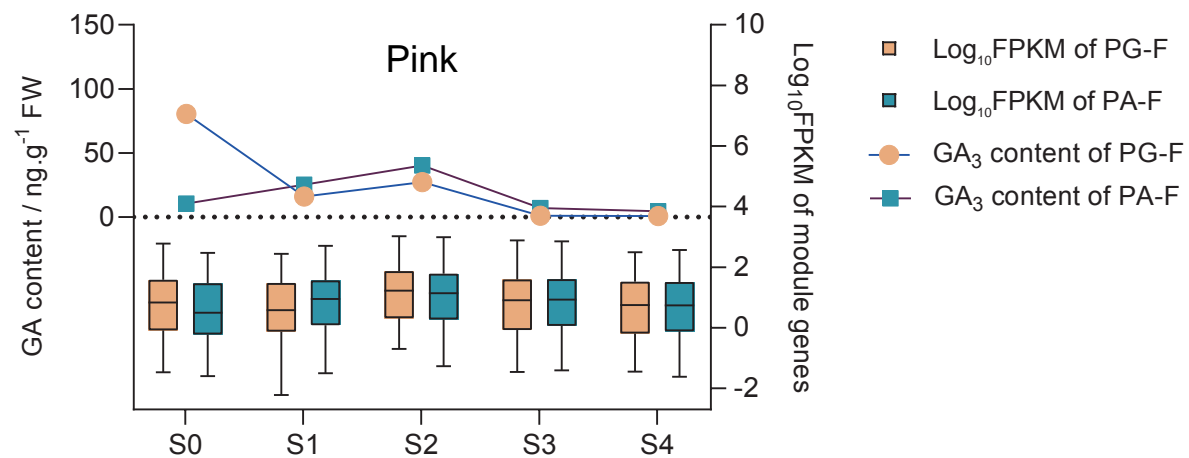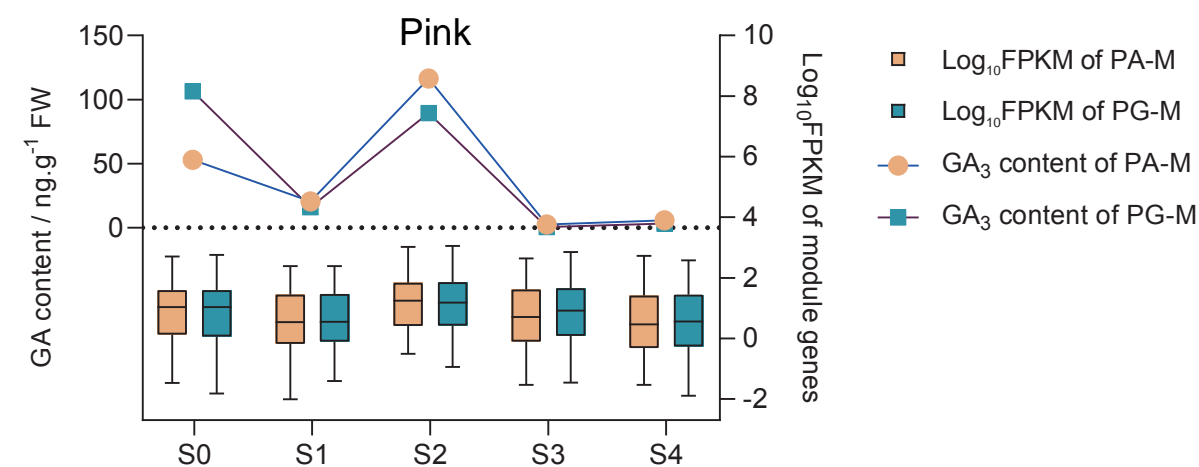

Supplement: Supplementary Figure S26 — Expression profiles of three modules and GA3 contents during five stages [file mmc27.pdf]

Pathway

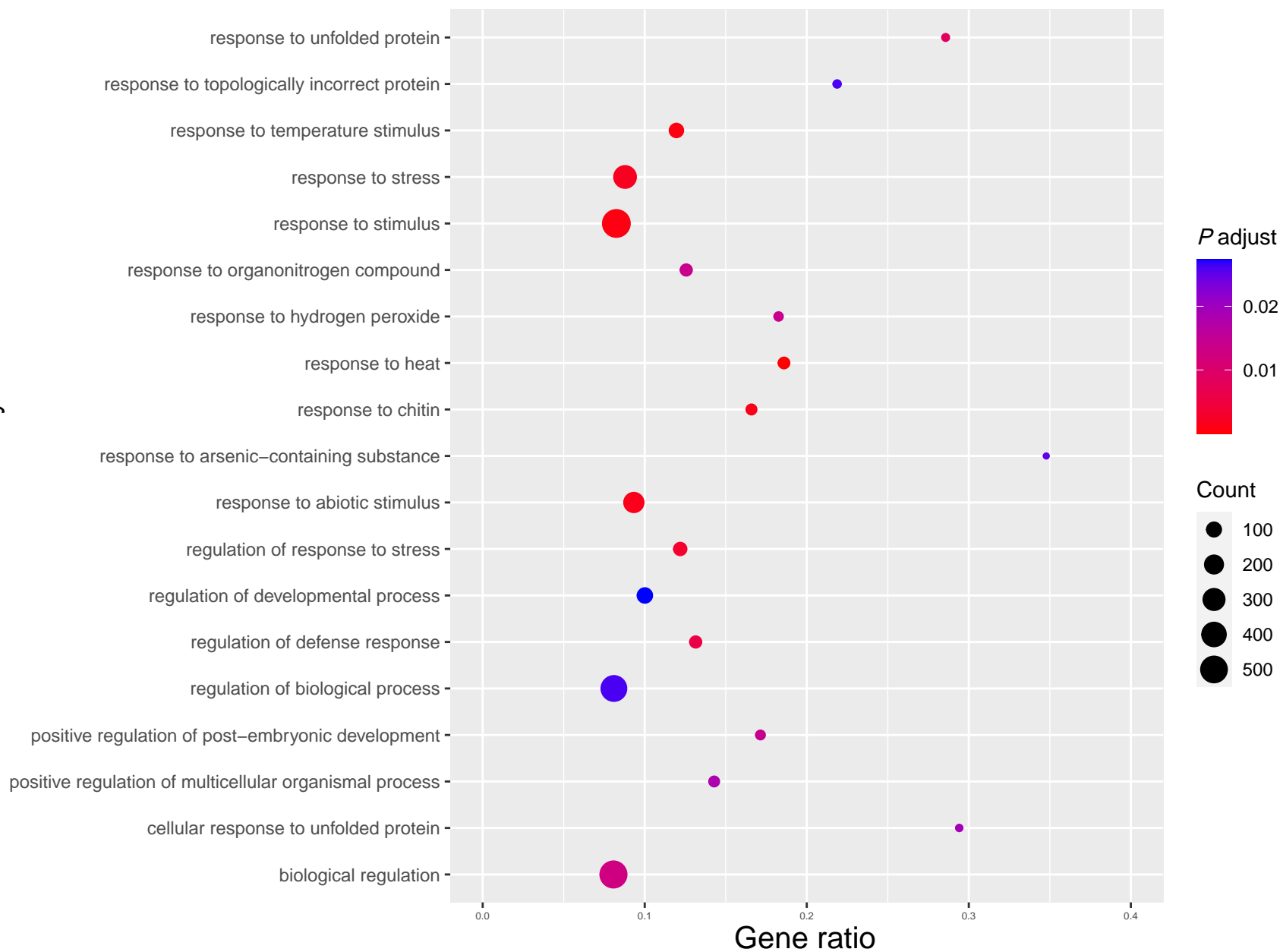

Supplement: Supplementary Figure S27 — GO enrichment of three modules (darkorange, red, and pink) genes [file mmc28.pdf]

Pathway

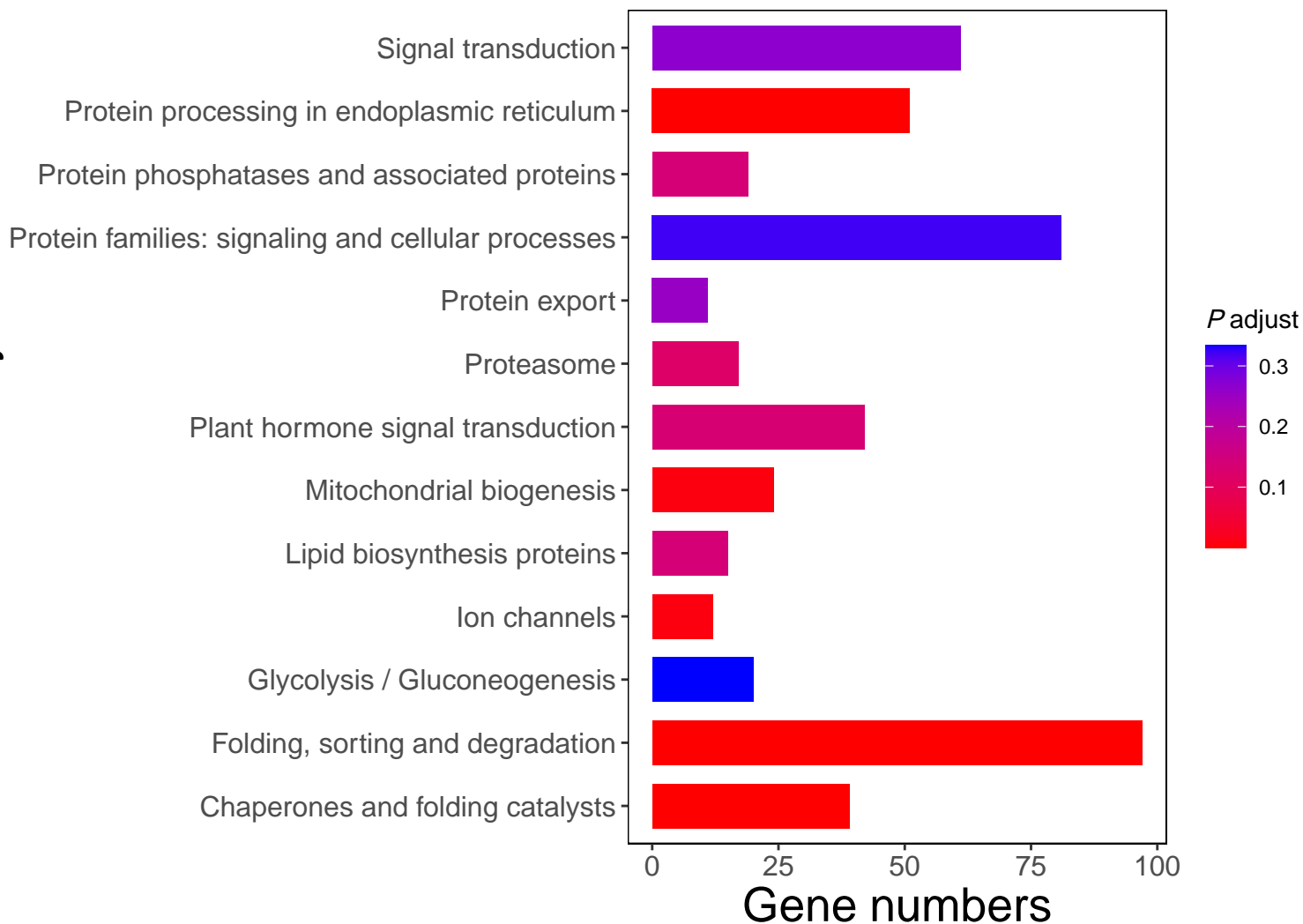

Supplement: Supplementary Figure S28 — KEGG enrichment of the three modules (darkorange, red, and pink) genes [file mmc29.pdf]

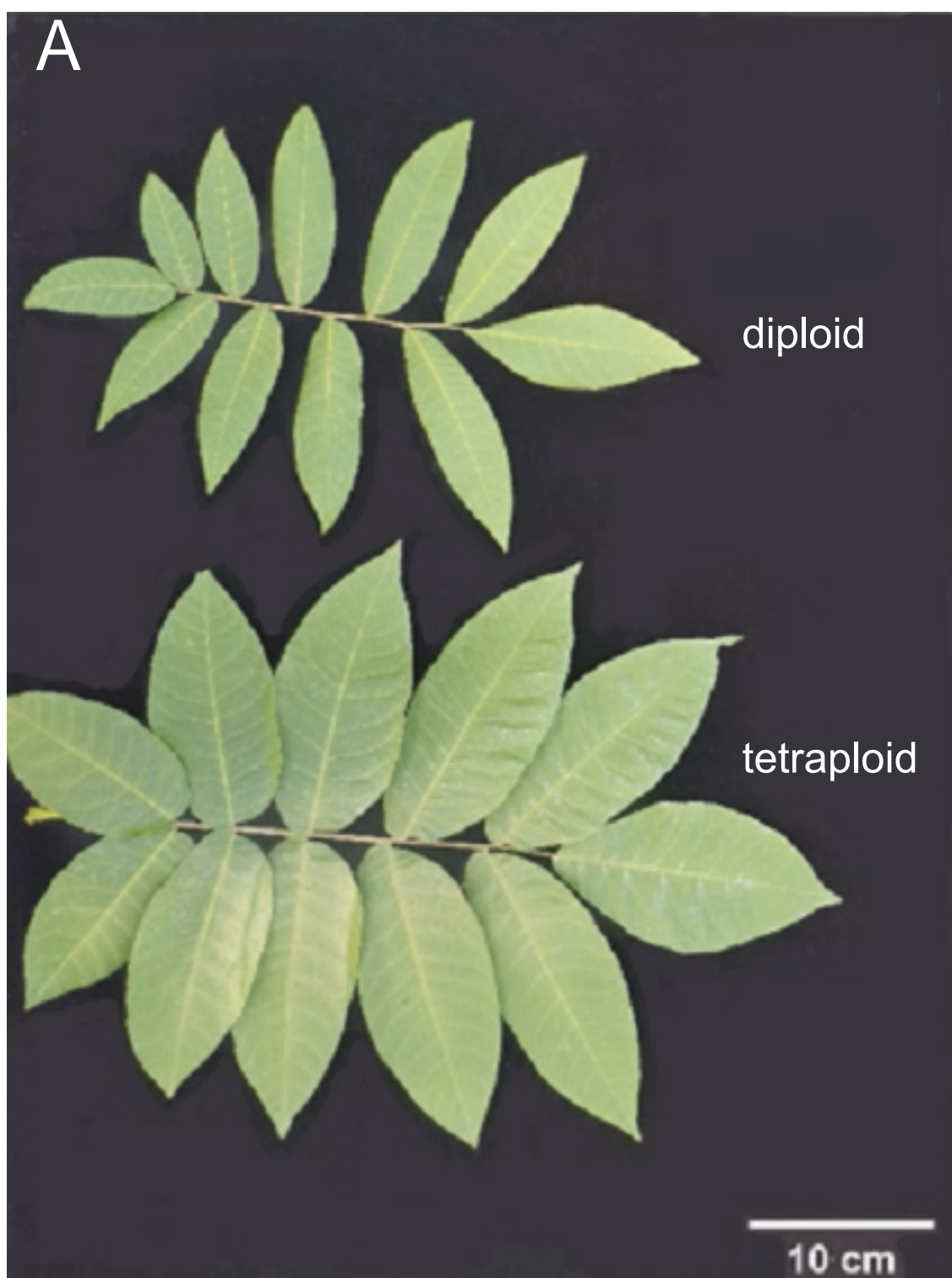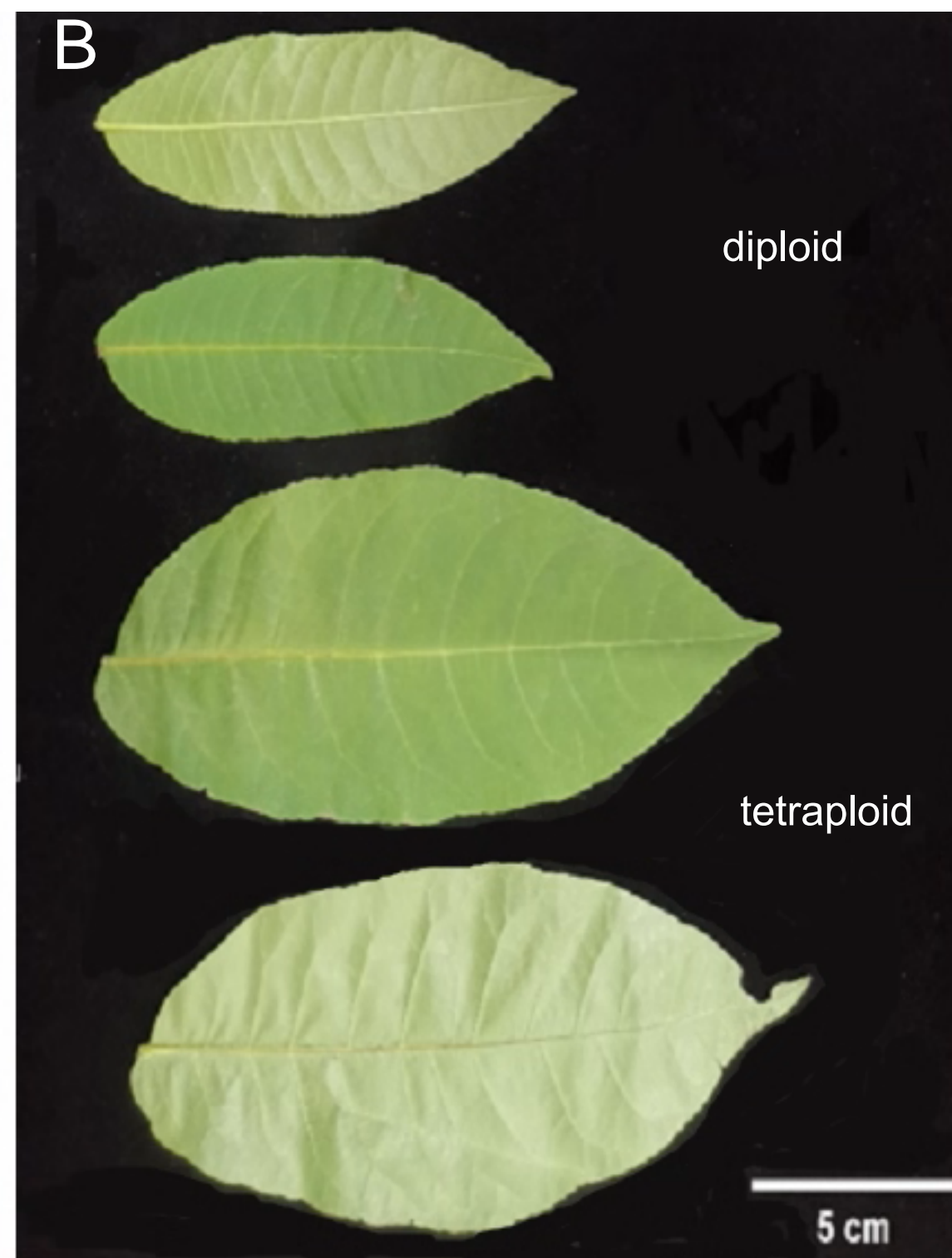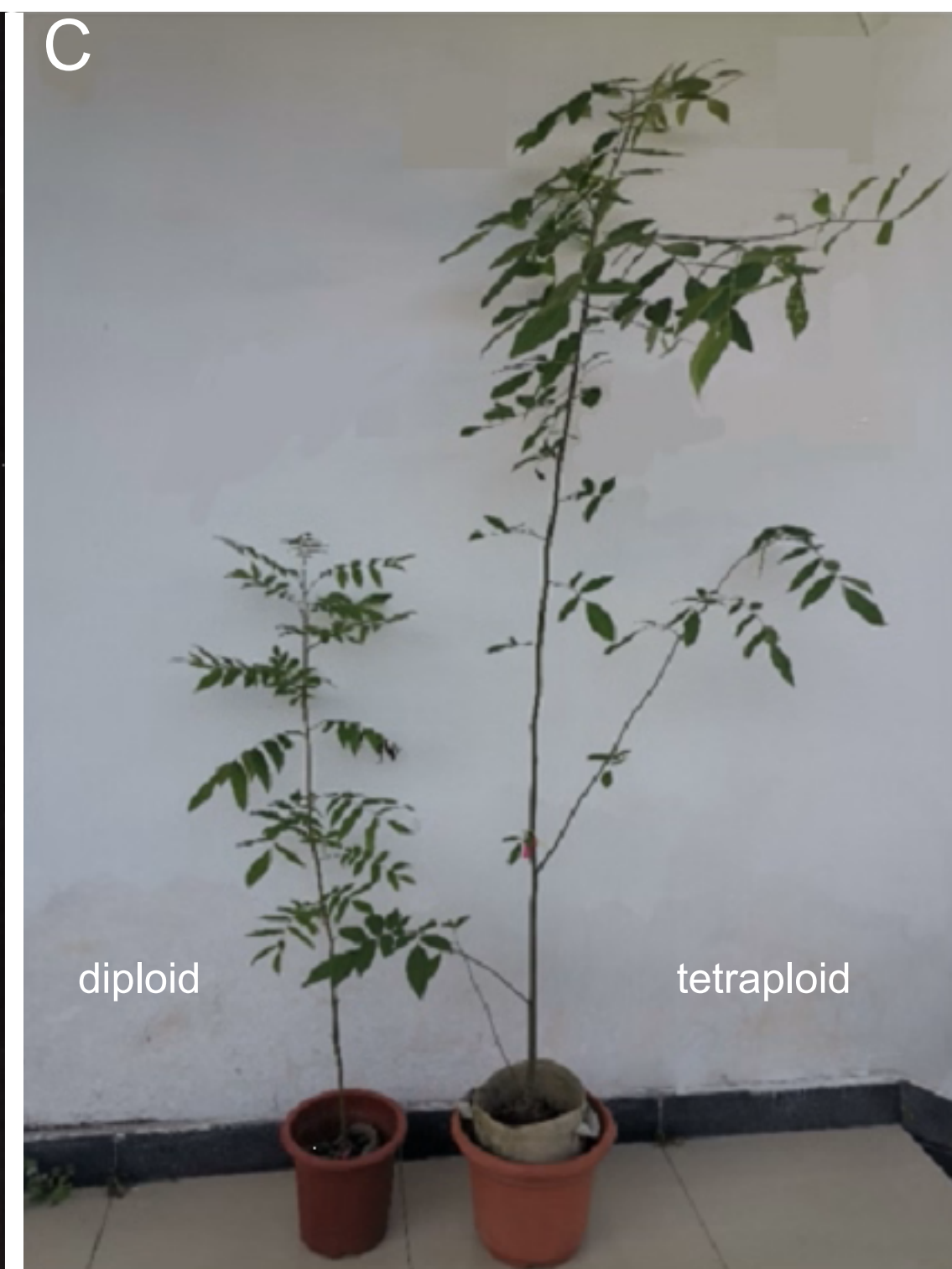

Supplement: Supplementary Figure S29 — The morphological difference between diploid and tetraploid C. paliurus A. Compound leaf. B. Singe leaf. C. Seedlings. [file mmc30.pdf]

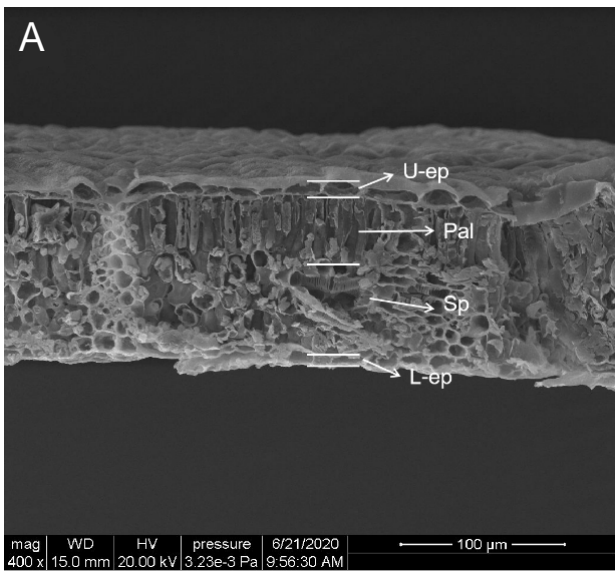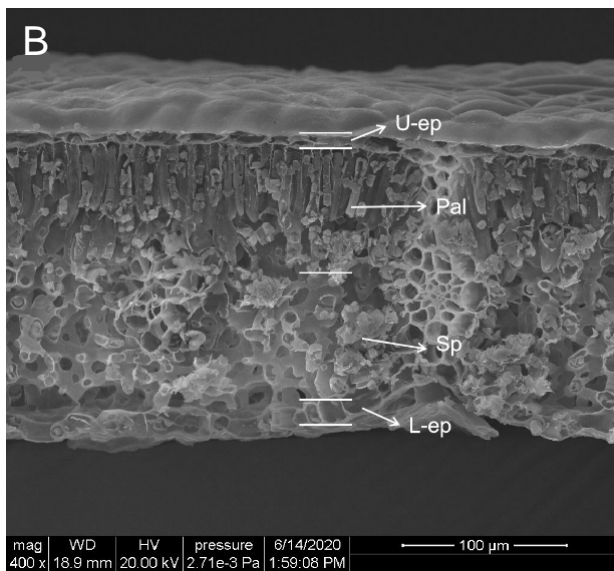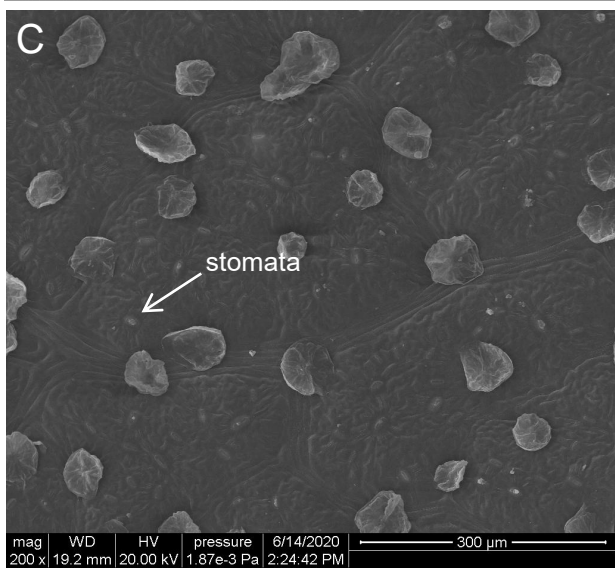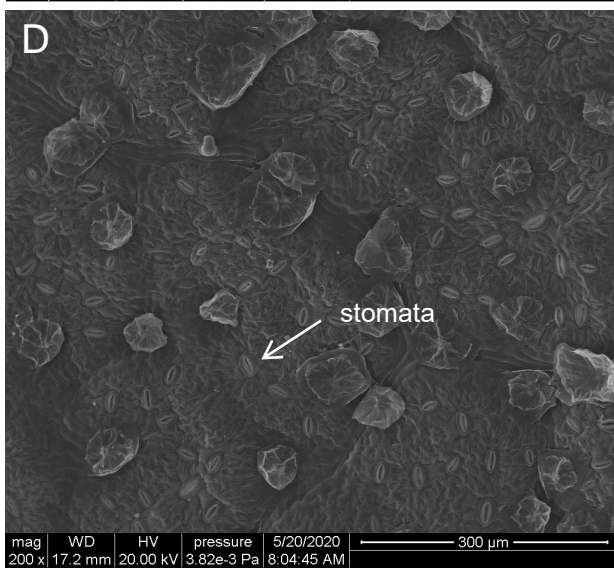

Supplement: Supplementary Figure S30 — The comparison of leaf thickness and stomatal density between diploid and tetraploid C. paliurus based on scanning electron microscopy A. Leaf thickness of diploid sample. B. Leaf thickness of tetraploid sample. C. Stomatal density of diploid sample. D. Stomatal density of tetraploid sample. U-ep, upper epidermal cells; L-ep, lower epidermal cells; Pal, palisade mesophyll; Sp, sponge tissue. [file mmc31.pdf]

**A**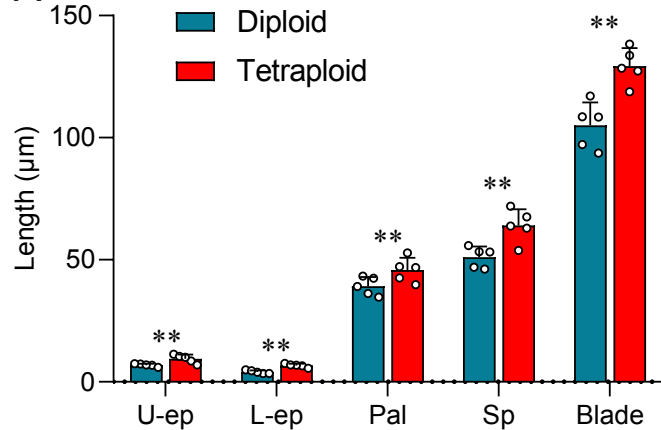**B**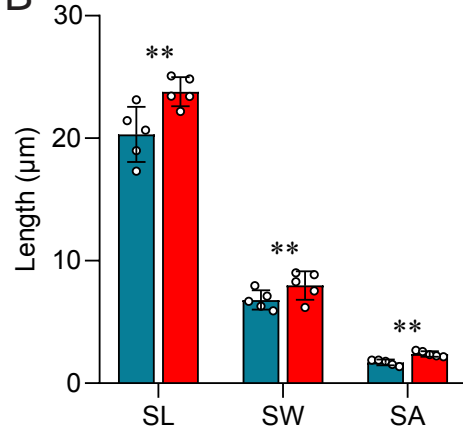**C**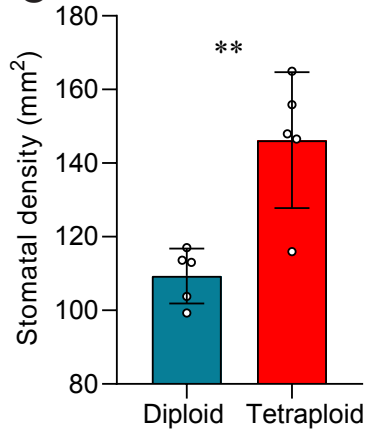

Supplement: Supplementary Figure S31 — Quantification of anatomical structure values in different ploidy C. paliurus A. Thickness of leaf tissues. B. Stomatal size of leaf. C. Stomatal density. **, P value < 0.01. [file mmc32.pdf]

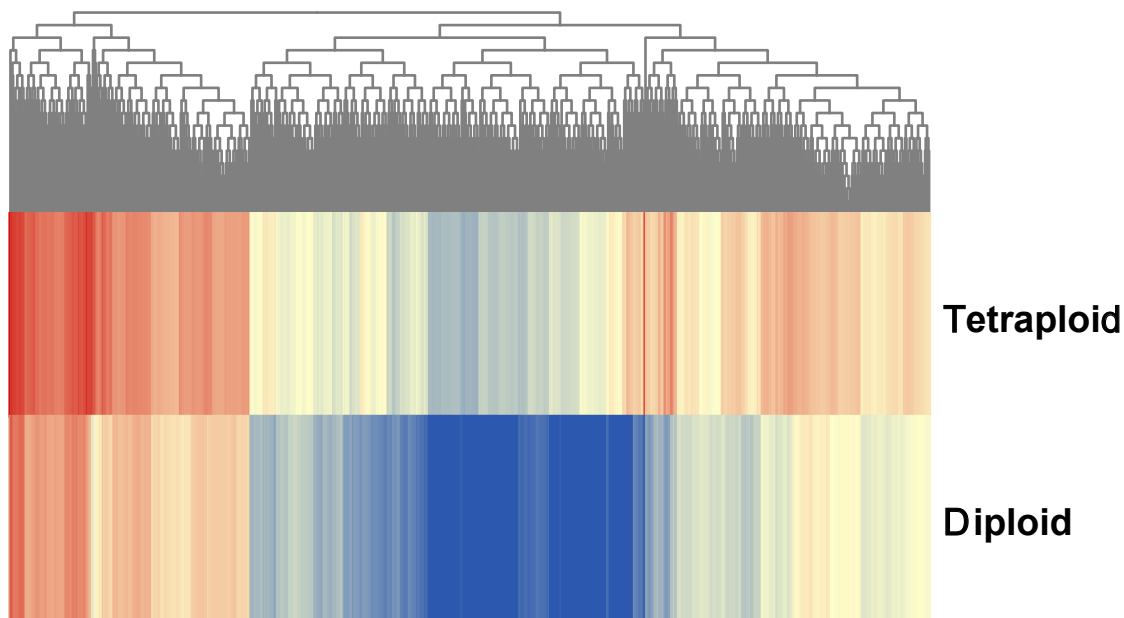

$\text{Log}_2\text{FPKM}$

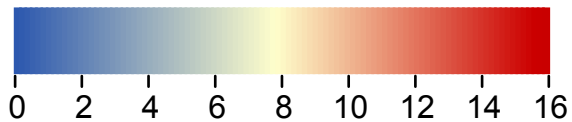

Supplement: Supplementary Figure S32 — Heatmap showing the expression patterns of 691 dosage-effect genes among tetraploid and diploid samples [file mmc33.pdf]

Description

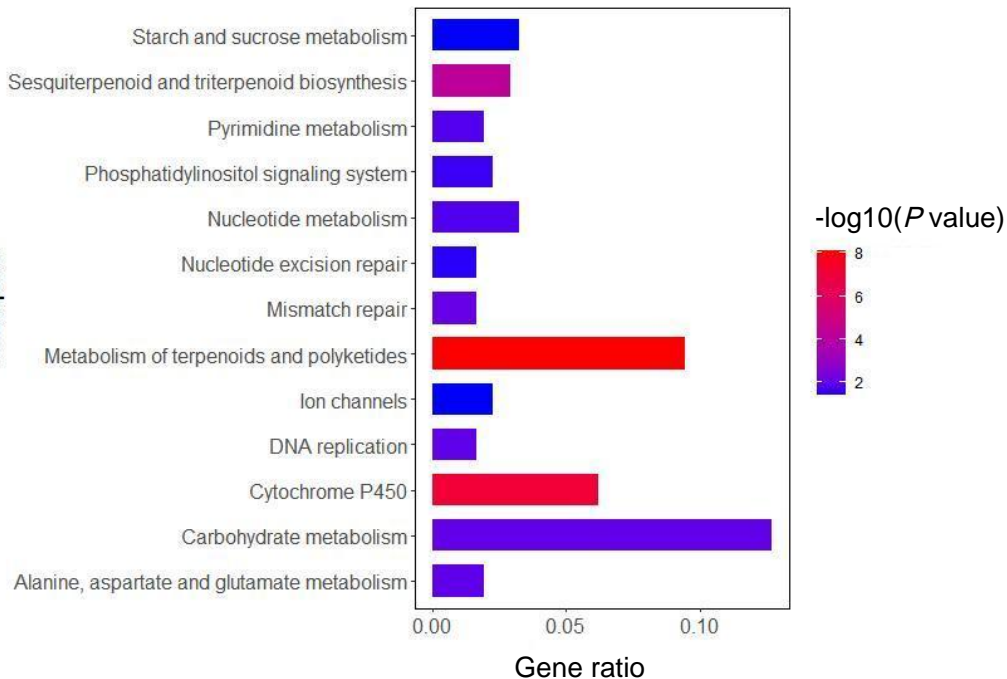

Supplement: Supplementary Figure S33 — KEGG pathway enrichment analysis of up-regulated genes in tetraploid samples [file mmc34.pdf]

GO

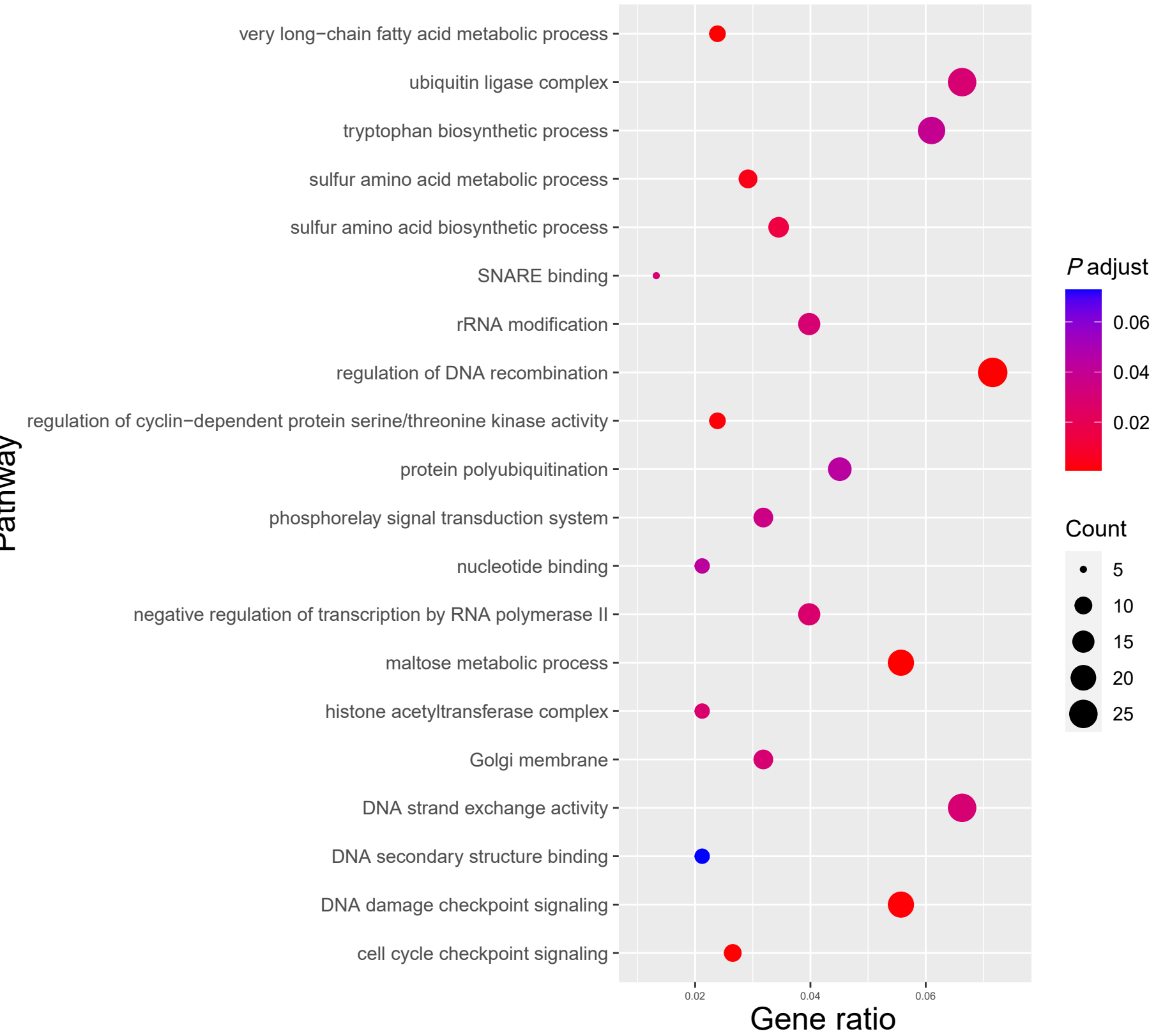

KEGG

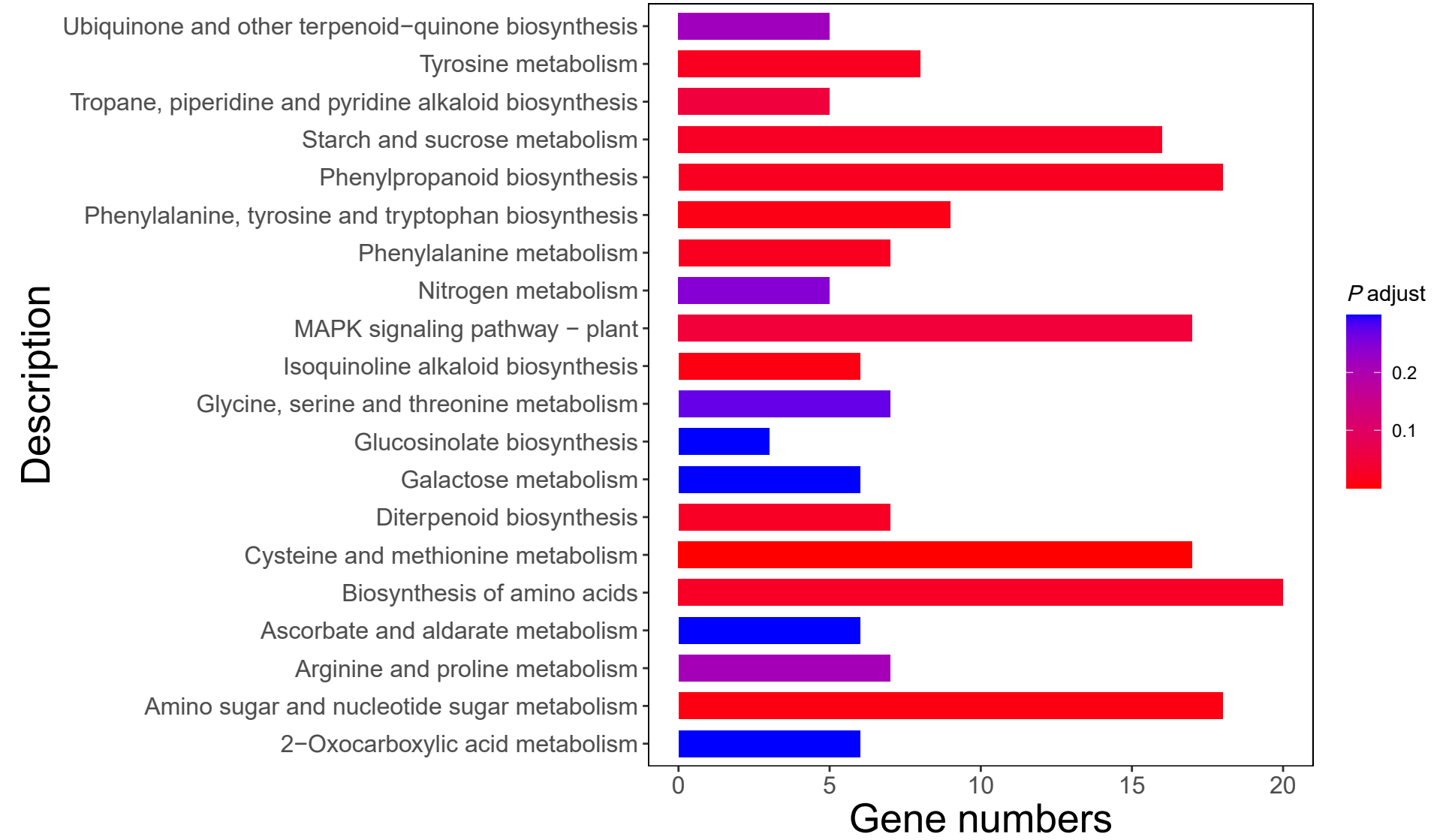

Supplement: Supplementary Figure S35 — Functional enrichment analysis of dosage compensation effect genes inC. paliurus [file mmc36.pdf]

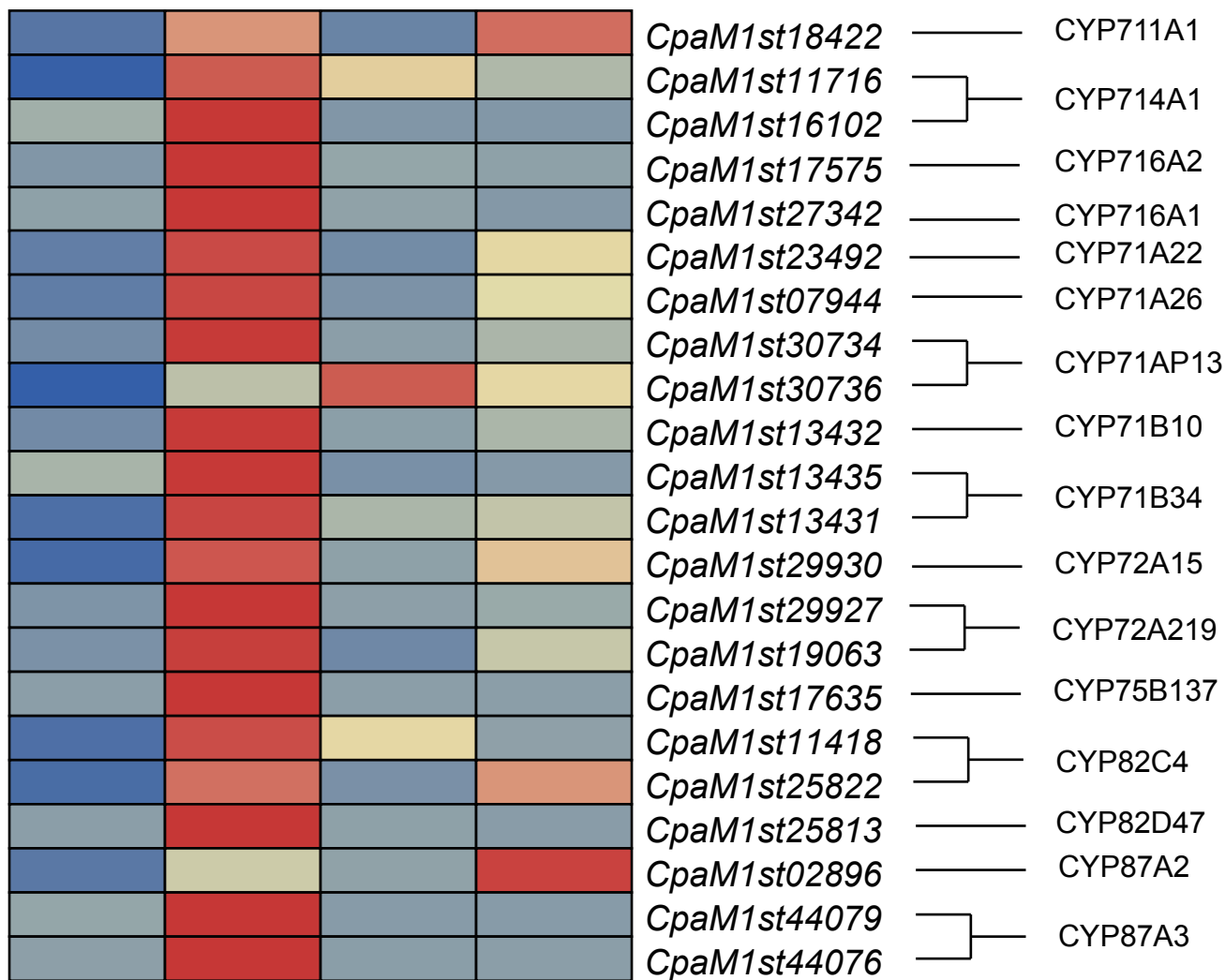

Sept\_diploid

Sept\_tetraploid

May\_diploid

May\_tetraploid

FPKM (Z-score)

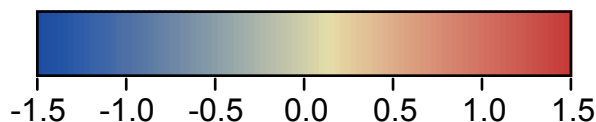

Supplement: Supplementary Figure S36 — Heatmap showing the expression patterns of P450 family genes of dosageeffect genes across four samples [file mmc37.pdf]

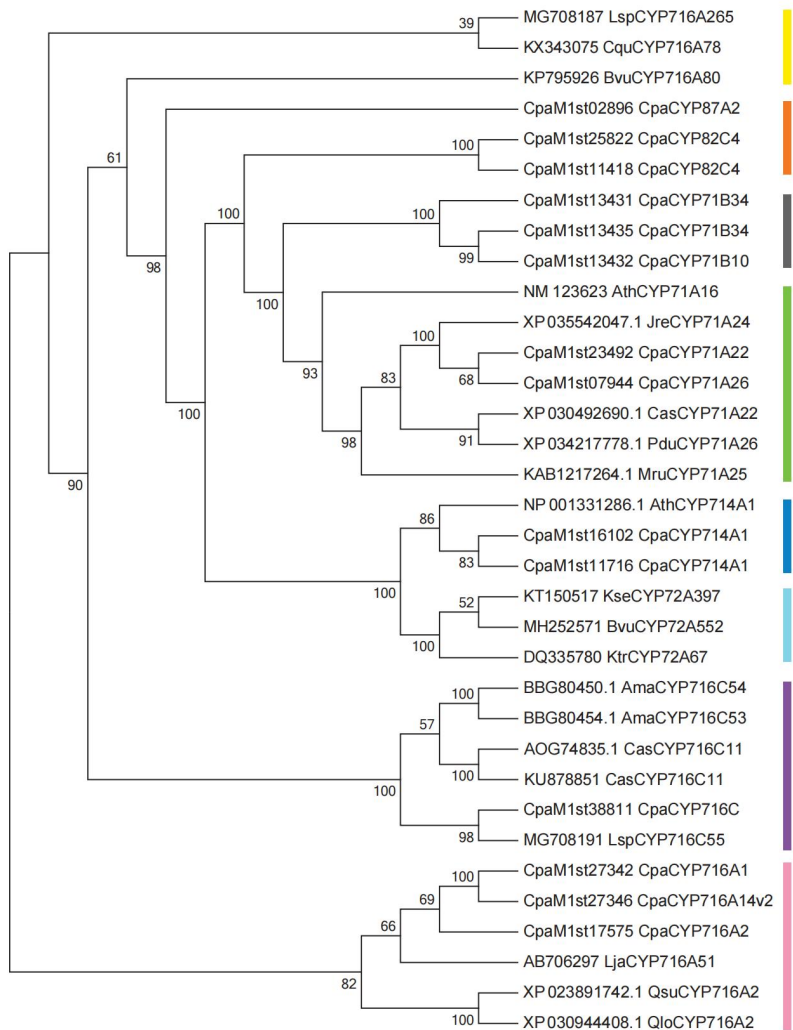

Supplement: Supplementary Figure S37 — Phylogenetic tree of P450 gene family in multiple species, including C. paliurus, Centella asiatica, Avicennia marina, Lagerstroemia speciosa, Moreua rubra, J. regia, Prunus duscis, A. thaliana, Kalopanax truncatula, Barbarea vulgaris, Chenopodium quinoa, Lotus japonicus, Quercus suber, Kalopanax septemlobus, and Quercus lobata [file mmc38.pdf]

A

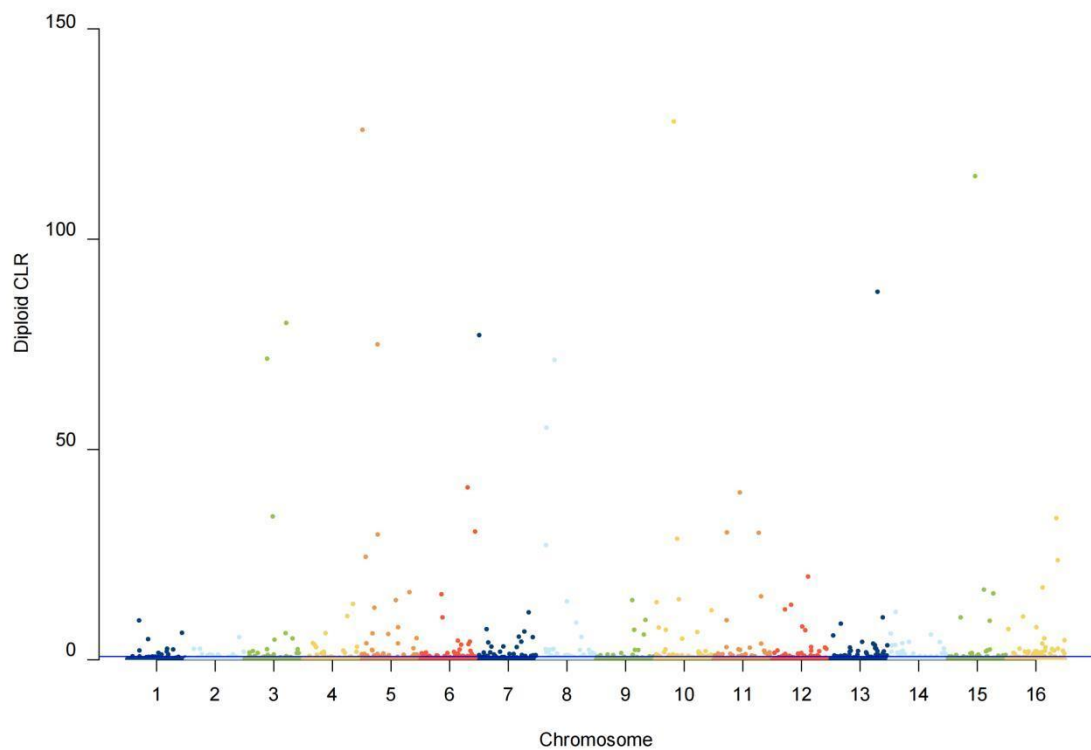

B

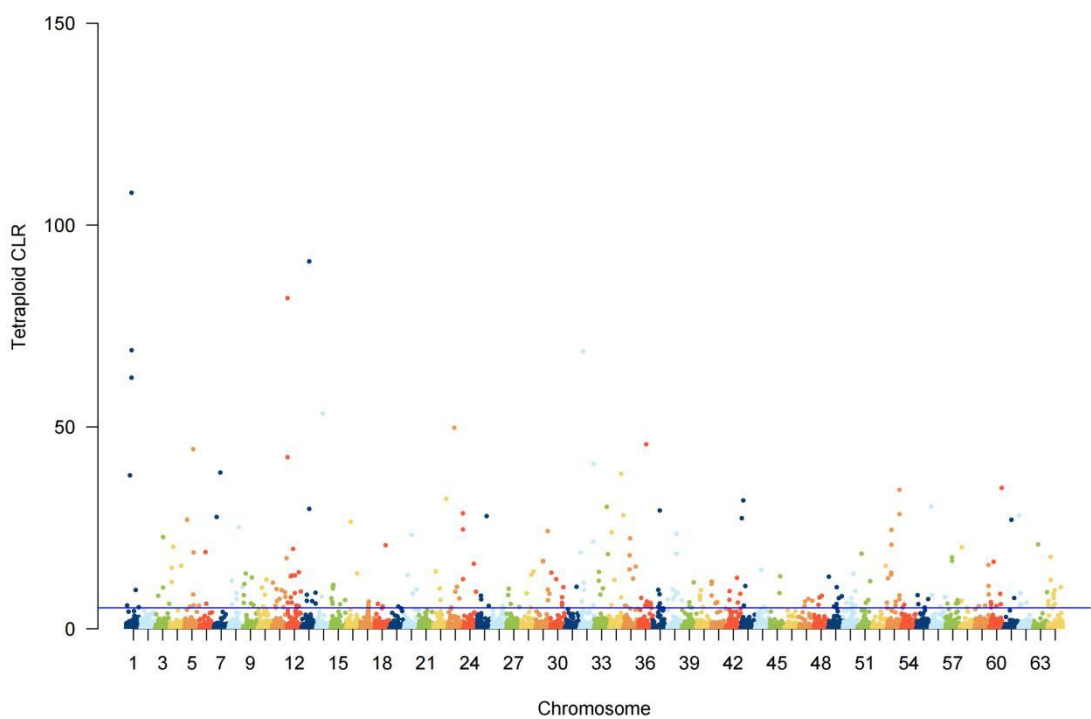

Supplement: Supplementary Figure S38 — Distribution of selective sweep regions in C. paliurus genome A. Diploid. B. Tetraploid. CLR, composite likelihood ratio. [file mmc39.pdf]

# GO enrichment

Pathway

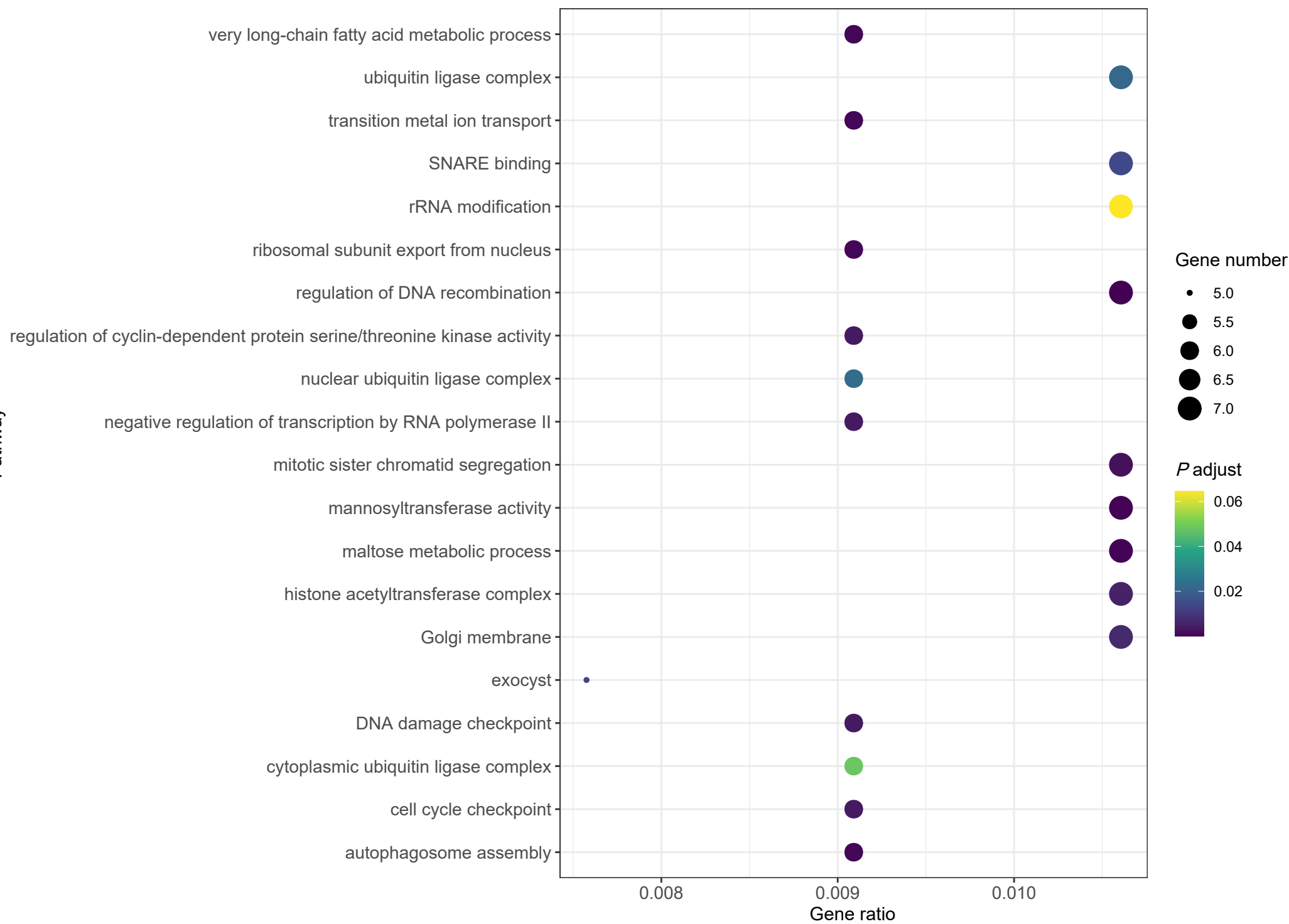

Supplement: Supplementary Figure S39 — GO enrichment analysis of genes under strong selective sweep in diploidC. paliuru [file mmc40.pdf]

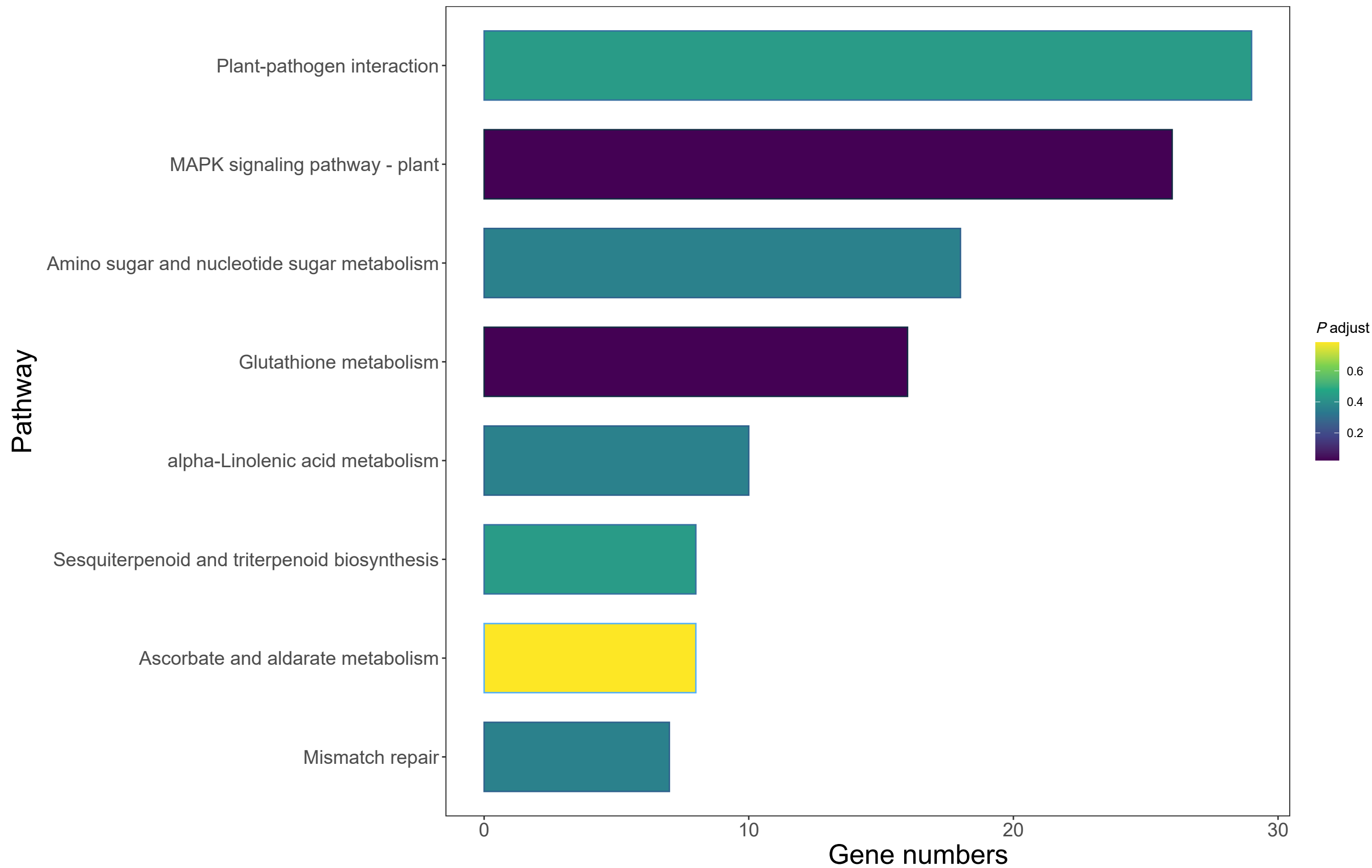

Supplement: Supplementary Figure S40 — KEGG pathway analysis of genes under strong selective sweep in diploid C. paliurus [file mmc41.pdf]

# GO enrichment

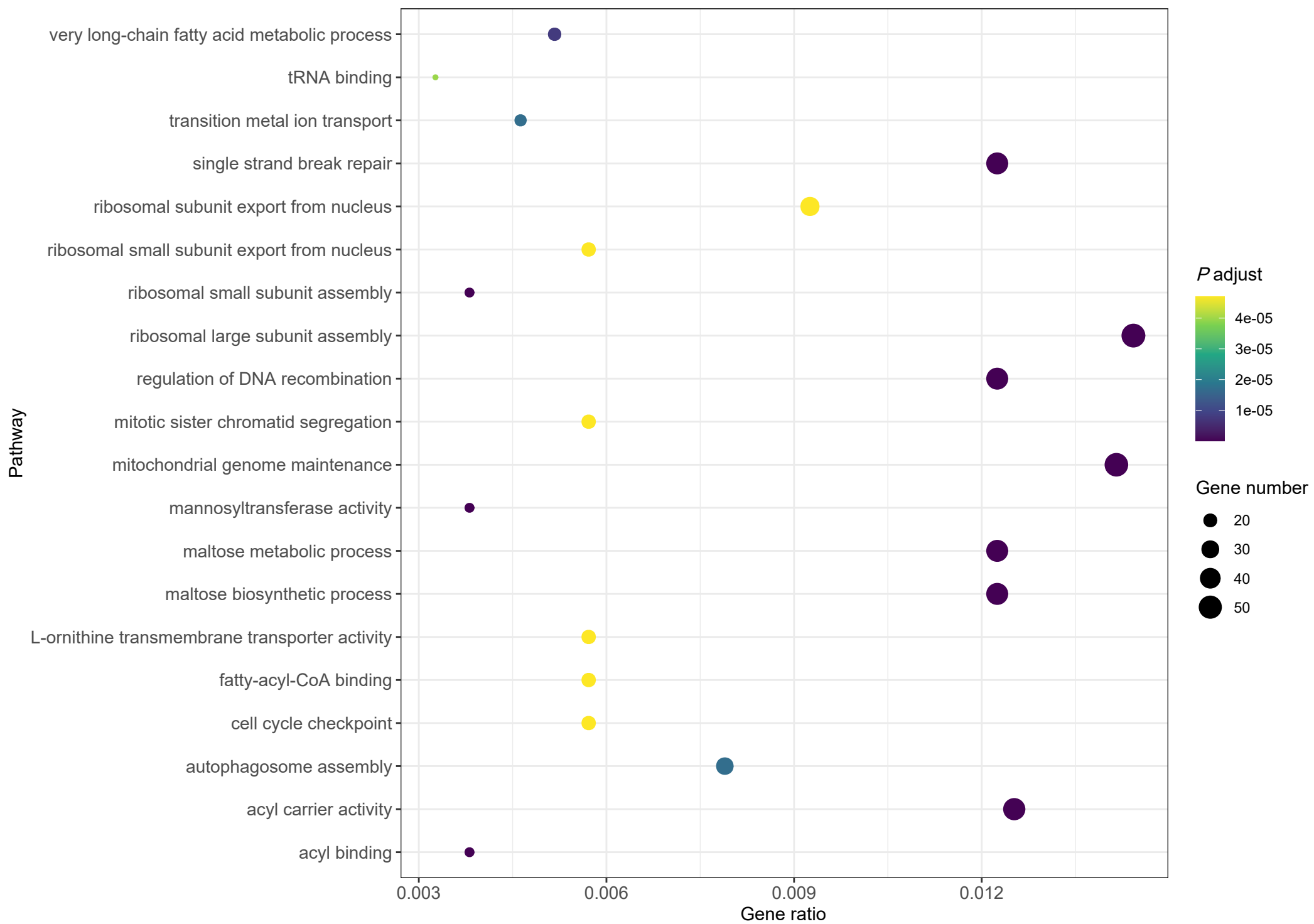

Supplement: Supplementary Figure S41 — GO enrichment analysis of genes under strong selective sweep in tetraploidC. paliurus [file mmc42.pdf]

Pathway

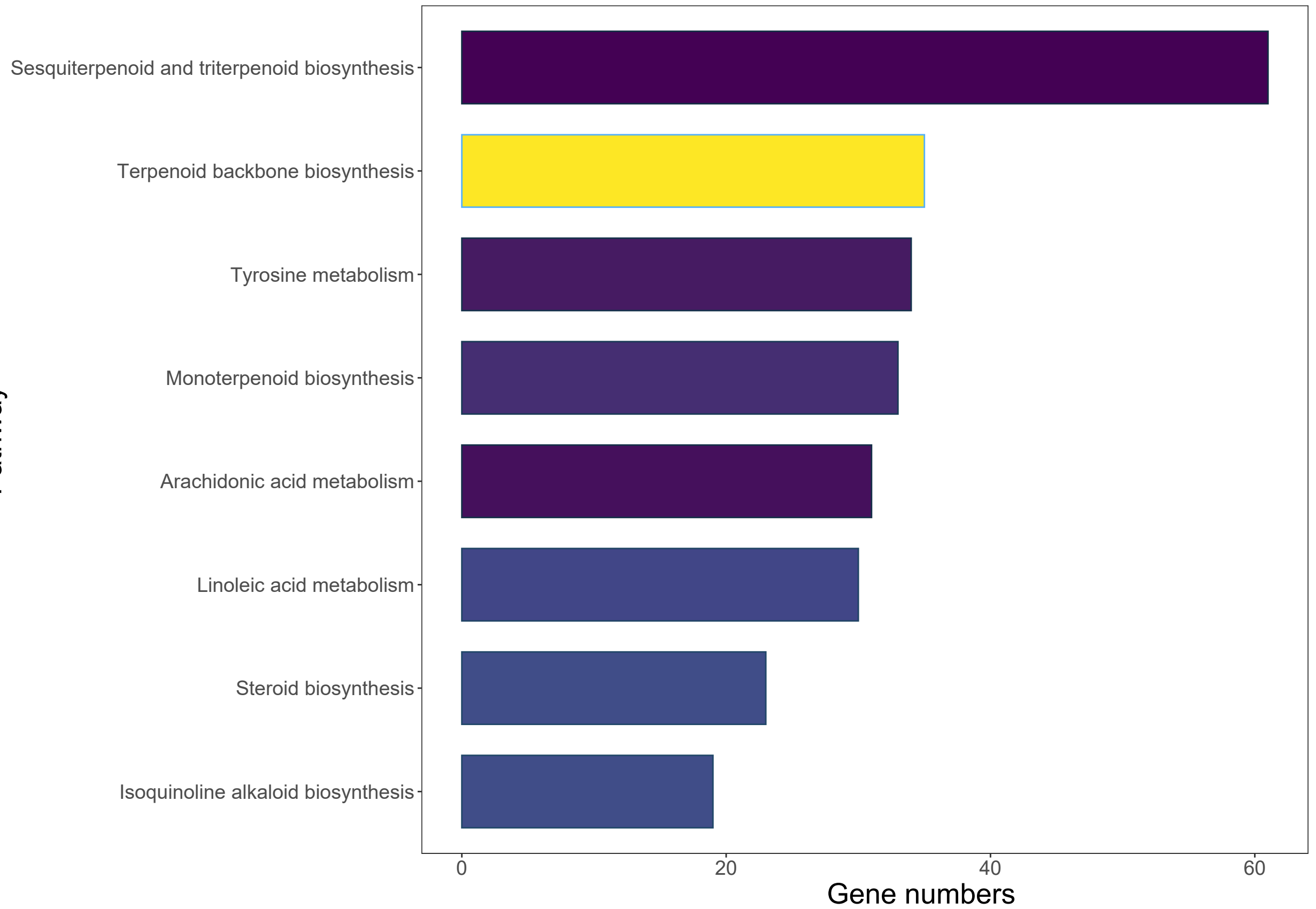

Supplement: Supplementary Figure S42 — KEGG pathway analysis of genes under strong selective sweep in tetraploidC. paliurus [file mmc43.pdf]

Tetraploid

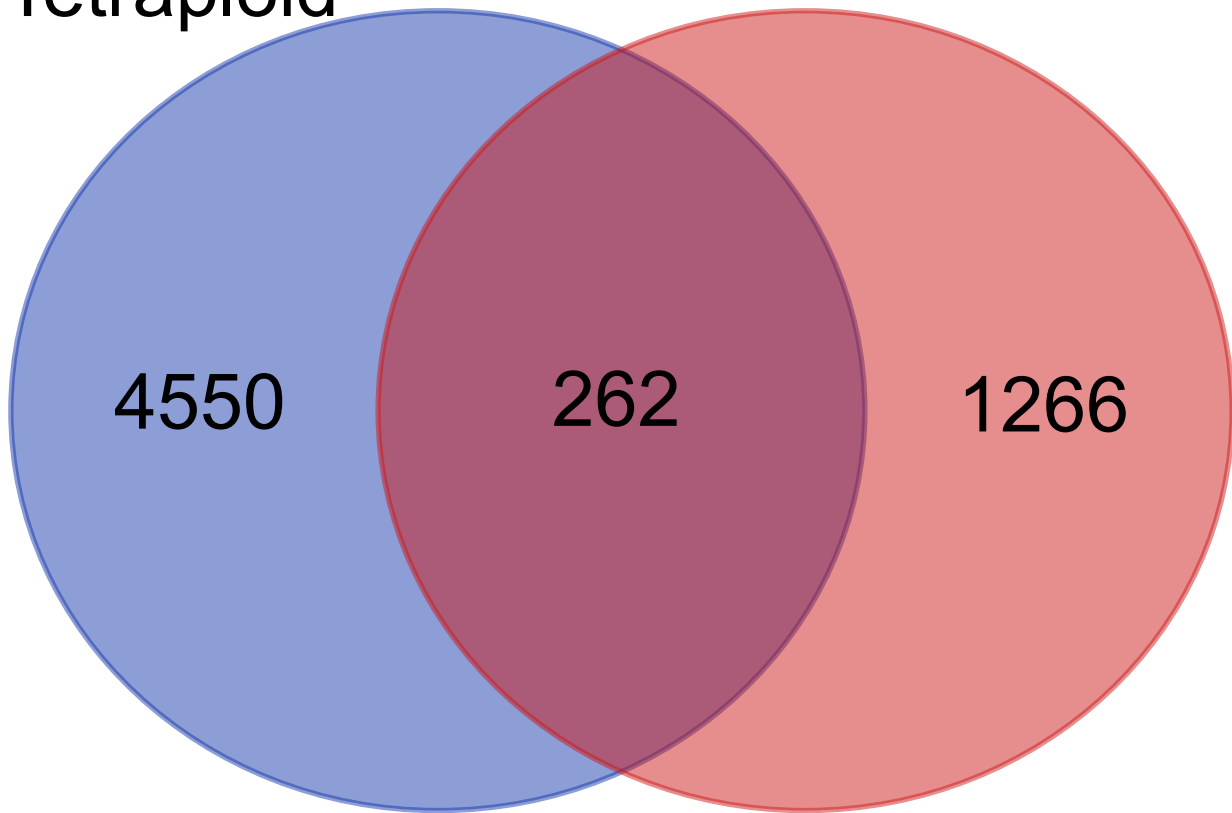

Diploid

Supplement: Supplementary Figure S43 — Venn diagrams of selective genes in diploid and tetraploidC. paliurus [file mmc44.pdf]

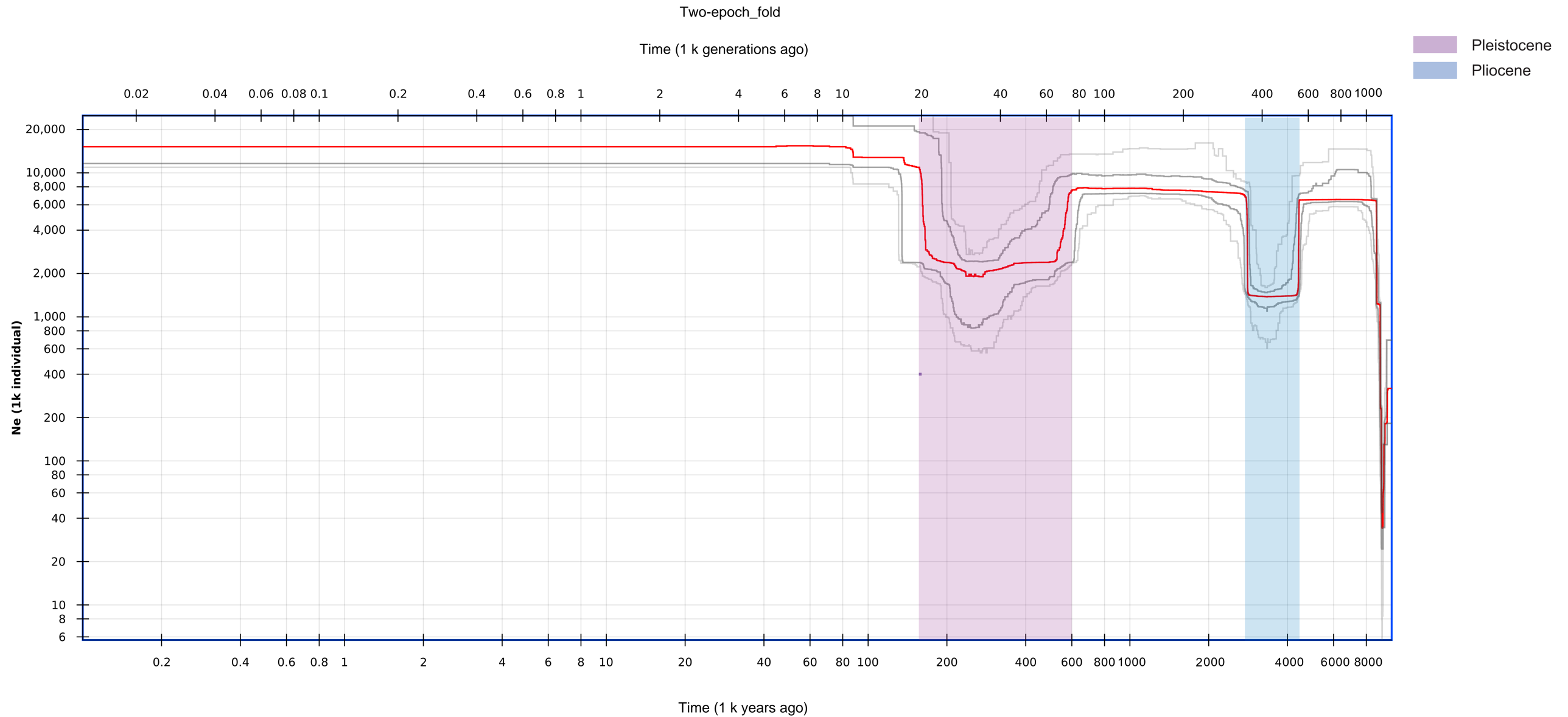

Supplement: Supplementary Figure S45 — Demographic history of C. paliurus Historical effective population size for C. paliurus beginning from 8 million years ago to present. Stairway plot showing that the C. paliurus population has undergone bottlenecks during two known periods of major climate upheaval: the Pleistocene (purple) and the Pliocene (blue). Ne, effective population size. [file mmc46.pdf]

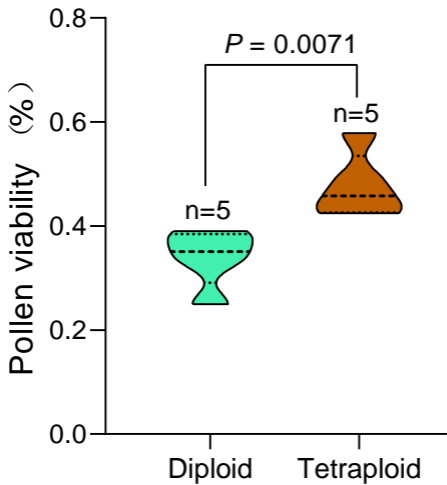

Supplement: Supplementary Figure S46 — The comparison of pollen viability between diploid and tetraploidC. paliurus [file mmc47.pdf]
