# Supplementary material for: Whole-genome Duplication Reshaped Adaptive Evolution in A Relict Plant Species, Cyclocarya paliurus
Source: Genomics Proteomics Bioinformatics. 2023 Feb 11;21(3):455–69. doi: 10.1016/j.gpb.2023.02.001 (PMC10787019; doi:10.1016/j.gpb.2023.02.001)
Supplement: Supplementary Figure S17 — The P450 clusters of expanded genes are arranged on Chr1, Chr4, and Chr12 P450, cytochrome P450 monooxygenase. [file mmc18.pdf]

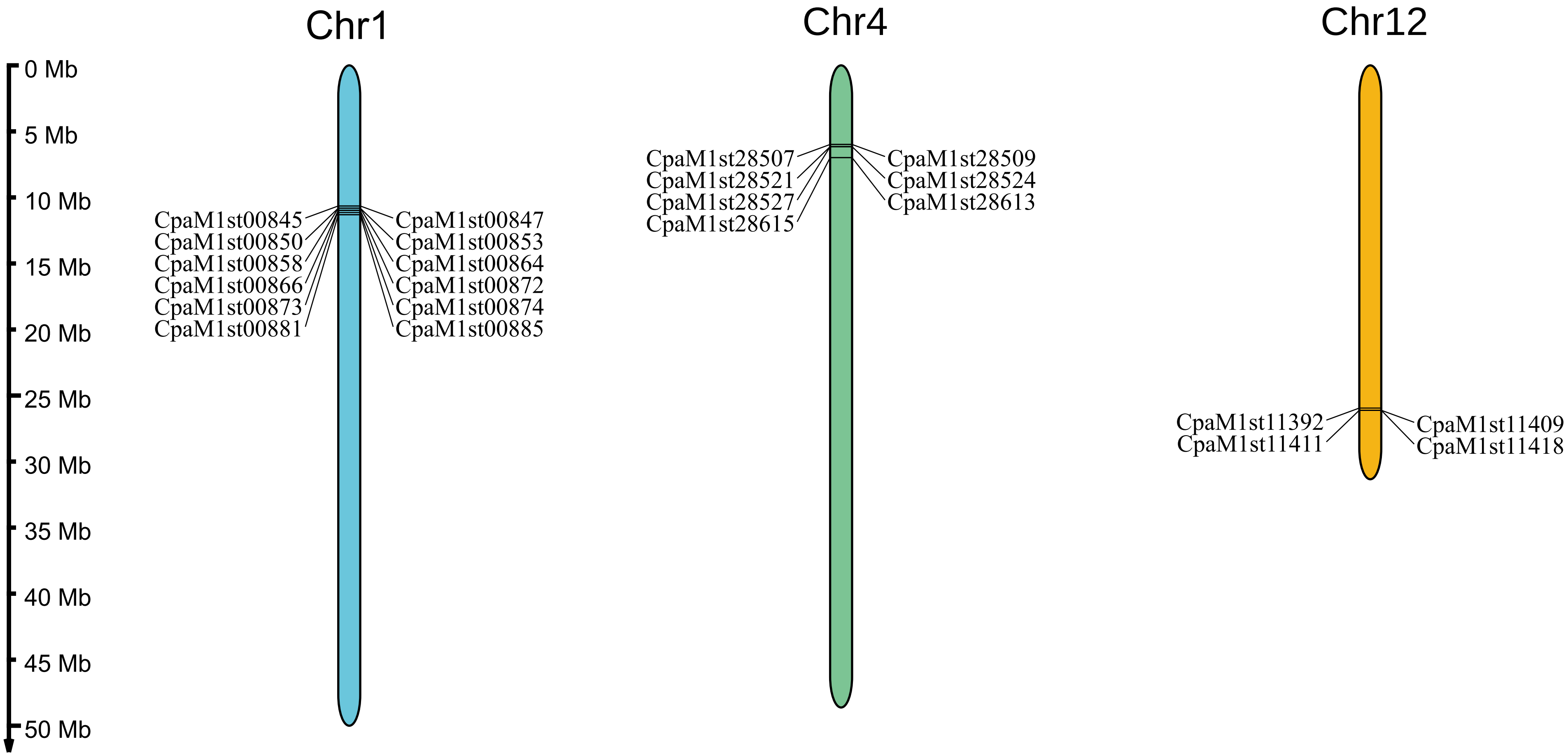
Chr1
Chr4
Chr12
0 Mb
5 Mb
10 Mb
15 Mb
20 Mb
25 Mb
30 Mb
35 Mb
40 Mb
45 Mb
50 Mb
CpaM1st00845
CpaM1st00850
CpaM1st00858
CpaM1st00866
CpaM1st00873
CpaM1st00881
CpaM1st00847
CpaM1st00853
CpaM1st00864
CpaM1st00872
CpaM1st00874
CpaM1st00885
CpaM1st28507
CpaM1st28521
CpaM1st28527
CpaM1st28615
CpaM1st28509
CpaM1st28524
CpaM1st28613
CpaM1st11392
CpaM1st11411
CpaM1st11409
CpaM1st11418
