# Supplementary material for: Whole-genome Duplication Reshaped Adaptive Evolution in A Relict Plant Species, Cyclocarya paliurus
Source: Genomics Proteomics Bioinformatics. 2023 Feb 11;21(3):455–69. doi: 10.1016/j.gpb.2023.02.001 (PMC10787019; doi:10.1016/j.gpb.2023.02.001)
Supplement: Supplementary Figure S19 — Identification and functional enrichment of DEGs between PG and PA C. paliurus at five flowering time stages (S0–S4) A. Venn diagrams of DEGs in the female floral buds (PG-F vs. PA-F). B. Venn diagrams of DEGs in the male floral buds (PA-M vs. PG-M). C. GO enrichment of the 958 DEGs in female floral buds (PG-F vs. PA-F). D. GO enrichment of the 2373 DEGs in male floral buds (PA-M vs. PG-M). In all subsequent figures, PG-F means female floral buds of PG, PA-F means female floral buds of PA, PG-M means male floral buds of PG, and PA-M means male floral buds of PA. FC, fold change; DEGs, differentially expressed genes; PG, protogyny; PA, protandry. [file mmc20.pdf]

A

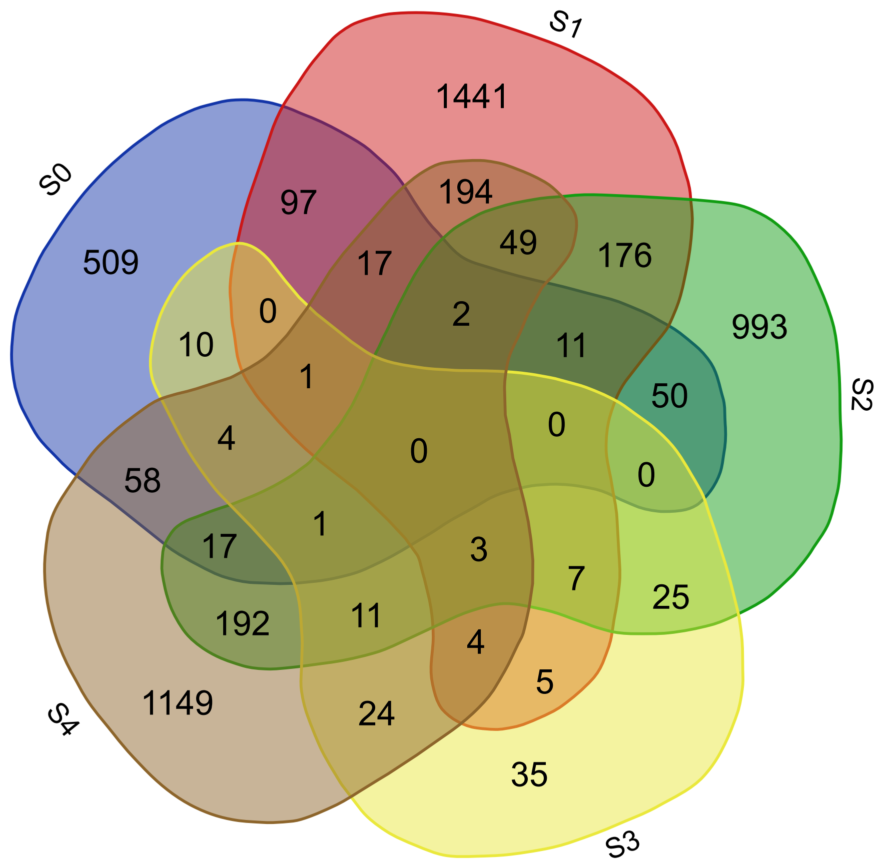

PG-F *vs.* PA-F

C

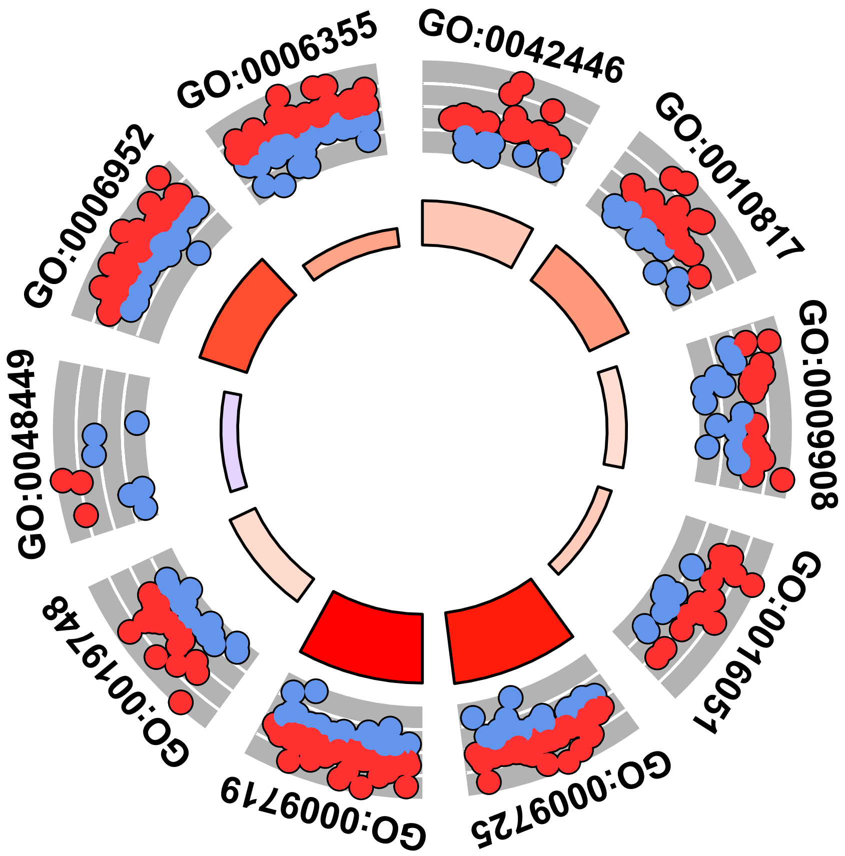

ID	Description
GO:0042446	hormone biosynthetic process
GO:0010817	regulation of hormone levels
GO:0009908	flower development
GO:0016051	carbohydrate biosynthetic process
GO:0009725	response to hormone
GO:0009719	response to endogenous stimulus
GO:0019748	secondary metabolic process
GO:0048449	floral organ formation
GO:0006952	defense response
GO:0006355	regulation of transcription, DNA−templated

z−score
decreasing increasing
logFC
downregulated upregulated

B

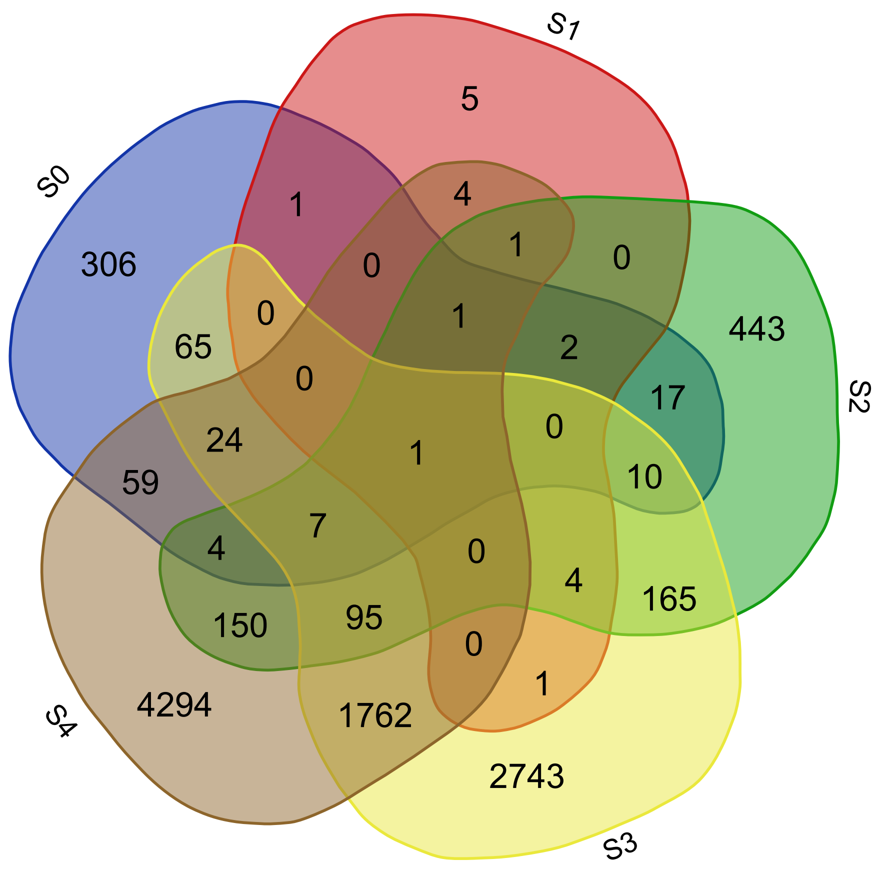

PA-M *vs.* PG-M

D

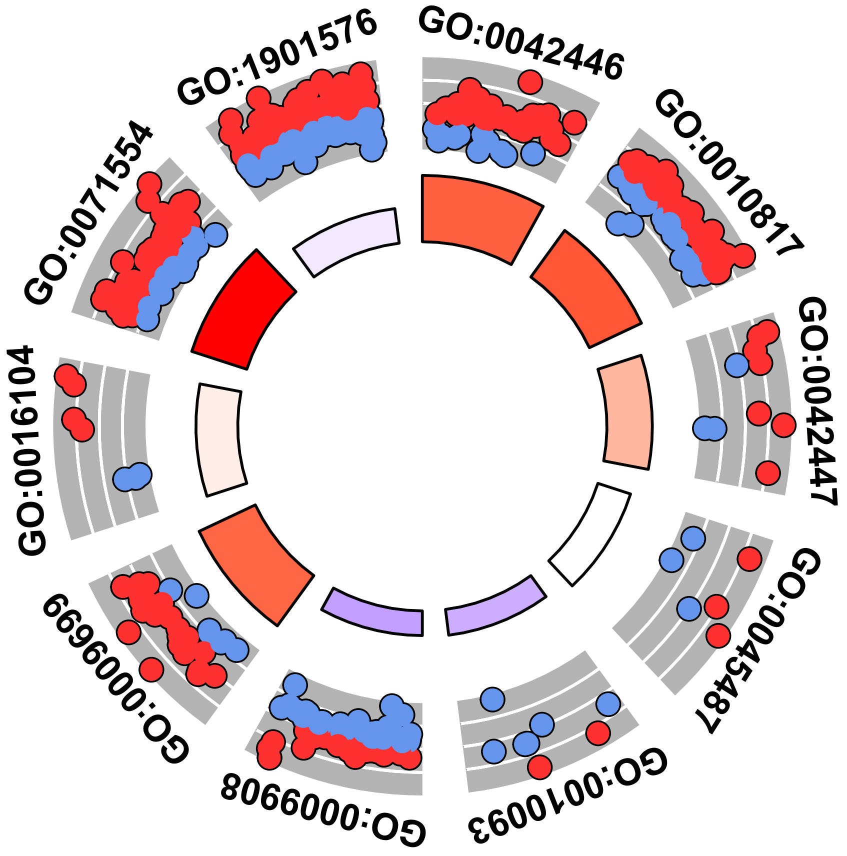

ID	Description
GO:0042446	hormone biosynthetic process
GO:0010817	regulation of hormone levels
GO:0042447	hormone catabolic process
GO:0045487	gibberellin catabolic process
GO:0010093	specification of floral organ identity
GO:0009908	flower development
GO:0009699	phenylpropanoid biosynthetic process
GO:0016104	triterpenoid biosynthetic process
GO:0071554	cell wall organization or biogenesis
GO:1901576	organic substance biosynthetic process

z−score
decreasing increasing
logFC
down-regulated up-regulated
