# Supplementary material for: Whole-genome Duplication Reshaped Adaptive Evolution in A Relict Plant Species, Cyclocarya paliurus
Source: Genomics Proteomics Bioinformatics. 2023 Feb 11;21(3):455–69. doi: 10.1016/j.gpb.2023.02.001 (PMC10787019; doi:10.1016/j.gpb.2023.02.001)
Supplement: Supplementary Figure S34 — Heatmap showing the expression patterns of photosynthesis-related genes CA, carbonic anhydrase; PPCK, phosphoenolpyruvate carboxylase kinase; Rubisco, ribulose-1,5-bisphosphate carboxylase/oxygenase; FBP, fructose-1,6-bisphosphatase; SBPASE, sedoheptulose-1,7-bisphosphatase. [file mmc35.pdf]

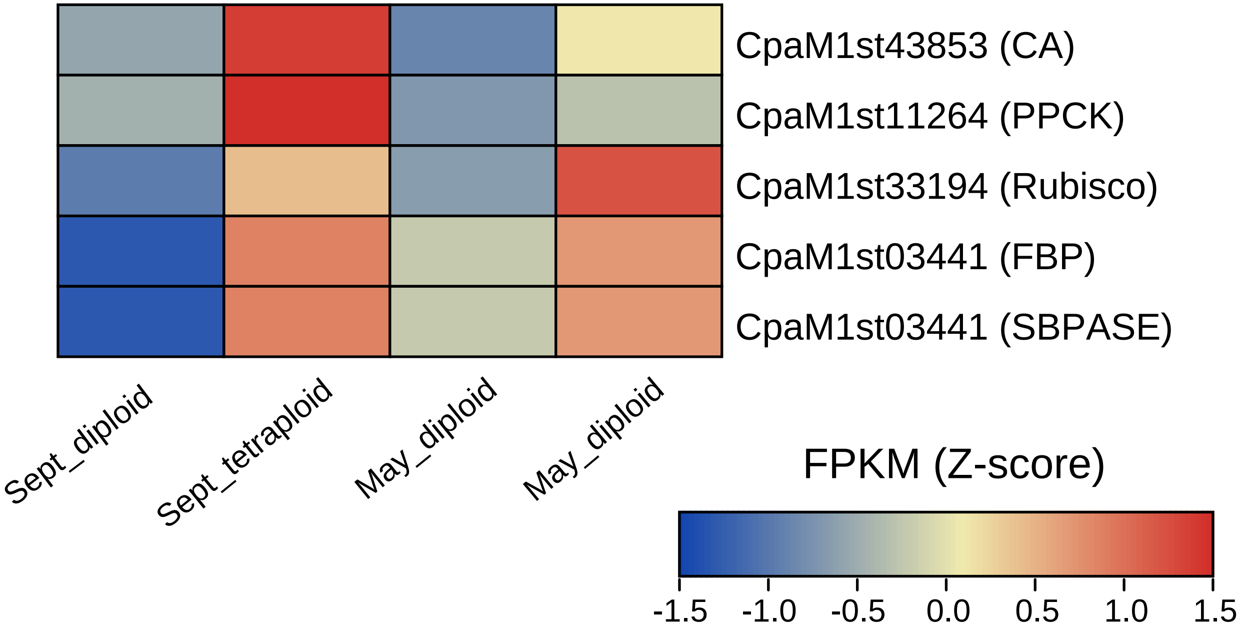
CpaM1st43853 (CA)
CpaM1st11264 (PPCK)
CpaM1st33194 (Rubisco)
CpaM1st03441 (FBP)
CpaM1st03441 (SBPASE)
Sept_diploid
Sept_tetraploid
May_diploid
May_diploid
FPKM (Z-score)
-1.5
-1.0
-0.5
0.0
0.5
1.0
1.5
