# Supplementary material for: Whole-genome Duplication Reshaped Adaptive Evolution in A Relict Plant Species, Cyclocarya paliurus
Source: Genomics Proteomics Bioinformatics. 2023 Feb 11;21(3):455–69. doi: 10.1016/j.gpb.2023.02.001 (PMC10787019; doi:10.1016/j.gpb.2023.02.001)
Supplement: Supplementary Figure S44 — GO and KEGG enrichment of selective genes specific to tetraploidC. paliurus [file mmc45.pdf]

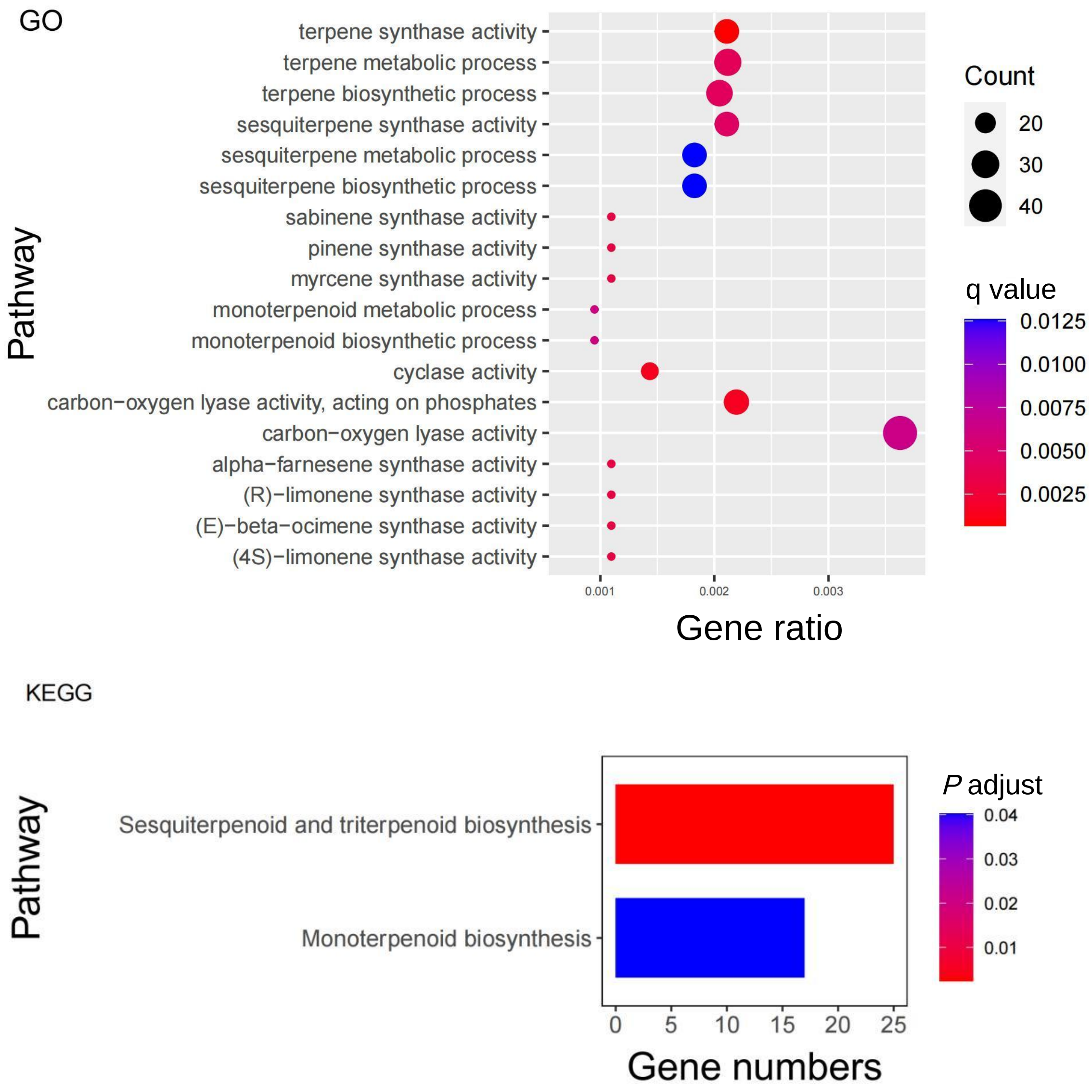

Supplementary Figure 43. GO and KEGG enrichment of selective genes specific to tetraploid *C. paliurus*.
