# Supplementary material for: Whole-genome Duplication Reshaped Adaptive Evolution in A Relict Plant Species, Cyclocarya paliurus
Source: Genomics Proteomics Bioinformatics. 2023 Feb 11;21(3):455–69. doi: 10.1016/j.gpb.2023.02.001 (PMC10787019; doi:10.1016/j.gpb.2023.02.001)
Supplement: Supplementary Table S1 — Sequencing information for the assemblies [file mmc48.docx]

| **Library type** | **Insert size** | **Raw data (Gb)** | | | **Coverage (×)** | | |
| --- | --- | --- | --- | --- | --- | --- | --- |
|  |  | **PA-dip** | **PG-dip** | **PA-tetra** | **PA-dip** | **PG-dip** | **PA-tetra** |
| Illumina paired-end | 200 bp | 106.7 | 86 | 291.7 | 176 | 131 | 237 |
| PacBio Sequel II | 20 kb | 134.9 | 75.5 | 271.8 | 223 | 115 | 221 |
| Hi-C | 200 bp | 65.4 | 68 | 264 | 108 | 103 | 215 |
| Total | - | 307 | 229.5 | 827.5 | 507 | 348 | 673 |

**Table S1 Sequencing information for the assemblies**
