# Supplementary material for: Whole-genome Duplication Reshaped Adaptive Evolution in A Relict Plant Species, Cyclocarya paliurus
Source: Genomics Proteomics Bioinformatics. 2023 Feb 11;21(3):455–69. doi: 10.1016/j.gpb.2023.02.001 (PMC10787019; doi:10.1016/j.gpb.2023.02.001)
Supplement: Supplementary Table S2 — Contig-level assemblies [file mmc49.docx]

|  | **PA-dip** | **PG-dip** | **PA-tetra** |
| --- | --- | --- | --- |
| No. of contigs | 1101 | 921 | 9744 |
| Max length (Mb) | 8.68 | 12.50 | 10.32 |
| Assembly size (Mb) | 586.62 | 583.45 | 2380.95 |
| Contig N90 (bp) | 419,278 | 355,229 | 100,226 |
| Contig N50 (bp) | 1,928,354 | 1,389,753 | 430,910 |
| Average (bp) | 532,809 | 633,498 | 244,350 |
| Total No. of contigs (> 2 kb) | 1082 | 912 | 9719 |

**Table S2 Contig-level assemblies**
