# Supplementary material for: Whole-genome Duplication Reshaped Adaptive Evolution in A Relict Plant Species, Cyclocarya paliurus
Source: Genomics Proteomics Bioinformatics. 2023 Feb 11;21(3):455–69. doi: 10.1016/j.gpb.2023.02.001 (PMC10787019; doi:10.1016/j.gpb.2023.02.001)
Supplement: Supplementary Table S3 — Statistics of Hi-C mapping [file mmc50.docx]

|  | **PG-dip** | **PA-dip** | **PA-tetra** |  |
| --- | --- | --- | --- | --- |
| **Statistics of mapping** | | | |  |
| Clean paired-end reads | 195,501,972 | 190,011,721 | 686,905,160 |  |
| Unmapped paired-end reads | 1,793,734 | 2,033,035 | 1,986,108 |  |
| Unmapped paired-end reads rate (%) | 0.917 | 1.070 | 0.289 |  |
| Paired-end reads with singleton | 25,285,318 | 25,084,739 | 37,399,248 |  |
| Paired-end reads with singleton rate (%) | 12.934 | 13.202 | 5.445 |  |
| Multi mapped paired-end reads | 54,214,489 | 57,828,217 | 541,930,877 |  |
| Multi mapped ratio (%) | 27.731 | 30.434 | 78.894 |  |
| Unique mapped paired-end reads | 114,208,431 | 105,065,730 | 105,588,927 |  |
| Unique mapped ratio (%) | 58.418 | 55.294 | 15.372 |  |
| **Statistics of valid reads** | | | |  |
| Unique mapped paired-end reads | 114,208,431 | 105,065,730 | 105,588,927 |  |
| Dangling end paired-end reads | 1,375,950 | 1,597,284 | 8,185,832 |  |
| Dangling end rate (%) | 1.205 | 1.520 | 7.753 |  |
| Self-circle paired-end reads | 125,793 | 94,131 | 87,361 |  |
| Self-circle rate (%) | 0.110 | 0.090 | 0.083 |  |
| Dumped paired-end reads | 9,339,965 | 8,391,600 | 10,003,495 |  |
| Dumped rate (%) | 8.178 | 7.987 | 9.474 |  |
| Interaction paired-end reads | 101,609,002 | 92,261,311 | 85,094,810 |  |
| Interaction rate (%) | 88.968 | 87.813 | 80.591 |  |
| Lib valid paired-end reads | 94,294,785 | 85,581,476 | 77,436,384 |  |
| Lib valid rate (%) | 82.564 | 81.455 | 73.338 |  |
| Lib dup (%) | 17.436 | 18.545 | 26.662 |  |

**Table S3 Statistics of Hi-C mapping**
