# Supplementary material for: Whole-genome Duplication Reshaped Adaptive Evolution in A Relict Plant Species, Cyclocarya paliurus
Source: Genomics Proteomics Bioinformatics. 2023 Feb 11;21(3):455–69. doi: 10.1016/j.gpb.2023.02.001 (PMC10787019; doi:10.1016/j.gpb.2023.02.001)
Supplement: Supplementary Table S4 — Chromosome-level genome assemblies [file mmc51.docx]

**Table S4 Chromosome-level genome assemblies**

|  | **PA-dip (Mb)** | **PG-dip (Mb)** | **PA-tetra (Mb)** | | | |
| --- | --- | --- | --- | --- | --- | --- |
| **Chr ID** |  |  | **Hap A** | **Hap B** | **Hap C** | **Hap D** |
| Chr1 | 46.29 | 46.58 | 41.27 | 38.01 | 42.45 | 50.08 |
| Chr2 | 43.74 | 44.40 | 43.92 | 43.74 | 32.86 | 50.40 |
| Chr3 | 39.60 | 41.58 | 41.77 | 41.49 | 38.96 | 38.16 |
| Chr4 | 38.01 | 38.67 | 39.44 | 42.99 | 36.33 | 35.67 |
| Chr5 | 37.99 | 37.97 | 37.28 | 39.01 | 39.72 | 33.65 |
| Chr6 | 36.49 | 37.30 | 37.88 | 37.41 | 35.25 | 35.28 |
| Chr7 | 36.56 | 37.08 | 44.92 | 35.67 | 40.88 | 25.69 |
| Chr8 | 32.70 | 35.15 | 42.35 | 36.54 | 32.56 | 34.12 |
| Chr9 | 33.84 | 34.68 | 33.98 | 31.55 | 34.36 | 34.52 |
| Chr10 | 32.37 | 32.69 | 31.48 | 32.99 | 31.72 | 30.89 |
| Chr11 | 33.71 | 32.21 | 33.96 | 38.48 | 36.88 | 23.04 |
| Chr12 | 29.00 | 30.21 | 32.31 | 29.82 | 23.49 | 31.52 |
| Chr13 | 28.95 | 29.25 | 27.51 | 29.46 | 35.23 | 22.26 |
| Chr14 | 25.93 | 27.22 | 27.58 | 27.05 | 26.89 | 26.16 |
| Chr15 | 24.52 | 24.85 | 25.30 | 24.02 | 25.61 | 24.33 |
| Chr16 | 23.82 | 24.03 | 27.91 | 20.92 | 23.21 | 18.46 |
| Total length of contigs (Mb) | 586.62 | 583.45 | 2380.95 | | | |
| Total length of chromosome level assembly (Mb) | 543.53 | 553.87 | 2168.65 | | | |
| Anchor rate (%) | 92.65 | 94.93 | 91.08 | | | |

*Note*: Chr, chromosome; ID, identity document; Hap, haplotype.
