# Supplementary material for: Whole-genome Duplication Reshaped Adaptive Evolution in A Relict Plant Species, Cyclocarya paliurus
Source: Genomics Proteomics Bioinformatics. 2023 Feb 11;21(3):455–69. doi: 10.1016/j.gpb.2023.02.001 (PMC10787019; doi:10.1016/j.gpb.2023.02.001)
Supplement: Supplementary Table S6 — BUSCO assessment of genome assemblies [file mmc53.docx]

| **Description** | **PA-dip** | | **PG-dip** | | **PA-tetra** | |
| --- | --- | --- | --- | --- | --- | --- |
|  | **Number** | **Percentage(%)** | **Number** | **Percentage(%)** | **Number** | **Percentage(%)** |
| Complete BUSCOs (C) | 1308 | 95.2 | 1326 | 96.4 | 1312 | 95.5 |
| Complete and single-copy BUSCOs (S) | 1178 | 85.7 | 1194 | 86.8 | 181 | 13.2 |
| Complete and duplicated BUSCOs (D) | 130 | 9.5 | 132 | 9.6 | 1131 | 82.3 |
| Fragmented BUSCOs (F) | 14 | 1.0 | 9 | 0.7 | 8 | 0.6 |
| Missing BUSCOs (M) | 53 | 3.8 | 40 | 2.9 | 55 | 3.9 |
| Total BUSCO groups searched | 1375 | 100 | 1375 | 100 | 1375 | 100 |

**Table S6 BUSCO assessment of genome assemblies**
