# Supplementary material for: Whole-genome Duplication Reshaped Adaptive Evolution in A Relict Plant Species, Cyclocarya paliurus
Source: Genomics Proteomics Bioinformatics. 2023 Feb 11;21(3):455–69. doi: 10.1016/j.gpb.2023.02.001 (PMC10787019; doi:10.1016/j.gpb.2023.02.001)
Supplement: Supplementary Table S7 — Statistics of Illumina short reads remapped to the assemblies [file mmc54.docx]

|  | **Numbers** | | | **Percentage** | | |
| --- | --- | --- | --- | --- | --- | --- |
|  | **PA** | **PG** | **PA-tetra** | **PA** | **PG** | **PA-tetra** |
| Total reads | 315,235,824 | 313,292,807 | 1,321,149,523 | 100.00% | 100.00% | 100.00% |
| Mapped reads | 311,747,061 | 310,862,209 | 1,314,624,201 | 98.89% | 99.22% | 99.51% |
| Paired reads | 311,704,982 | 311,704,982 | 1,318,071,852 | 98.88% | 99.49% | 99.77% |
| Properly paired | 292,135,204 | 299,878,500 | 1,278,400,172 | 93.72% | 96.21% | 96.99% |
| Unmapped reads | 1,073,915 | 667,698 | 1,242,784 | 0.34% | 0.21% | 0.09% |

**Table S7 Statistics of Illumina short reads remapped to the assemblies**
