# Supplementary material for: Whole-genome Duplication Reshaped Adaptive Evolution in A Relict Plant Species, Cyclocarya paliurus
Source: Genomics Proteomics Bioinformatics. 2023 Feb 11;21(3):455–69. doi: 10.1016/j.gpb.2023.02.001 (PMC10787019; doi:10.1016/j.gpb.2023.02.001)
Supplement: Supplementary Table S8 — Allele annotation in auto-tetraploidC. paliurus genome [file mmc55.docx]

|  | **Total no. of genes** | **No. of genes with 4 alleles** | **No. of genes with 3 alleles** | **No. of genes with 2 alleles** | **No. of genes with 1 allele** |
| --- | --- | --- | --- | --- | --- |
| Chr1 | 2777 | 608 | 913 | 669 | 587 |
| Chr2 | 2851 | 639 | 924 | 748 | 540 |
| Chr3 | 2035 | 608 | 486 | 415 | 526 |
| Chr4 | 2349 | 639 | 743 | 531 | 436 |
| Chr5 | 2598 | 548 | 913 | 642 | 495 |
| Chr6 | 2277 | 612 | 717 | 489 | 459 |
| Chr7 | 2364 | 848 | 705 | 430 | 381 |
| Chr8 | 2229 | 372 | 595 | 543 | 719 |
| Chr9 | 2093 | 639 | 608 | 426 | 420 |
| Chr10 | 2210 | 769 | 619 | 395 | 427 |
| Chr11 | 2405 | 623 | 589 | 446 | 747 |
| Chr12 | 1912 | 471 | 699 | 381 | 361 |
| Chr13 | 1924 | 470 | 632 | 453 | 369 |
| Chr14 | 1663 | 591 | 354 | 323 | 395 |
| Chr15 | 1426 | 468 | 346 | 276 | 336 |
| Chr16 | 1520 | 457 | 419 | 342 | 302 |
| Gene with annotated alleles | 34,633 | 9362 | 10,262 | 7509 | 7500 |
| Unanchored genes/alleles | 588 | **-** | **-** | **-** | **-** |

**Table S8 Allele annotation in auto-tetraploid *C*. *paliurus* genome**
