# Supplementary material for: Whole-genome Duplication Reshaped Adaptive Evolution in A Relict Plant Species, Cyclocarya paliurus
Source: Genomics Proteomics Bioinformatics. 2023 Feb 11;21(3):455–69. doi: 10.1016/j.gpb.2023.02.001 (PMC10787019; doi:10.1016/j.gpb.2023.02.001)
Supplement: Supplementary Table S9 — Genes number of the threeC. paliurus genomes [file mmc56.docx]

| **Chromosome number** | **PA-dip** | **PG-dip** | **PA-tetra** | | | | | | | |
| --- | --- | --- | --- | --- | --- | --- | --- | --- | --- | --- |
|  |  |  | **Gene number** | **No. of alleles in Hap A** | | **No. of alleles in Hap B** | | **No. of alleles in Hap C** | | **No. of alleles in Hap D** |
| Chr1 | 2814 | 2834 | 2777 | 1771 | | 1674 | | 1761 | | 1890 |
| Chr2 | 2916 | 2914 | 2851 | 1862 | | 1845 | | 1780 | | 1877 |
| Chr3 | 2019 | 2016 | 2035 | 1392 | | 1300 | | 1293 | | 1261 |
| Chr4 | 2242 | 2308 | 2349 | 1694 | | 1636 | | 1501 | | 1452 |
| Chr5 | 2538 | 2575 | 2598 | 1681 | | 1619 | | 1731 | | 1679 |
| Chr6 | 2162 | 2230 | 2277 | 1613 | | 1621 | | 1475 | | 1327 |
| Chr7 | 2366 | 2440 | 2364 | 1768 | | 1650 | | 1630 | | 1700 |
| Chr8 | 1641 | 1692 | 2229 | 1454 | | 1358 | | 1138 | | 1128 |
| Chr9 | 1954 | 2058 | 2093 | 1418 | | 1336 | | 1408 | | 1490 |
| Chr10 | 2104 | 2154 | 2210 | 1528 | | 1621 | | 1512 | | 1489 |
| Chr11 | 1902 | 1824 | 2405 | 1425 | | 1440 | | 1399 | | 1634 |
| Chr12 | 1790 | 1824 | 1912 | 1409 | | 1170 | | 1178 | | 1347 |
| Chr13 | 1858 | 1843 | 1924 | 1268 | | 1335 | | 1337 | | 1111 |
| Chr14 | 1522 | 1601 | 1663 | 1142 | | 1114 | | 1142 | | 1069 |
| Chr15 | 1321 | 1274 | 1426 | 950 | | 952 | | 969 | | 927 |
| Chr16 | 1522 | 1499 | 1520 | 1091 | | 1035 | | 1020 | | 925 |
| Anchored | 32,671 | 33,086 | 34,633 | **-** | | **-** | | **-** | | **-** |
| Total number | 34,699 | 35,221 | 90,752 | 23,466 | 22,706 | | 22,274 | | 22,306 | |

**Table S9 Genes number of the three *C*. *paliurus* genomes**
