# Supplementary material for: Whole-genome Duplication Reshaped Adaptive Evolution in A Relict Plant Species, Cyclocarya paliurus
Source: Genomics Proteomics Bioinformatics. 2023 Feb 11;21(3):455–69. doi: 10.1016/j.gpb.2023.02.001 (PMC10787019; doi:10.1016/j.gpb.2023.02.001)
Supplement: Supplementary Table S10 — BUSCO analysis of annotation completeness [file mmc57.docx]

| **Description** | **PA-dip** | | **PG-dip** | | **PA-tetra** | |
| --- | --- | --- | --- | --- | --- | --- |
|  | **Number** | **Percentage (%)** | **Number** | **Percentage (%)** | **Number** | **Percentage (%)** |
| Complete BUSCOs (C) | 1323 | 96.2 | 1323 | 96.2 | 1299 | 94.4 |
| Complete and single-copy BUSCOs (S) | 1203 | 87.5 | 1200 | 87.3 | 178 | 12.9 |
| Complete and duplicated BUSCOs (D) | 120 | 8.7 | 123 | 8.9 | 1121 | 81.5 |
| Fragmented BUSCOs (F) | 25 | 1.8 | 30 | 2.2 | 18 | 1.3 |
| Missing BUSCOs (M) | 27 | 2 | 22 | 1.6 | 58 | 4.3 |
| Total BUSCO groups searched | 1375 | 100 | 1375 | 100 | 1375 | 100 |

**Table S10 BUSCO analysis of annotation completeness**
