# Supplementary material for: Whole-genome Duplication Reshaped Adaptive Evolution in A Relict Plant Species, Cyclocarya paliurus
Source: Genomics Proteomics Bioinformatics. 2023 Feb 11;21(3):455–69. doi: 10.1016/j.gpb.2023.02.001 (PMC10787019; doi:10.1016/j.gpb.2023.02.001)
Supplement: Supplementary Table S12 — The expanded P450s families in theC. paliurus genome [file mmc59.docx]

| **Gene family** | **Number of genes** | **Gene ID** |
| --- | --- | --- |
| CYP71 | 1 | CpaM1st11714 |
| CYP82 | 6 | CpaM1st11392 |
|  |  | CpaM1st11409 |
|  |  | CpaM1st11411 |
|  |  | CpaM1st11418 |
|  |  | CpaM1st25816 |
|  |  | CpaM1st25822 |
| CYP89 | 16 | CpaM1st00845 |
|  |  | CpaM1st00847 |
|  |  | CpaM1st00850 |
|  |  | CpaM1st00853 |
|  |  | CpaM1st00858 |
|  |  | CpaM1st00863 |
|  |  | CpaM1st00864 |
|  |  | CpaM1st00866 |
|  |  | CpaM1st00872 |
|  |  | CpaM1st00873 |
|  |  | CpaM1st00874 |
|  |  | CpaM1st00881 |
|  |  | CpaM1st00885 |
|  |  | CpaM1st14090 |
|  |  | CpaM1st14091 |
|  |  | CpaM1st35816 |
| CYP706 | 18 | CpaM1st06185 |
|  |  | CpaM1st09353 |
|  |  | CpaM1st17603 |
|  |  | CpaM1st17613 |
|  |  | CpaM1st17625 |
|  |  | CpaM1st17635 |
|  |  | CpaM1st20465 |
|  |  | CpaM1st26335 |
|  |  | CpaM1st26346 |
|  |  | CpaM1st26520 |
|  |  | CpaM1st26526 |
|  |  | CpaM1st28507 |
|  |  | CpaM1st28509 |
|  |  | CpaM1st28521 |
|  |  | CpaM1st28524 |
|  |  | CpaM1st28527 |
|  |  | CpaM1st28613 |
|  |  | CpaM1st28615 |
| CYP727 | 1 | CpaM1st30617 |

**Table S12 The expanded P450s families in the *C*. *paliurus* genome**
