# Supplementary material for: Whole-genome Duplication Reshaped Adaptive Evolution in A Relict Plant Species, Cyclocarya paliurus
Source: Genomics Proteomics Bioinformatics. 2023 Feb 11;21(3):455–69. doi: 10.1016/j.gpb.2023.02.001 (PMC10787019; doi:10.1016/j.gpb.2023.02.001)
Supplement: Supplementary Table S13 — Comparison of growth indices between diploid and tetraploidC. paliurus seedling, one year after stumping [file mmc60.docx]

**Table S13 Comparison of growth indices between diploid and tetraploid *C*. *paliurus* seedling, one year after stumping**

| **Determination standard** | **Ploidy** | **Extremum** | **Mean ± SD** | **Character difference** | **Coefficient of variation** |
| --- | --- | --- | --- | --- | --- |
| Seedling height/cm | Diploid | 57.00–152.00 | 105.98 ± 19.38 | 11.38 | 0.22 |
|  | Tetraploid | 80.00–172.40 | 117.36 ± 17.51* |  | 0.15 |
| Ground diameter/mm | Diploid | 4.26–17.30 | 11.16 ± 2.49 | 6.3 | 0.22 |
|  | Tetraploid | 6.94–27.27 | 17.46 ± 4.37** |  | 0.25 |
| Compound leaf fresh weight/g | Diploid | 0.43–5.26 | 2.53 ± 1.01 | 1.74 | 0.40 |
|  | Tetraploid | 1.29–7.76 | 4.27 ± 1.58** |  | 0.37 |
| Compound leaf dry weight/g | Diploid | 0.14–1.96 | 0.77 ± 0.35 | 0.72 | 0.45 |
|  | Tetraploid | 0.39–3.22 | 1.49 ± 0.64** |  | 0.43 |
| Leaf moisture content | Diploid | 0.24–0.44 | 0.30 ± 0.03 | 0.05 | 0.10 |
|  | Tetraploid | 0.25–0.68 | 0.35 ± 0.08 |  | 0.23 |
| Compound leaf area/cm^2^ | Diploid | 43.31–503.39 | 253.10 ± 93.79 | 136.29 | 0.37 |
|  | Tetraploid | 86.39–636.79 | 389.39 ± 138.52** |  | 0.36 |
| Leaf specific weight/(g/cm^2^) | Diploid | 0.0016–0.0076 | 0.0031 ± 0.0010 | 0.0008 | 0.33 |
|  | Tetraploid | 0.0027–0.0056 | 0.0039 ± 0.0008 |  | 0.21 |
| Number of saw teeth | Diploid | 24–94 | 61 ± 12 | 3 | 0.20 |
|  | Tetraploid | 48–97 | 64 ± 13 |  | 0.20 |
| Blade length/cm | Diploid | 2.95–19.10 | 9.71 ± 2.25 | 3.06 | 0.23 |
|  | Tetraploid | 4.74–16.50 | 12.77 ± 2.86** |  | 0.22 |
| Blade width/cm | Diploid | 1.47–5.50 | 2.96 ± 0.78 | 1.77 | 0.26 |
|  | Tetraploid | 2.74–6.70 | 4.73 ± 1.09** |  | 0.23 |
| Blade aspect ratio | Diploid | 1.19–4.51 | 3.34 ± 0.50** | -0.6 | 0.15 |
|  | Tetraploid | 1.46–3.64 | 2.74 ± 0.47 |  | 0.17 |

*Note*: * * and * indicate significant differences at 0.01 and 0.05 levels, respectively. Character difference = Mean of tetraploid − diploid. SD, standard deviation.
