# Supplementary material for: Whole-genome Duplication Reshaped Adaptive Evolution in A Relict Plant Species, Cyclocarya paliurus
Source: Genomics Proteomics Bioinformatics. 2023 Feb 11;21(3):455–69. doi: 10.1016/j.gpb.2023.02.001 (PMC10787019; doi:10.1016/j.gpb.2023.02.001)
Supplement: Supplementary Table S15 — Summary of genetic variations in C. paliurus populations [file mmc62.docx]

| **Category** | **Diploid** | **Tetraploid** |
| --- | --- | --- |
| SNPs | 3,545,162 | 23,076,276 |
| Indels | 341,670 | 3,598,719 |
| Variants with effects on genes | 38,874 | 282,037 |
| SNPs that introduce stop codons | 145 | 1068 |
| SNPs that disrupt stop codons | 19 | 114 |
| SNPs that induce alternative splicing | 472 | 3237 |
| Indels located in genic regions | 3845 | 38,899 |
| Frameshift variants | 314 | 2088 |
| Nonsynonymous variants | 5503 | 35,241 |
| Synonymous variants | 3753 | 23,764 |

**Table S15 Summary of genetic variations in *C*. *paliurus* populations**
