# Supplementary material for: Whole-genome Duplication Reshaped Adaptive Evolution in A Relict Plant Species, Cyclocarya paliurus
Source: Genomics Proteomics Bioinformatics. 2023 Feb 11;21(3):455–69. doi: 10.1016/j.gpb.2023.02.001 (PMC10787019; doi:10.1016/j.gpb.2023.02.001)
Supplement: Supplementary Figure S24 — Co-expression modules identified by weighted gene co-expression network analysis (WGCNA) [file mmc25.pdf]

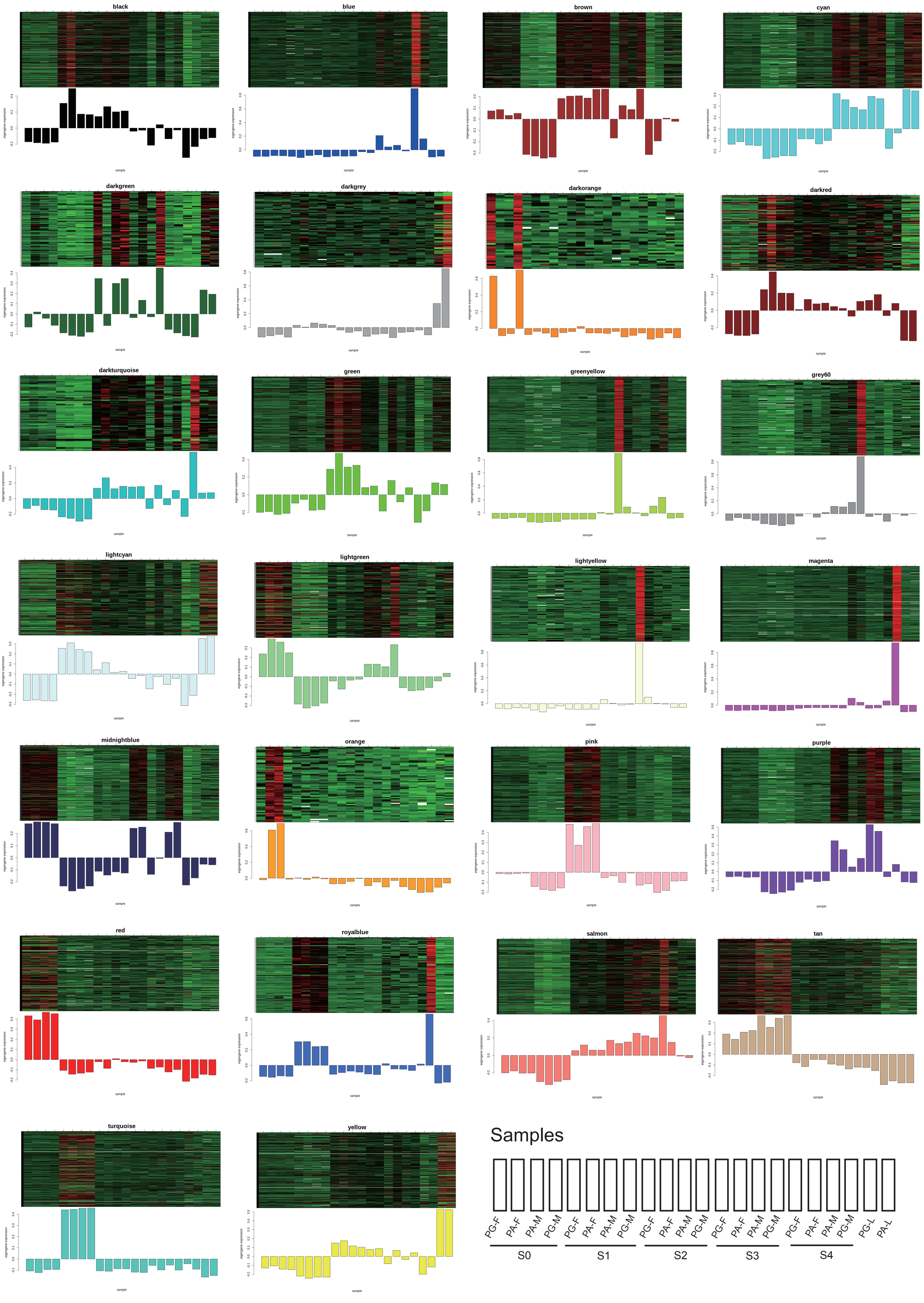
black
blue
brown
cyan
darkgreen
darkgrey
darkorange
darkred
darkturquoise
green
greenyellow
grey60
lightcyan
lightgreen
lightyellow
magenta
midnightblue
orange
pink
purple
red
royalblue
salmon
tan
turquoise
yellow
eigengene expression
sample
Samples
PG-F PA-F PA-M PG-M PG-F PA-F PA-M PG-M PG-F PA-F PA-M PG-M PG-F PA-F PA-M PG-M PG-F PA-F PA-M PG-M PG-L PA-L
S0
S1
S2
S3
S4
